# Magnesium-catalysed nitrile hydroboration†
†Electronic supplementary information (ESI) available: Experimental procedures and full characterisation data, details of the X-ray analyses of compounds **1–5**, protocols and data associated with the kinetic analyses and NMR spectra. CCDC 1018705–1018709. For ESI and crystallographic data in CIF or other electronic format see DOI: 10.1039/c5sc03114a


**DOI:** 10.1039/c5sc03114a

**Published:** 2015-10-20

**Authors:** Catherine Weetman, Mathew D. Anker, Merle Arrowsmith, Michael S. Hill, Gabriele Kociok-Köhn, David J. Liptrot, Mary F. Mahon

**Affiliations:** a Department of Chemistry , University of Bath , Claverton Down , Bath , BA2 7AY , UK . Email: msh27@Bath.ac.uk

## Abstract

A β-diketiminato *n*-butylmagnesium complex is presented as a selective precatalyst for the reductive hydroboration of organic nitriles with pinacolborane (HBpin). Stoichiometric reactivity studies indicate that catalytic turnover ensues through the generation of magnesium aldimido, aldimidoborate and borylamido intermediates, which are formed in a sequence of intramolecular nitrile insertion and inter- and intramolecular B–H metathesis events. Kinetic studies highlight variations in mechanism for the catalytic dihydroboration of alkyl nitriles, aryl nitriles bearing electron withdrawing (Ar(EWG)CN) and aryl nitriles bearing electron donating (Ar(EDG)CN) substitution patterns. Kinetic isotope effects (KIEs) for catalysis performed with DBpin indicate that B–H bond breaking and C–H bond forming reactions are involved in the rate determining processes during the dihydroboration of alkyl nitriles and Ar(EDG)CN substrates, which display divergent first and second order rate dependences on [HBpin] respectively. In contrast, the hydroboration of Ar(EWG)CN substrates provides no KIE and HBpin is not implicated in the rate determining process during catalysis. Irrespective of these differences, a common mechanism is proposed in which the rate determining steps are deduced to vary through the establishment of several pre-equilibria, the relative positions of which are determined by the respective stabilities of the dimeric and monomeric magnesium aldimide and magnesium aldimidoborate intermediates as a result of adjustments to the basicity of the nitrile substrate. More generally, these observations indicate that homogeneous processes performed under heavier alkaline earth catalysis are likely to demonstrate previously unappreciated mechanistic diversity.

## Abstract 2

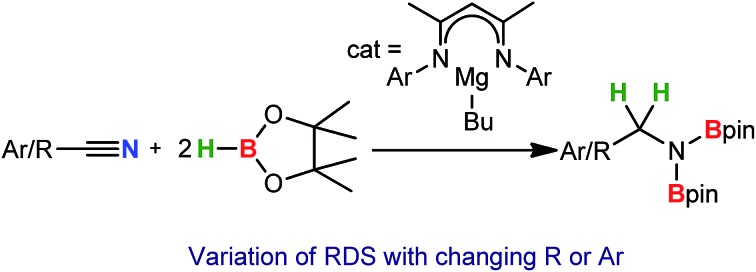
A β-diketiminato *n*-butylmagnesium complex is presented as a selective precatalyst for the reductive hydroboration of organic nitriles with pinacolborane (HBpin).

## Introduction

The reduction of organic nitriles to primary amines is an essential component of many industrial processes (*e.g.* the production of dyes, polyesters, agrochemicals and as precursors for pharmaceutical compounds).^1^ Catalytic nitrile hydrogenation may be achieved under heterogeneous conditions. These latter processes, however, are typically poorly selective and also result in the unwanted formation of imine and secondary amine side products at the high temperatures required.^2^ While their reliance on the use of poorly abundant and/or toxic precious metals is a further indicator of the unsustainability of these heterogeneous systems, it is notable that the development of well-defined solution-phase catalysis is limited to a handful of reports which deploy similarly expensive species derived from heavy precious metals.^3^,^4^ Although the reduction of nitriles may also be achieved through the use of stoichiometric quantities of main group reducing agents such as LiAlH_4_ and NaBH_4_,^5^ the flammable nature of these reagents, coupled with large amounts of inorganic waste by-products, again renders them unattractive. More recent reports have shown that reduction may be accomplished using amine borane reagents,^6^ whilst other novel hydrogenation methods have exploited the use of catalytic amounts of ‘frustrated’ Lewis pairs to provide the first metal-free systems to reduce nitriles, albeit under rather energetic (120 °C) reaction conditions.^7^

Whilst nitrile hydrogenation provides a direct route to the desired amine product, reductive hydrosilylation or hydroboration can be advantageous in their provision of further functionality to the resultant amine.^8^ Although the catalytic hydroboration of a wide range of multiply-bonded substrates has been achieved, only a handful of nitrile hydroboration reactions have been devised and all but one of these previous reports described non-catalysed H–B addition and required the use of more activated and less discriminating borane reagents.^9^ A unique case of a catalysed addition, therefore, has been provided by Nikonov's report of the catalytic hydroboration of nitriles using 5 mol% of the Mo(iv) imido–hydrido complex (**I**) with catecholborane (HBcat).^10^ With this system acetonitrile and benzonitrile were reduced to the 1,1-bis(boryl)amine products, which were themselves shown to undergo chemoselective coupling with aldehydes, R′C(O)H, to afford imines RCH_2_N=C(H)R′.
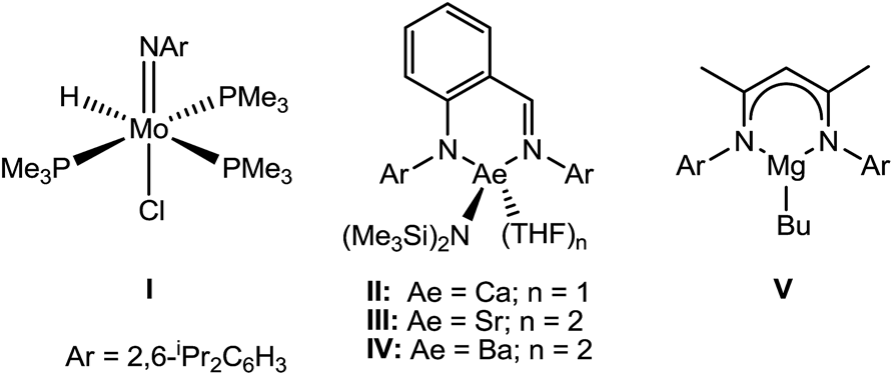


**Scheme 1:**
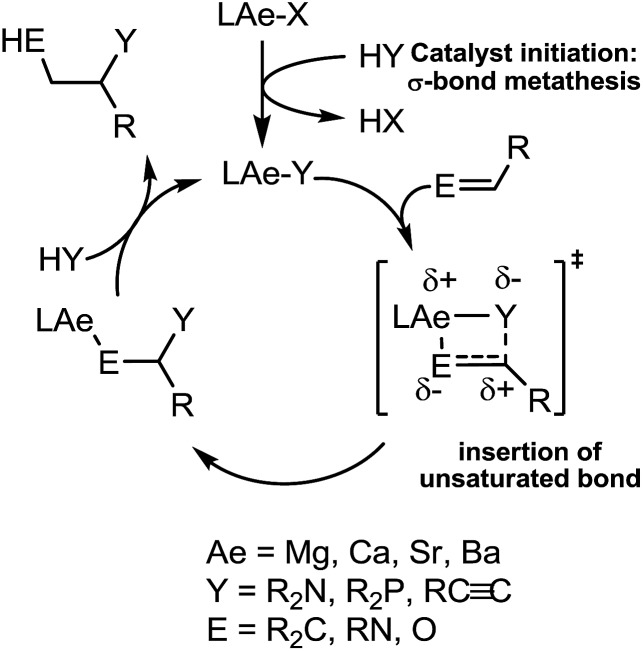


**Scheme 2:**
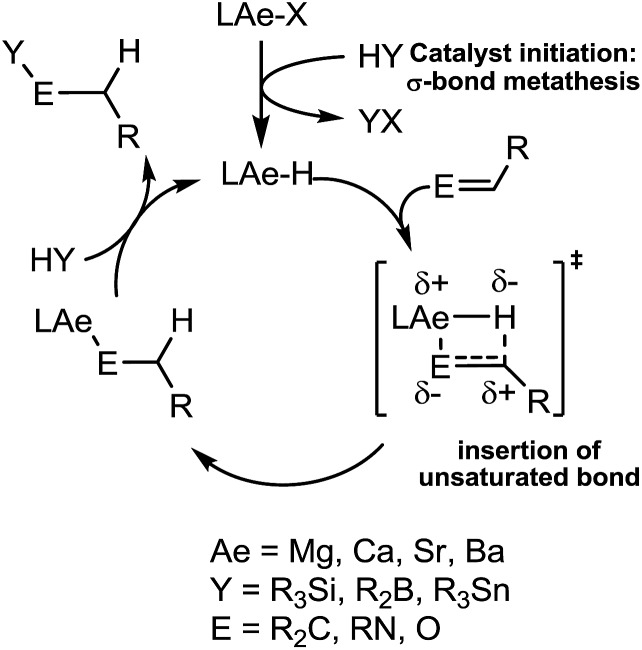


Our own research has focussed on the development of a homogeneous catalytic chemistry for complexes, LAeX (Ae = Mg, Ca, Sr and Ba; L = unreactive spectator ligand; X = reactive substituent), derived from the heavier alkaline-earth elements.^11^ The negligible toxicity and high natural abundance of calcium and magnesium (the fourth and sixth most abundant lithospheric elements respectively) in particular designate species of this type as environmentally benign and sustainable. Although these are primary motivating factors for the development of this chemistry, an additional major concern is a deconvolution of basic reactivity patterns for this relatively understudied family of elements. Based upon an immutable +2 oxidation state and effectively ionic ligand and substrate binding under catalytic conditions, a level of ‘lanthanide mimetic’ behaviour was initially assumed. Consequently, a wide variety of heterofunctionalisation catalyses, predicated on sequences of regioselective and polarisation-dependent sigma-bond metathesis and insertion events have now been described (Schemes 1 and 2).^11^ A majority of detailed studies have focussed on the intramolecular hydroamination/cyclisation of aminoalkenes as an appropriate baseline reaction that is well-precedented in homogeneous 4f-element centred catalysis.^12^,^13^ Within this one reaction type alone, distinct variations which occur with changing group 2 atomic weight have been rationalised as a consequence of the substantial adjustments to ionic radius and cation charge density on descending the group. While an expectation of some rate variation with alkaline earth identity may appear retrospectively obvious, it is also apparent that a generalised framework accounting for the intrinsic or relative reactivity of the individual elements is yet to emerge. Recent work by Sarazin and Carpentier,^14^ for example, employing the anilido-imine species **II–IV** has indicated a general increase in catalytic hydroamination capability with increasing group 2 atomic weight (*i.e.* Ca < Sr < Ba) whereas our own studies of the homoleptic bis(trimethylsilyl)amides [Ae{N(SiMe_3_)_2_}_2_]_2_ [Ae = Mg, Ca, Sr, Ba] have evidenced more subtle kinetic dependences upon the identity and characteristics of the Ae^2+^ cation.^11^ In such cases we have reasoned that the facility for reaction is determined by an intricate interplay of factors of which complex nuclearity and access to the group 2 centre, in concert with the basicity of the pre-catalytic reagent and an ability to polarise in-coming substrate molecules, are essential to successfully traverse the various transition states involved in the catalytic process.

**Scheme 3:**
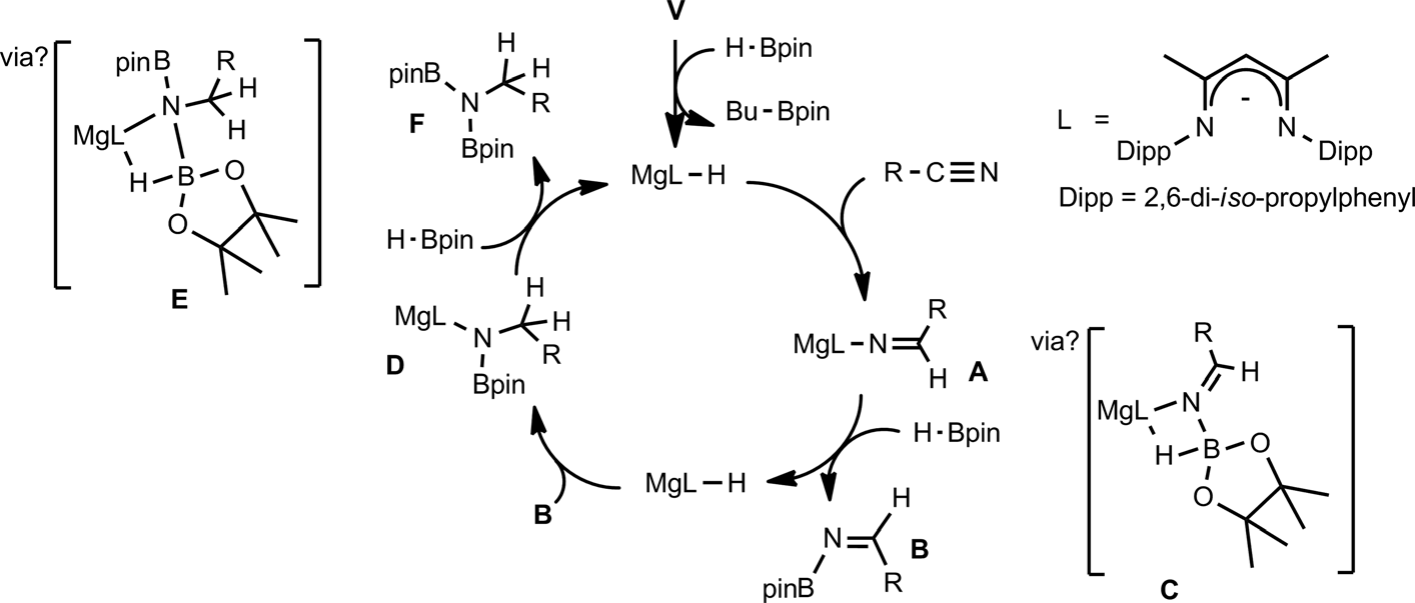
Prototype mechanism for dihydroboration of nitriles.

We have previously reported the use of the magnesium alkyl precatalyst (**V**) for the hydroboration of aldehydes and ketones,^15^ as well as pyridines,^16^ aldimines, ketimines^17^ and CO_2_ (ref. 18*a*) with HBpin. Most recently we have described a unique case of isonitrile dihydroboration through use of the same borane and magnesium reagents, which provides access to 1,2-diborylated amines.18*b* In all cases this reactivity was deduced to be derived from the rapid and unobservable generation of magnesium hydride species through Mg–C/B–H σ-bond metathesis and a sequence of polarised insertion and further metathesis steps (*cf.*Scheme 2). In common with a recent report of magnesium-catalysed ester reduction with HBpin,19*a* these reactions are also observed to occur through the assembly of alkoxy- or amido-hydrido borate derivatives, in which the hydride reducing equivalent is already present in the proposed catalyst resting state. During the course of these studies we have previously noted that cyanopyridines underwent double hydroboration of the nitrile C–N triple bond to form *N*,*N*-(diboryl)aminopyridines.^16^ The reaction sequence depicted in Scheme 3 may, thus, be envisaged as a generalised means to effect a similar dihydroboration of organic nitriles. In this case the formation of the 1,1-diborylated amine product (**F**) requires the consumption of two molecules of HBpin and the intermediacy of an initial magnesium-bound imide (**A**), a borylated imine **(B**) as the product of initial B–H/Mg–N metathesis and an amide species (**D**) formed by formal Mg–H insertion of the borylimine (**B**). The additional complexity introduced by the requirement for two-fold nitrogen functionalisation, thus, raises a number of issues regarding validity of such a stepwise process and the identity of other potential intermediates such as the borate species shown as **C** and **E** shown in Scheme 3. (*vide infra*).

With these broader considerations in mind we now describe a magnesium-centred protocol for the hydroboration of organic nitriles with the unactivated borane reagent pinacolborane (HBpin). Our selection of a single β-diketiminato magnesium precatalyst (**V**), which is stable to both Schlenk-type redistribution equilibria and to chemical degradation under conditions of the catalysis, allows for an assessment of the effects of gradual substrate adjustment which suggests that every reaction must be treated on its merits.

## Results

### Catalytic reaction scope

An initial catalytic reaction using 10 mol% of **V** with respect to one equivalent of propionitrile with 2 molar equivalents of HBpin at room temperature evidenced slow consumption of the reagents while heating of an identical reaction mixture at 60 °C provided quantitative conversion to the 1,1-bis(boryl)amine product within 30 minutes. The clean formation of this new species was clearly apparent through the emergence of a (2H) triplet methylene resonance in the ^1^H NMR spectrum at *δ* 3.42 ppm synchronous with a new singlet signal in the ^11^B NMR spectrum at *δ* 29.5 ppm. In contrast an analogous reaction performed without the addition of **V** evidenced less than 5% consumption of the propionitrile substrate when heated at 60 °C for 16 hours. Encouraged by this result the conditions of the catalytic study were extended to the successful di-hydroboration of the range of alkyl and aryl nitriles summarised in Table 1.

**Table 1:** Catalytic dihydroboration of organonitriles with HBpin and 10 mol% **V**

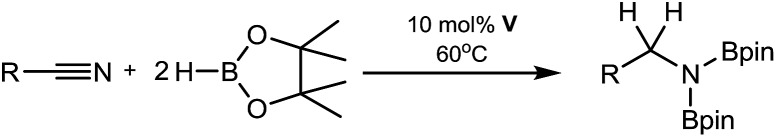				
Entry	R	Time (h)	NMR yield^*a*^ (%)	Isolated yield^*a*^ (%)
1	Et	0.5	>99	70
2	*i*-Pr	1	93	96
3	*t*-Bu	5.5	97	54
4	Cy	1	94	75
5	C_6_H_5_	12	92	56
6	*o*-(Me)C_6_H_4_	15	86	73
7	*m*-(Me)C_6_H_4_	15	87	81
8	*m*-(F)C_6_H_4_	14	88	57
9	*m*-(MeO)C_6_H_4_	15	90	60
10	*p*-(Me)C_6_H_4_	13	91	72
11	*p*-(F)C_6_H_4_	12	94	61
12	*p*-(Cl)C_6_H_4_	12	93	59
13	*p*-(CF_3_)C_6_H_4_	12.5	89	76
14	*p*-(MeO)C_6_H_4_	13.5	88	58
15	Ph_2_CH	30	75	43

^*a*^All NMR reactions were carried out in C_6_D_6_ whilst scale-up reactions were carried out in toluene then isolated from hexanes.

**Fig. 1:**
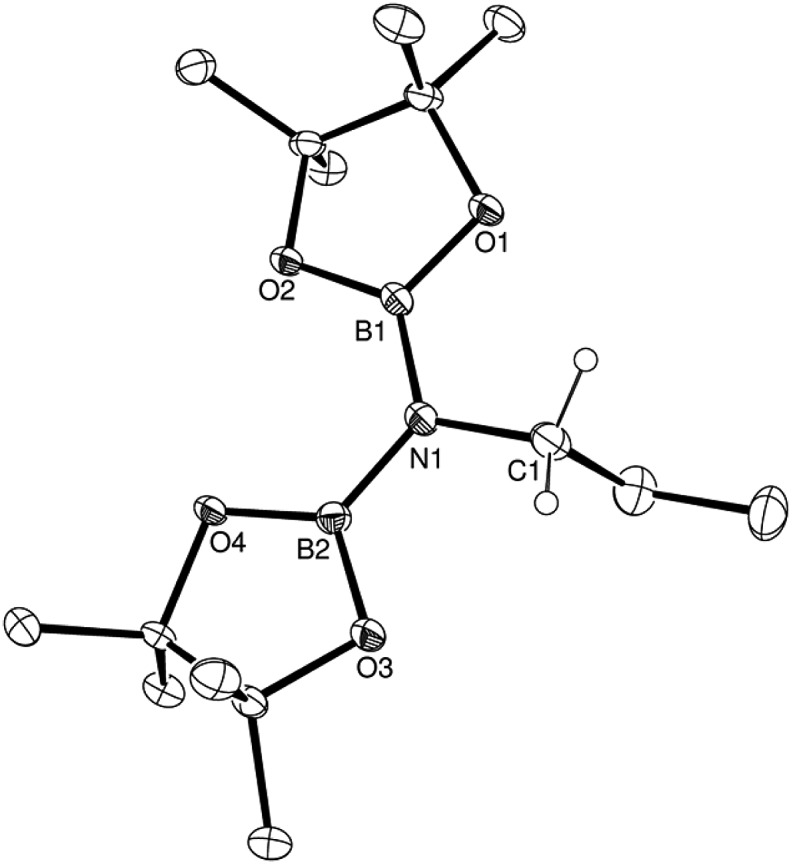
ORTEP representation of the structure of compound **1** (30% probability ellipsoids). Hydrogen atoms, except for those attached to the C1 methylene carbon centre, are omitted for clarity.

In common with our earlier reports of the magnesium-catalysed hydroboration of aldehydes, ketones, imines and isonitriles,^15^–^17^,18b any increase in the overall steric demands of the substrate resulted in a longer reaction time. This is most readily appreciated through consideration of the effects of increasing steric demands upon substitution of the N≡C-bound carbon centre of aliphatic nitriles wherein a 10-fold increase in reaction time is observed across the transition from propionitrile (entry 1) to iso-propylnitrile (entry 2) to *tert*-butylnitrile (entry 3). Aryl nitriles (entries 5–15) also provided clean reactions with no side products, but with markedly longer reaction times. Notably varied reaction times were also required with changing aryl substituent patterns. Although in the case of *o*-tolunitrile (entry 6) the slower reaction may again be attributed to an increase in substituent steric hindrance proximal to the reaction centre, any minor variability across the range of *meta*-and *para*-substituted arylnitriles is more realistically attributed to electronic adjustments across the pi-conjugated substrate or intermediate structure (*vide infra*). The identities of the products from this catalysis were confirmed by ^1^H, ^11^B and ^13^C NMR spectroscopy and a single crystal X-ray diffraction analysis performed upon the product (compound **1**) isolated from the catalytic dihydroboration of propionitrile. The outcome of this latter experiment is shown in Fig. 1, while selected bond lengths and angles and details of the X-ray analysis are provided in Tables 2–4 respectively. The N1–C1 bond length of 1.497(2) Å is consistent with the expected twofold reduction of the nitrile while the N1–C1–C2 angle of 109.1° is a clear indication of sp^3^ hybridisation at the C1 carbon atom.

**Table 2:** Selected bond lengths (Å) for compounds **1–5**

	**1**	**2**	**3**	**4**	**5**
N1–B1	1.423(2)	—	—	1.572(2)^*a*^	—
N1–B2	1.430(2)	—	—	—	—
N1–C1	1.497(2)	—	—	—	—
Mg1–N1	—	2.0957(16)	2.1003(16)	2.0334(13)	2.033(5)^*b*^
Mg1–N2	—	2.0931(17)	2.0840(16)	2.0361(13)	2.057(4)^*c*^
Mg1–N3	—	2.1062(16)	2.0951(16)	2.0825(13)	1.996(6)^*d*^
Mg1–O1	—	—	—	1.9714(11)	1.974(4)^*e*^
N3–C30	—	1.263(2)	1.271(2)	1.266(2)	1.188(8)^*f*^ ^,^^*g*^

^*a*^N3–B1.

^*b*^Mg2–N4 2.046(4).

^*c*^Mg2–N4 2.0467(4).

^*d*^Mg1–N5.

^*e*^Mg2–O2 1.953(4).

^*f*^N(5)–C(30).

^*g*^N6–C79 1.114(7).

**Table 3:** Selected bond angles (°) for compounds **1–5**

	**1**	**2**	**3**	**4**	**5**
B1–N1–B2	126.15(15)	—	—	—	—
B1–N1–C1	117.15(14)	—	—	—	—
B2–N1–C1	116.41(14)	—	—	120.10(13)^*a*^	—
N2–Mg1–N1	—	90.19(6)	90.88(6)	95.26(5)	93.76(18)^*b*^
N1–Mg1–N3	—	119.63(6)	124.69(7)	132.20(6)	105.7(2)^*c*^ ^,^^*d*^
N2–Mg1–N3	—	122.98(7)	117.31(6)	115.45(5)	107.3(2)^*e*^ ^,^^*f*^
C30–N3–Mg1	—	136.56(13)	137.55(13)	150.55(12)	—
O1–Mg1–N3	—	—	—	71.91(5)	103.31(19)^*g*^ ^,^^*h*^

^*a*^B1–N3–C30.

^*b*^N3–Mg2–N4 93.44(18).

^*c*^N1–Mg1–N5.

^*d*^N3–Mg2–N6 103.53(19).

^*e*^N2–Mg1–N5.

^*f*^N4–Mg2–N6 105.5(2).

^*g*^O1–Mg1–N5.

^*h*^O2–Mg2–N6 97.01(13).

**Table 4:** X-ray crystallographic data for compounds **1–5**

	**1**	**2**	**3**	**4**	**5**
Empirical formula	C_15_H_31_B_2_NO_4_	C_37_H_49_MgN_3_O	C_74_H_98_Mg_2_N_6_O_2_	C_40_H_64_MgBN_3_O_2_	C_102_H_132_BMg_2_N_6_O_2_
FW (g mol^–1^)	311.03	1152.20	1152.20	654.06	1533.57
Crystal system	Triclinic	Monoclinic	Monoclinic	Monoclinic	Monoclinic
Space group	*P*1	*P*2_1_/*n*	*P*2_1_/*n*	*P*2_1_/*c*	*Cc*
*a* (Å)	6.3072(1)	12.7410(2)	22.7470(2)	13.7058(2)	12.3279(5)
*b* (Å)	12.4643(3)	12.9480(2)	13.2640(1)	13.1659(2)	30.1877(9)
*c* (Å)	12.5184(3)	20.7600(3)	24.9170(2)	22.9509(3)	26.5175(11)
*α* (°)	101.1406(10)	90	90	90	90
*β* (°)	100.1735(10)	92.575(1)	115.512(1)	96.1770(10)	102.622(2)
*γ* (°)	103.8021(12)	90	90	90	90
Density (Mg m^–3^)	1.133	1.118	1.128	1.055	1.058
*Z*	2	4	4	4	4
*μ* (Mo Kα) (mm^–1^)	0.078	0.083	0.084	0.077	0.074
Reflections collected	16 086	58 058	113 992	76 184	35 060
Independent reflections	3190	7818	15 492	9431	14 727
*R*_int_	0.0465	0.0658	0.0685	0.0814	0.1401
*R*_1_, w*R*_2_ [*I* > 2*σ*(*I*)]	0.0495, 0.1087	0.0569, 0.1247	0.0561, 0.1192	0.0498, 0.1103	0.0771, 0.1602
*R* Indices (all data)	0.0613, 0.1137	0.0837, 0.1347	0.1025, 0.1405	0.0894, 0.1273	0.1605, 0.1978

### Stoichiometric reactions

**Fig. 2:**
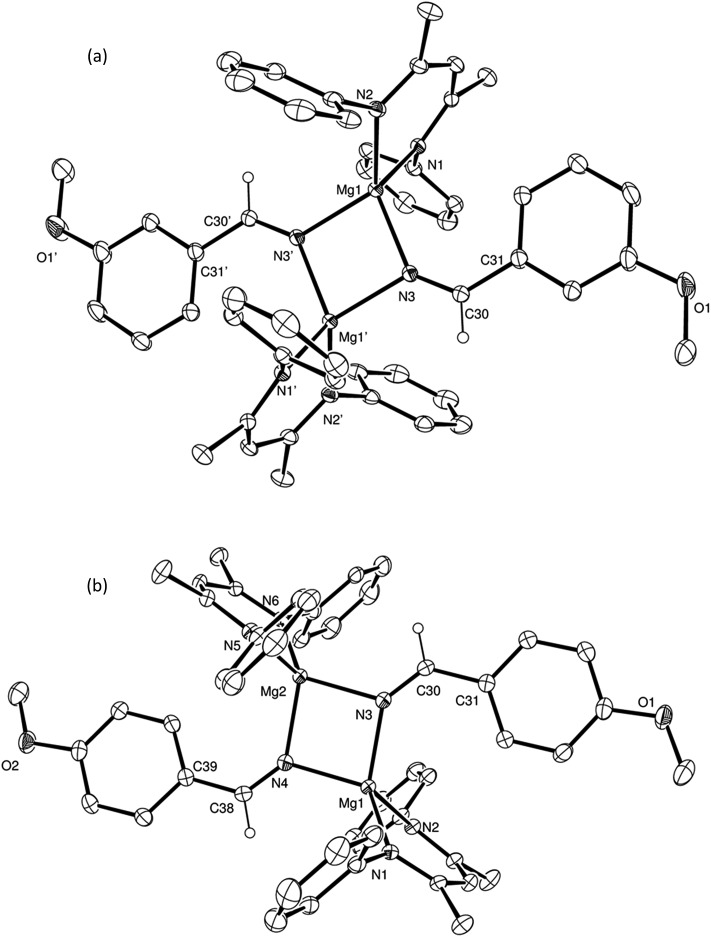
ORTEP representations of (a) compound **2** and (b) compound **3** (30% probability ellipsoids). Hydrogen atoms, except for those attached to the C(30) and, for **3**, the C(38) methine carbon centres, and iso-propyl groups are omitted for clarity. Symmetry transformations used to generate equivalent (′) atoms in (b) –*x* + 1, –*y* + 1, –*z* + 1.

To provide further insight into the course of these successful catalytic reactions and the viability of intermediate species such as those shown as **A–E** in Scheme 3, a series of stoichiometric reactions were undertaken. Monitoring of NMR scale reactions performed with *t*-BuCN was particularly informative due to the ready discrimination of the diagnostic singlet *tert*-butyl resonances in the ^1^H NMR spectra. We have earlier reported that **V** and HBpin react to form a magnesium hydride/borohydride species along with BuBpin within minutes at room temperature.^16^ Repetition of this initial reaction with subsequent addition of an equimolar equivalent of *t*-BuCN provided complete C≡N insertion into the magnesium hydride bond, which was clearly apparent after heating for 12 hours at 60 °C through the formation of a single new compound designated as an aldimide derivative analogous to the species **A** depicted in Scheme 3. The production of this species was clearly evidenced by the emergence of a downfield singlet (1H by integration) resonance at *δ* 7.84 ppm assigned as the aldimide methine signal and a set of resonances associated with a single new β-diketiminate ligand environment in the ^1^H NMR spectrum (see Scheme S1†). Although attempts to isolate this aliphatic aldimide species were unsuccessful, the veracity of this deduction was supported by subsequent analogous reactions performed with *m*- and *p*-MeOC_6_H_4_CN. While both of the resultant compounds (**2** and **3**) again displayed characteristic 1H aldimide methine singlet resonances (**2**, *δ* 8.63; **3***δ* 8.58 ppm) in their respective ^1^H NMR spectra, in these latter cases crystallisation from benzene solutions also provided samples of both compounds suitable for single crystal X-ray diffraction analysis. The results of these analyses are presented in Fig. 2a and b while selected bond lengths and angles and details of the X-ray analyses are provided in Tables 2–4 respectively. Although compounds **2** and **3** are the first magnesium aldimide complexes to be characterised in the solid state, their structures are otherwise analogous to a previously described calcium benzaldimide derivative supported by the identical β-diketiminate ligand.^20^ As was the case for this heavier congener, the structures of compounds **2** and **3** are dimeric with bridging Mg–N–Mg interactions provided by the aldimide ligands. Whereas the asymmetric unit of compound **2** comprises half of a dimer which straddles a crystallographic inversion centre, each half of the dimeric unit of compound **3** is unique. The gross features of both structures are, however, very similar wherein the magnesium centres are bridged by unsymmetrical Mg–N–Mg interactions. The magnesium to aldimide nitrogen bond lengths in **2** and **3** [**2**: 2.1062(16), 2.0918(16); **3**: 2.0840(16), 2.0882(16), 2.0951(16), 2.0974(16) Å] are shorter than the magnesium to amide contacts observed within topologically related dimeric magnesium benzylamide and pyrollidide derivatives [2.1251(16) and 2.117(2) Å respectively], both of which contain four-coordinate magnesium centres supported by the identical β-diketiminate ligand.^21^ The transition from formal sp^3^ to sp^2^ hybridisation at the bridging nitrogen centres in comparison to these previously described compounds is also reflected by the more obtuse Mg–N–Mg bond angles [90.73(6) and 91.58(9)° *versus* (**2**) 95.51(6)°, (**3**) 95.26(6) and 95.27(6)°] within compounds **2** and **3**. As previously highlighted in Harder and co-workers' discussion of their calcium benzaldimide species,^20^ the planes described by the methoxyphenyl rings of both compounds **2** and **3** are virtually co-planar with the planes formed by the magnesium and the aldimide nitrogen and sp^2^ carbon centres. A similar in-plane conformation is also a common feature of dimeric diorganoaluminum benzaldimide species, [R_2_AlN=C(Ph)H]_2_,^22^ and has been calculated for the model lithium compound, [LiN=CH_2_]_2_, to lie some 17 kcal mol^–1^ lower in energy than the alternative conformation in which the methylene unit is perpendicular to the Li_2_N_2_ plane.^23^ In the current case, the angles subtended by the aryl rings and the various Mg–N–C(Ar)H planes within compounds **2** and **3** (**2**: 0.7°; **3**: 11.3, 10.9°) indicate that this stabilisation is further enhanced by delocalisation across the aryl substituents. Although such data should be treated with caution due to the possibility of solid-state crystal packing and dispersion effects, we ascribe the greater co-planarity of **2** in comparison to **3** to the mesomeric influence of the respectively stabilizing 3-methoxy- and destabilising 4-methoxyphenyl substituents. While only providing a minor solid-state effect, we suggest that modulation of aldimide resonance stabilisation is a significant factor during the magnesium-catalysed hydroboration of the range of aryl nitriles listed in Table 1 (entries 5–14) (*vide infra*).

**Fig. 3:**
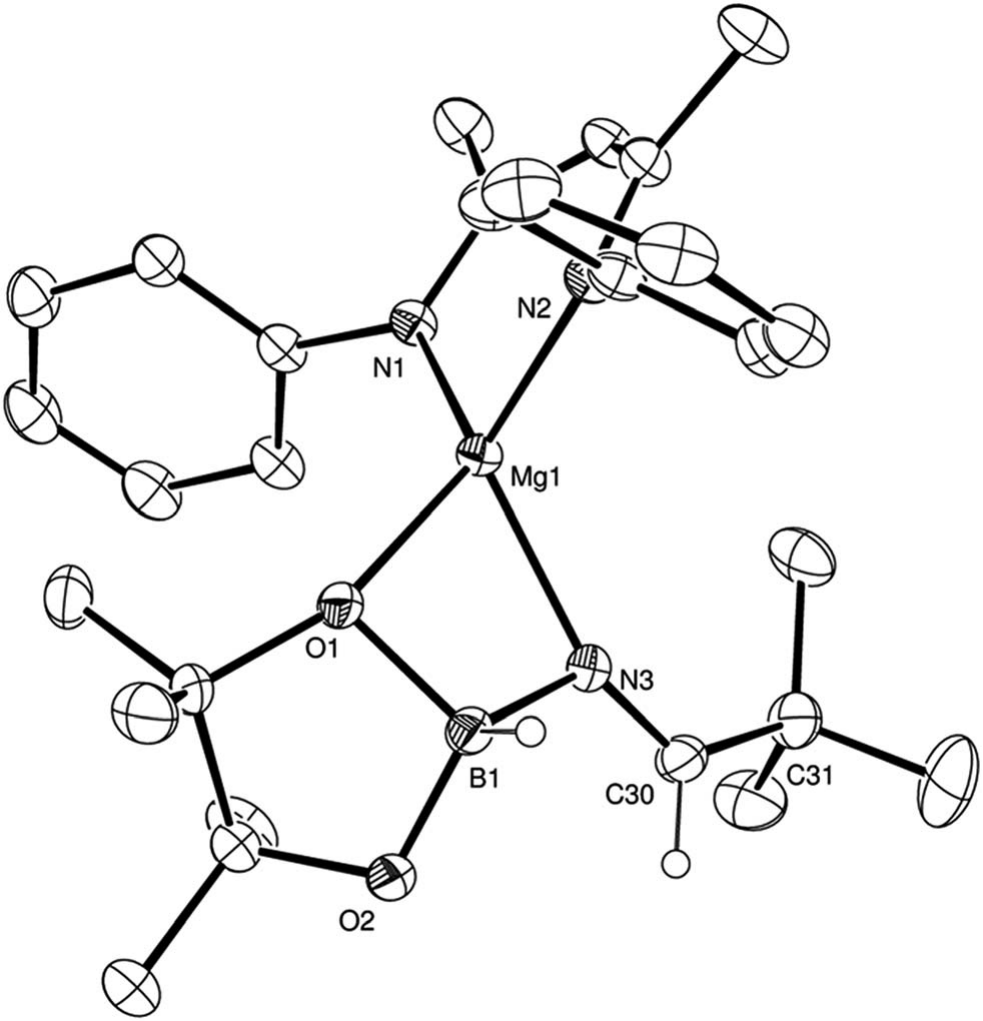
ORTEP representation of compound **4** (30% probability ellipsoids). Hydrogen atoms apart from those attached to B1 and C30 and iso-propyl groups are omitted for clarity.

Counter to the expectation illustrated in Scheme 3, addition of a further equivalent of HBpin to the imide product generated in the reaction with *t*-BuCN did not provide any initial evidence of B–H/Mg–N metathesis and production of a borylated imine derivative (shown as **B** in Scheme 3). Rather, this procedure yielded a single new species, assigned as a magnesium aldimidoborohydride (**C** in Scheme 3), characterised by a resonance at *δ* 9.27 ppm in the ^11^B NMR spectrum in which the integrity of the B–H bond was confirmed by a ^1^*J*_BH_ coupling of 105 Hz; a value that is significantly reduced from the three-coordinate HBpin reagent (^1^*J*_BH_ = 175 Hz) and more typical of four-coordinate boron.^24^ Scale up of this reaction in hexane and storage at –30 °C to avoid conversion to the boryl amide species (*vide infra*) allowed the isolation of the aldimidoborohydride derivative, compound **4**, which was fully characterised by multinuclear NMR spectroscopy and a further single crystal X-ray experiment. The results of this latter analysis are presented in Fig. 3 and selected bond lengths and angles and details of the X-ray analysis are provided in Tables 2–4 respectively. The monomeric structure displays a distorted tetrahedral geometry about the magnesium atom with coordination from the aldimidoborohydride ligand provided by interactions with the imide nitrogen centre and one of the oxygen atoms of the pinacolate unit of the anion. Although the formation of borohydride intermediates has been previously implied from our NMR studies of both carbonyl and imine hydroboration^15^,^17^ and Sadow's ester reduction catalysis,19*a* the structure of compound **4** provides the first crystallographic evidence for the intermediacy of borohydride intermediates during any magnesium-mediated hydroboration catalysis. The C(3)–N(3) distance (1.226(2) Å) of the formal C=N double bond, although shorter than the aldimide C=N distances, comprising 2-coordinate nitrogen, within compounds **2** and **3** (**2**: 1.263(2); **3**: 1.262(2), 1.271(2) Å), is closely comparable to previously reported, albeit exclusively transition metal-coordinated, imine-derived borate anions.^25^

Samples of compound **4** in C_6_D_6_ were observed to undergo continued reaction on standing at room temperature over a period of 12 hours; clearly observed by monitoring of the time-resolved ^1^H NMR spectra through the disappearance of the downfield 1H aldimido proton signal and the simultaneous appearance of a new singlet resonance at *δ* 3.35 ppm with an ultimate 2H intensity by integration. The corresponding ^11^B NMR spectra over this period evidenced a small downfield shift to *δ* 6.3 ppm with loss of the doublet signal multiplicity. We suggest that these observations are a consequence of an intramolecular hydride shift resulting in reduction of the aldimide residue and production of a magnesium borylamide derivative directly analogous to species **D** shown in Scheme 3. Addition of a final equivalent of HBpin yielded the desired dihydroboration product, depicted as species **F** in Scheme 3. Conversion to this latter product, with regeneration of the initial magnesium hydride species, however, required heating at 60 °C obviating any observation of borohydride intermediates akin to species **E** in Scheme 3.

Notably, analogous addition of one equivalent of HBpin to solutions of compounds **2** and **3** provided no evidence for similar borate formation and provided resonances consistent with the persistence of the unreacted starting compounds. DOSY NMR analysis of these reactions also evidenced no change in the solution diffusion coefficient attributed to the dimeric species even with increasing temperature and the addition of further equivalents of HBpin. We attribute this observation to the additional conjugative stability provided to the dimeric unit by the co-planarity of the 3- and 4-methoxyphenyl substituents with the C–N–Mg linkage.

**Fig. 4:**
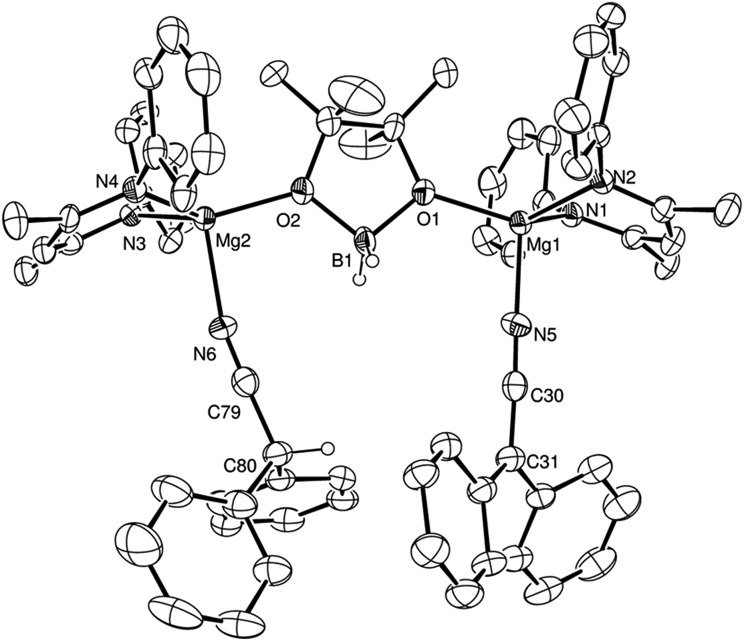
ORTEP representation of compound **5** (30% probability ellipsoids). Iso-propyl groups and hydrogen atoms other than those attached to B1 and C80 have been removed for clarity.

Isolated samples of compounds **2–4** could themselves be utilised as precatalysts for the hydroboration of *m*- and *p*-MeOC_6_H_4_CN and *t*-BuCN respectively. These reactions provided conversions and reaction times that were broadly commensurate with the reactions initiated by **V** (Table 1; entries 9, 14 and 3), indicating the possible intermediacy of these aldimide and aldimidoborohydride species during the course of each catalysis. Further stoichiometric reactions of alkyl nitriles bearing less sterically demanding substituents than *tert*-butyl were found to take place with insufficient discrimination to provide useful mechanistic information. A similar reaction of compound **V** with Ph_2_CHCN and HBpin, however, highlighted the potential for more complex behaviour during magnesium-catalysed nitrile hydroboration. Upon immediate addition of the reagents, bubbles of gas were observed and single crystals of a new compound (**5**) were isolated from the unstirred reaction mixture. Although this new species was insufficiently soluble to allow characterisation by solution-state NMR spectroscopy, a single crystal X-ray diffraction analysis revealed compound **5** as the unusual dinuclear magnesium complex shown in Fig. 4. Selected bond length and angle data and details of the X-ray analysis are again provided in Tables 2–4 respectively. The alkaline earth centres of compound **5** are connected through bridging interactions provided by both oxygen atoms of a [H_2_Bpin]^–^ anion, in a manner reminiscent of that observed in both a previously reported trimeric calcium species, [LCa(H_2_Bpin)]_3_ (where L is as defined in Scheme 3),^26^ and a μ-Mg–H–Mg and O–B–O bridged β-diketiminato magnesium compound the connectivity of which was established through a low resolution (*R*_1_ = 10.31%) X-ray diffraction analysis.^16^ Although each of the magnesium atoms displays a similar *pseudo*-tetrahedral N_3_O-coordination sphere, with ligation provided by the bridging borate and a single β-diketiminate anion, the nature of the fourth nitrogen-centred ligand differs across the two Mg centres of the molecule. Whereas Mg(1) is coordinated by a molecule of diphenylacetonitrile, the coordination sphere of Mg(2) is completed by a diphenylketeniminate anion, generated by deprotonation of the nitrile starting material. Magnesium and barium derivatives of the identical diphenylketeniminate anion are precedented by complexes in which the alkaline earth centres were further coordinated by sterically demanding bis(imino)acenaphthene (Dipp-BIAN) radical anions, in which case the magnesium complex was prepared by deprotonation of diphenylacetonitrile by [(Dipp-BIAN)MgMe].^27^ We suggest that the diphenylketeniminate derivative **5** is formed in a similar manner, in a process which is detrimentally competitive with the desired hydroboration reactivity under catalytic conditions (Table 1, entry 15).

### Kinetic studies

**Fig. 5:**
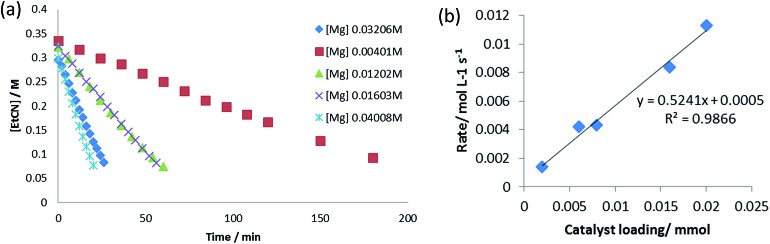
(a) *Pseudo*-zero order kinetics of propionitrile dihydroboration with varying [**V**]; (b) propionitrile dihydroboration reaction rate as a function of [**V**].

To further assess the mechanistic implications of these observations, a kinetic investigation of the hydroboration catalysis was undertaken. Propionitrile was selected as the representative alkyl nitrile substrate due to its provision of opportune reaction rates and clearly observed starting material and product resonances in the ^1^H NMR spectra. Initial reactions were carried out at 323 K and were monitored by ^1^H NMR spectroscopy to three half-lives (80% product conversion), using the standard catalytic reaction of 10 mol% of the precatalyst **V** in conjunction with a 1 : 2.1 ratio of propionitrile (0.40 M) to HBpin (0.82 M). While the reactions displayed apparent *pseudo*-zero order kinetic behaviour (Fig. 5a), a first order dependence on [**V**] was deduced through variation of the catalyst loading with maintenance of the same concentrations of propionitrile and HBpin (Fig. 5b). This latter observation is consistent with other magnesium- and calcium-catalysed reactions in which the alkaline earth centre is coordinated by a β-diketiminate ligand and,^12^ we suggest, is indicative of the involvement of an isolated magnesium centre during the rate determining process of the catalysis. Experiments under *pseudo*-first order conditions employing a large excess of propionitrile (8.0 M, 20 equivalents) signified a first order dependence on [HBpin] (Fig. S5–S8†). Although reactions performed with a twentyfold excess of HBpin and with variation of [EtCN] were also indicative of a first order dependence of the reaction rate with changing nitrile concentration (Fig. S9–S12†), further studies highlighted that the data acquired under these conditions may not be truly reflective of the stoichiometric catalytic process. A series of reactions performed with [HBpin] = 8.0 M and [EtCN] = 0.4 M, for example, undertaken with catalyst starting concentrations adjusted between 0.012 M and 0.068 M provided a series of *k*_obs_ which varied with the third power of [catalyst] (Fig. S13–S18†). In contrast further *pseudo*-first order experiments employing a large excess of EtCN provided the expected first order variation in rate with respect to changing [catalyst] (Fig. S19–S22†). We, thus, suggest that these latter experiments indicate a possible change in mechanism under *pseudo*-first order conditions in HBpin. Variation of both [EtCN] and [HBpin] under a concentration regime more representative of the 2 : 1 HBpin : EtCN ratio required for the dihydroboration (Fig. S23–S30†) also indicated a marked dependence on the reaction stoichiometry and maximum (saturating) substrate concentration limits reminiscent of enzymatic Michaelis–Menten kinetics.

We interpret the suppression of reaction rate at high substrate concentrations on the ability of both reagents to coordinate to the metal centre, either as a neutral nitrile donor or through the likely formation of borohydride species. We suggest that this deduction is further supported by the first order dependence of the reaction rate on [HBpin] in the presence of excess EtCN wherein increasing concentration will favour the displacement of the neutral nitrile from the magnesium coordination sphere allowing the onward formation of intermediate aldimidoborohydride species akin to compound **4** and resultant catalytic turnover. A comparison of the *pseudo*-zero order rate constants for hydroboration of propionitrile in which HBpin was replaced by DBpin provided a kinetic isotope effect (KIE, *k*_H_/*k*_D_) of 2.79. Although relatively small, this value would constitute a very large secondary effect and is significantly in excess of comparable results (*k*_H_/*k*_D_ = 1.62) reported by Hartwig *et al.* during studies of catecholborane metathesis at ruthenium(ii) alkyl centres, in which B–H bond breaking processes are integral to the progress of the reaction.^28^ We, thus, deduce that species analogous to compound **4** do not persist under catalytic conditions but are consumed by intramolecular hydride transfer from HBpin to the metal-bound aldimide fragment.

**Fig. 6:**
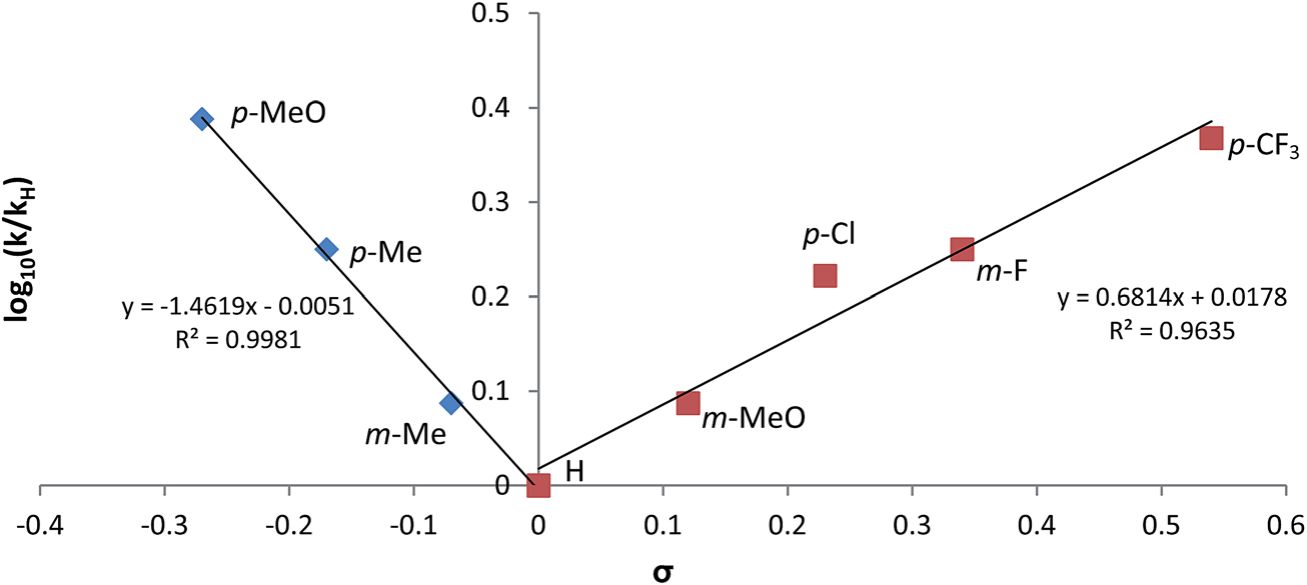
Hammett plot for dihydroboration of variously substituted aryl nitriles catalysed by **V**.

In contrast to the *pseudo*-zero order kinetics displayed during the hydroboration of propionitrile, experiments performed to interrogate the rates of reactions displayed by the array of aryl nitriles employed in the study (Table 1) evidenced significantly divergent characteristics. In all cases, reactions performed with the 1 : 2 nitrile/HBpin ratio required by the reaction stoichiometry conformed to unambiguous first order kinetic behaviour (Fig. S37†), the observed rate constants of which varied internally across the range of aryl substitution patterns employed. These data were employed to construct the chevron-shaped Hammett plot shown in Fig. 6, which clearly indicates a change in reaction mechanism at the transition from overall electron donating [denoted as Ar(EDG)CN] to electron withdrawing [denoted as Ar(EWG)CN] aryl nitrile substitution.^29^ Although the resultant *ρ* values [*ρ*(EDG) = –1.46; *ρ*(EWG) = +0.68] are relatively small and must be interpreted with caution,^30^ the change in sign is indicative of minor, yet mechanistically significant, adjustments to the redistribution of electronic charge during the traversal of the rate determining transition state(s).

This apparent change in mechanism was interrogated through kinetic studies of the hydroboration of nitrile substrates bearing representative electron donating (*p*-MeOC_6_H_4_CN) and electron withdrawing (*m*-MeOC_6_H_4_CN) aryl substitution. For the hydroboration of *p*-MeOC_6_H_4_CN a first order dependence on the concentration of [catalyst] (Fig. S41 and S43†) was again consistent with catalysis mediated by a single magnesium centre. A series of reactions conducted at 323 K with 10 mol% of **V** under *pseudo*-first order conditions employing 20 molar equivalents of the nitrile substrate, however, indicated a second order dependence on [HBpin] for *p*-MeOC_6_H_4_CN (Fig. S57†). An inverse dependence on [Ar(EDG)CN] again denoted nitrile inhibition as a key influence on the observed reaction rate (Fig. S50†). We suggest that the experimental reaction order in [HBpin] does not reflect the molecularity of the rate determining process. Rather, the second order dependence on [HBpin] is likely the result of two sequential reactions between an intermediate magnesium species and the borane substrate (*vide infra*).

Despite similar global first order rate behaviour, analogous kinetic experiments for the hydroboration of *m*-MeOC_6_H_4_CN evidenced dissimilar dependences on the concentrations of the reaction components. In contrast to the expectation provided by the kinetic analyses of the EtCN- and *p*-MeOC_6_H_4_CN-based reactions, these latter experiments highlighted a second order dependence on the precatalyst concentration [**V**] (Fig. S80†). Furthermore, whereas reactions performed with DBpin and *p*-MeOC_6_H_4_CN provided *k*_H_/*k*_D_ = 2.44 commensurate with that deduced for propionitrile hydroboration, analogous experiments performed with *m*-MeOC_6_H_4_CN did not provide any retardation or acceleration of the reaction rate and *k*_H_/*k*_D_ = 1.00. These latter data confirm that the HBpin substrate plays an influential role in the reduction of aryl nitriles with electron donating substitution patterns but has no influence on the turnover limiting process during the magnesium-catalysed hydroboration of more electron poor nitrile substrates.

In a similar manner to that noted for kinetic experiments performed with EtCN, the rates of dihydroboration reactions performed on both aryl nitriles studied in the presence of either a large excess of HBpin or the nitrile substrate were seen to display variable reaction orders in [catalyst] which were dependent on the concentration regime employed. Whilst we consider it imprudent to speculate on the origin of these adjustments, it is clear that the reactions again undergo a change in mechanism in the presence of a saturating concentration of one or the other substrate. With this proviso in place, however, it is clearly apparent that a change in the disposition of the nitrile substrate yields a decisive influence over the course of the catalysis.

Further insight into the nature of the mechanistic discontinuities was, thus, obtained from variable temperature kinetic studies of all three reaction types. The macro- and microscopic activation parameters for the hydroboration of propionitrile, *p*-MeOC_6_H_4_CN and *m*-MeOC_6_H_4_CN mediated by 10 mol% **V** were determined through Arrhenius and Eyring analyses for reactions performed at four temperatures with standard errors calculated using the least squares method for linear regression. The data presented in Table 5 again evidence considerable variability and are strongly reflective of the dissimilar turnover limiting and bond breaking and forming processes across the three substrate classes. Whilst the hydroboration of propionitrile is enthalpically disadvantaged, the catalysis benefits from a markedly less negative (effectively zero) activation entropy than that deduced for either aryl nitrile substrate. This observation, in conjunction with the significant KIE, further implicates a combination of borane and nitrile substrates which are pre-assembled at the catalytic magnesium centre prior to B–H bond rupture. We thus suggest that the structure of compound **4** (Fig. 3) provides a viable solid-state model for the assembled catalytic intermediate during the hydroboration of alkyl nitriles *prior* to an intramolecular B–N bond forming and B–H bond breaking process to form a magnesium borylamido species which is in turn rapidly consumed by facile Mg–N/B–H metathesis with a further equivalent of HBpin.

**Table 5:** Kinetic activation parameters for the hydroboration of EtCN, *p*-MeOC_6_H_4_CN and *m*-MeOC_6_H_4_CN with HBpin, mediated by 10 mol% **V**

Nitrile	*E*_a_ (kJ mol^–1^)	Δ*H*^≠^ (kJ mol^–1^)	Δ*S*^≠^ (J K^–1^ mol^–1^)
EtCN	106.5 (±3.0)	103.8 (±3.0)	–4.1 (±9.4)
*p*-MeOC_6_H_4_CN	53.1 (±5.8)	50.4 (±5.8)	–174.8 (±18.2)
*m*-MeOC_6_H_4_CN	46.2 (±3.1)	43.5 (±3.1)	–197.6 (±9.7)

In contrast, the Δ*H*^≠^ and Δ*S*^≠^ values for reactions encompassing both electron donating and electron withdrawing aryl substituents are notably influenced by entropic rather than enthalpic considerations. While the enthalpic contribution to the microscopic activation free energies for both aryl nitrile-based catalyses are *ca.* 50% of that deduced for EtCN, a significantly negative activation entropy of *ca.* –190 J K^–1^ mol^–1^ is, in both cases, indicative of the associative formation of activated complexes through the assembly of two or more reaction components and must be accounted for in any mechanistic rationale.

## Discussion

The kinetic data and the demarcation between electron donating and electron withdrawing aryl nitrile substitution illustrated by the Hammett analysis (Fig. 6) are suggestive of significant variability across the catalytic reactions. We suggest, however, that each reaction conforms to a common mechanism and that the differing kinetic profiles for the three substrate classes are indicative of a series of substrate-dependent pre-equilibria established during the multi-step catalytic reaction.

**Scheme 4:**
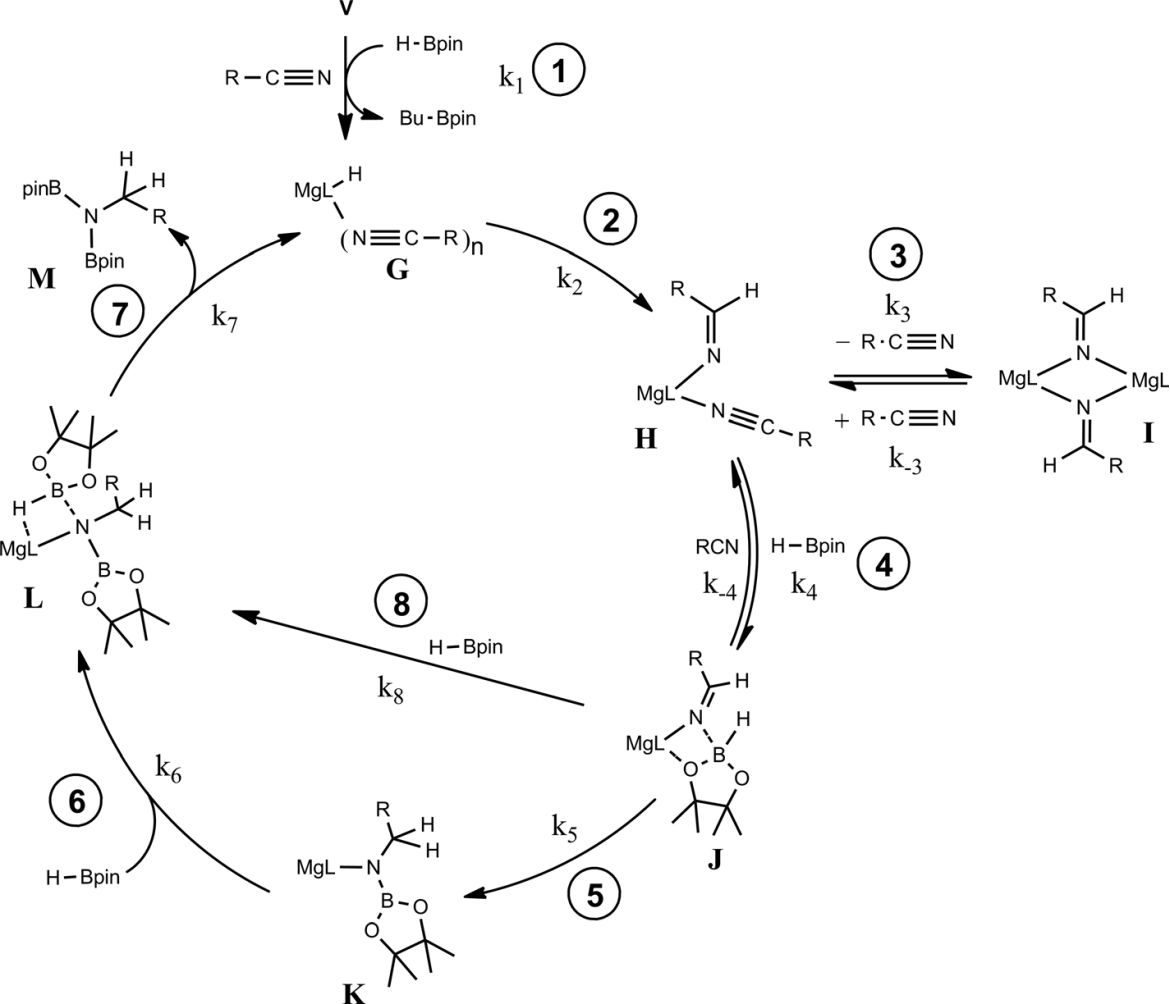


Scheme 4 provides a catalytic mechanism that, although common to each substrate, allows the discrimination of divergent turnover limiting behaviour for (a) alkyl nitriles (RCN), (b) aryl nitriles with appended electron donating groups [Ar(EDG)CN] and (c) aryl nitriles with electron withdrawing groups [Ar(EWG)CN]. Entrance to the catalytic manifold presents a common initiation step involving the transformation of the magnesium precatalyst (**V**) into a hydride intermediate (**G**) through metathesis of the magnesium–butyl bond with HBpin (designated as the circled reaction 1 in Scheme 4). Reaction of the nitrile substrate with magnesium hydride and the formation of aldimide derivatives (**H** and **I**) exemplified by the isolation of compounds **2** and **3** appears facile, irrespective of nitrile substrate identity (reaction 2). Whilst the activation of organonitriles toward different nucleophiles (*e.g.* water, alcohols, amines,^31^ mercaptans,^32^ phosphines,^33^ oximes^34^) by coordination to transition metal centres is well precedented and has been rationalised by an increase of the effective positive charge induced at the nitrile sp-carbon centre,^35^ this process appears to have little impact on the overall facility for catalytic turnover. We, thus, propose that the rate determining processes of the reactions diverge under the influence of a series of pre-equilibria within the catalytic manifold, which are themselves dictated by modulating nitrile basicity and magnesium aldimide stability. Although only minor variations in experimental and calculated gas-phase basicities and proton affinities have been reported across a wide variety of organic nitriles,^36^ even slight changes in basicity induced by the relative stabilizing influence of the *N*-alkyl or *N*-aryl residue will affect the access of HBpin to magnesium and the consequent formation of borate species akin to compound **4** necessary for successful catalytic turnover. From this perspective, the variation in kinetic behaviour across the three substrate classes (a)–(c) may be discriminated.

### Case (a): alkyl nitriles (RCN)

**Scheme 5:**
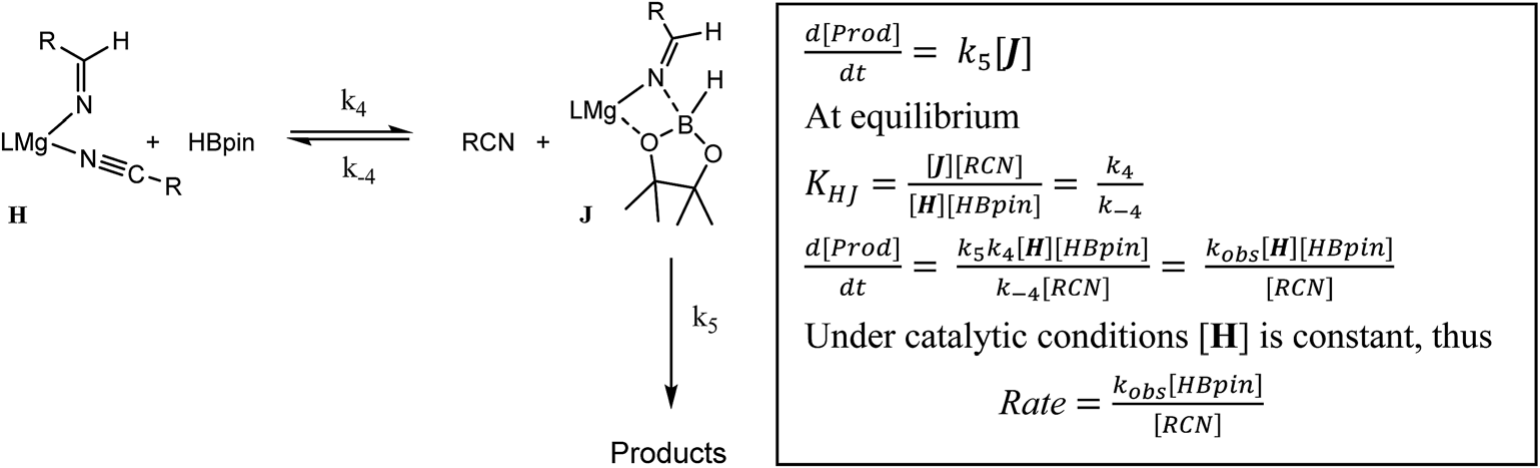


The more basic character of alkyl nitriles ensures that the monomer/dimer equilibrium depicted as reaction 3 in Scheme 4 favours the monomeric species, **H** (*i.e. k*_–3_ ≫ *k*_3_). The assembly of aldimidohydridoborate anions (**J**) such as that confirmed by the crystallographic characterisation of compound **4** requires the displacement of pre-coordinated nitrile by the HBpin substrate (reaction 4 depicted in Scheme 4), an equilibration process that is reflected in the inhibition of catalysis at higher propionitrile concentrations. Once formed, the facility of subsequent imine reduction by intramolecular boron-to-carbon hydride transfer to form the borylamide intermediate **K** will be dictated by the relative stability of the aldimide fragment. Subsequent Mg–N metathesis with a second equivalent of HBpin will then provide the ultimate bis(boryl)amine product (**M**) *via* the assembly of a further borate intermediate (**L**) (reactions 6 and 7, Scheme 4). For alkyl nitrile substrates we suggest that this process will be relatively facile under catalytic conditions with *k*_5_ > *k*_–4_ (Scheme 5) yielding the negligible entropic component (Δ*S*^≠^ = –4.1 (±9.4) J K^–1^ mol^–1^) of the free energy of activation for the alkyl nitrile hydroboration catalysis.

The catalytic hydroboration of alkyl nitriles is consequently determined by the pre-equilibration of **H** to **J** and its subsequent consumption through B–H transfer to the coordinated aldimide fragment. The observed rate of catalysis for alkyl nitrile hydroboration is, thus, dictated by not only the ability of HBpin to replace nitrile in the magnesium coordination sphere but also the consequent ease of intramolecular C=N hydride reduction.

### Case (b): aryl nitriles with appended electron donating groups (Ar(EDG)CN)

**Scheme 6:**
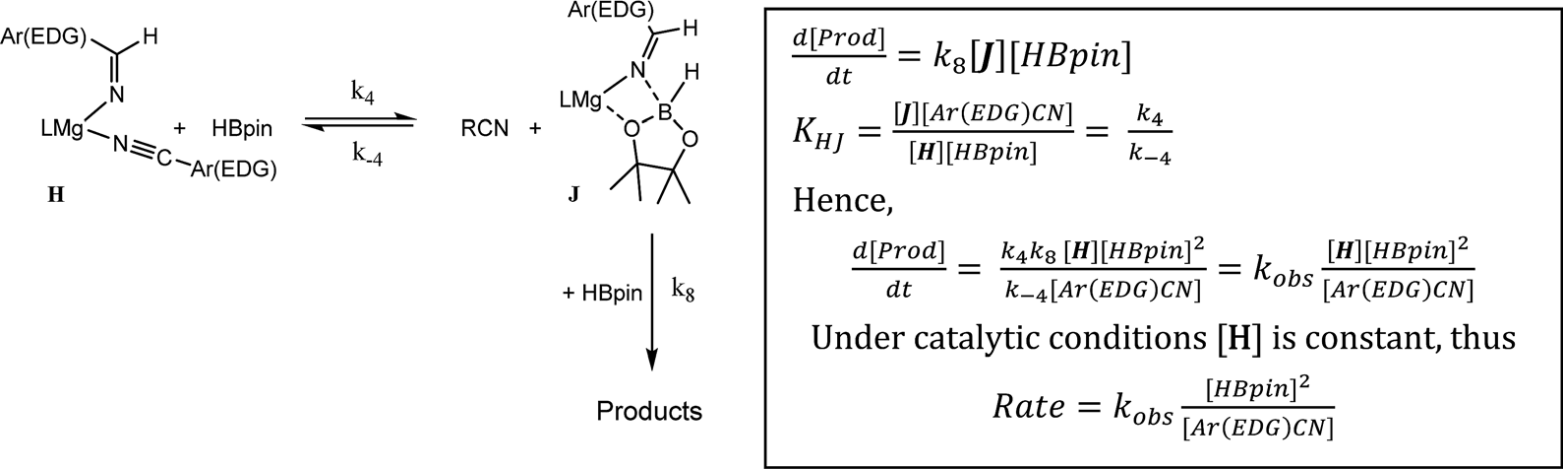


As highlighted by the isolation of compounds **2** and **3**, magnesium aldimide derivatives bearing *N*-aryl substitution will benefit from considerably enhanced conjugative stability in comparison to those bearing alkyl residues of comparable steric demands. We suggest, therefore, that the kinetic profile observed for the hydroboration of *p*-MeOC_6_H_4_CN reflects the resistance to intramolecular hydride transfer of aldimidoborate species analogous to compound **4** (species **J** in Scheme 4). Although the ease of formation of such species is again dictated by the equilibrium shown as reaction 4 in Scheme 4, the conjugative stability of the resultant aldimidoborate toward intramolecular C=N reduction suggests that the sequence of reactions 5–7 in Scheme 4 is inoperable for these intermediates. In such cases, we suggest that hydride transfer and consequent catalytic turnover occurs through reaction of species analogous to **J** with a further molecule of HBpin (shown as reaction 8 in Scheme 4). The KIE (2.44) associated with this process is comparable to that observed for alkyl nitrile hydroboration, which leads us to deduce that this most likely occurs as a sequence of elementary processes rather than an alternative pathway involving simultaneous aldimide reduction and Mg–N/B–H metathesis. The notably negative activation entropy deduced for this reaction (Δ*S*^≠^ = –174.8 (±18.2) J K^–1^ mol^–1^) is also congruent with pre-equilibration of Ar(EDG)CN and HBpin and the onward reaction with a second molecule of HBpin. Consideration of the entire process illustrated by Scheme 6 predicts a rate law which correctly encapsulates the experimentally determined second order dependence on [HBpin] while also accounting for the inhibitory effects of increasing nitrile concentration and the first order reliance on changing precatalyst concentration.

### Case (c): aryl nitriles with appended electron withdrawing groups (Ar(EWG)CN)

**Scheme 7:**
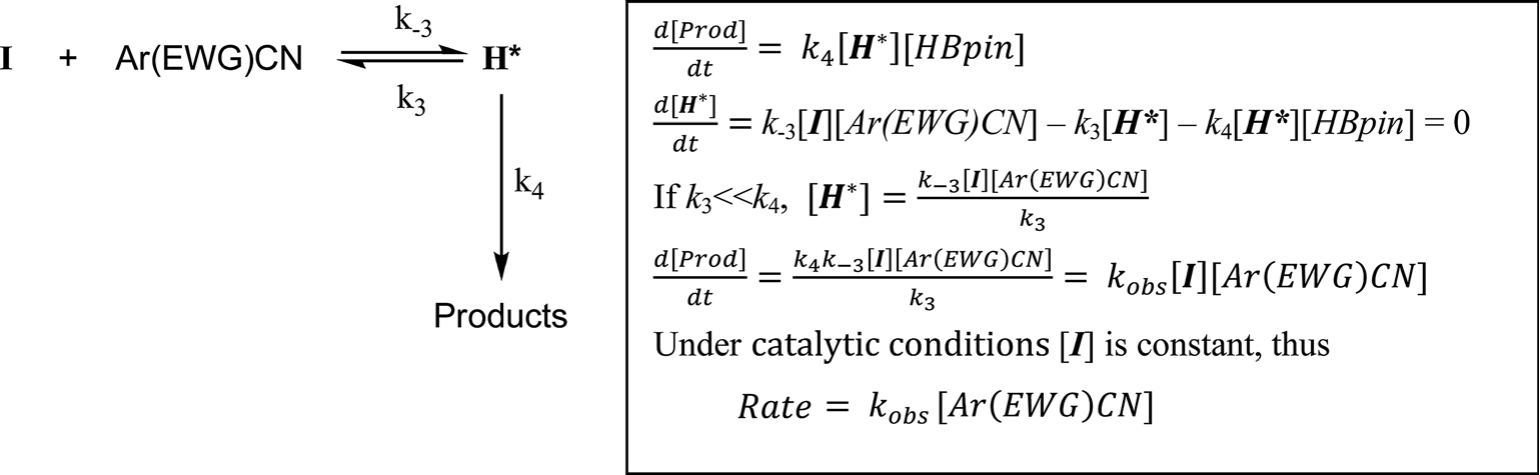


For 3-methoxybenzonitrile, Mg–H insertion will provide a dimeric aldimide (**I**) with a solution structure similar to that depicted for **2** in Fig. 2a. The apparent independence of the reaction rate on [HBpin] and the absence of a KIE resulting from its replacement in the reaction with DBpin indicates that HBpin is uninvolved in the rupture of this dimeric aldimide. The pre-equilibrium shown in Scheme 7 is, thus, entirely dependent on the ability of the weakly basic nitrile to coordinate to magnesium with rupture of the dimeric resting state to form an intermediate denoted as H* (*vide infra*). Use of the steady state approximation allows the derivation of a rate law that is dependent on [**I**] and may be identified as the rate determining process in a hydroboration catalysis in which *k*_3_ ≫ *k*_–3_ ≪ *k*_4_.

**Scheme 8:**
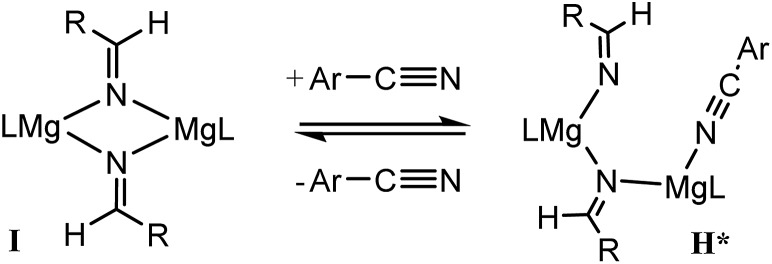


We suggest that this process, therefore, occurs with only partial rupture of the dimeric unit as illustrated in Scheme 8, whereupon subsequent reaction with HBpin can only take place at the terminal aldimide to magnesium bond of the unsymmetrical dimeric intermediate (H*). Under this regime the role of the nitrile substrate is encapsulated by the experimentally deduced second order dependence on initial precatalyst concentration, [**V**], and is simply reflective of the involvement of two magnesium centres in the formation of H*.

## Conclusion

In conclusion, the β-diketiminato magnesium species **V** has been demonstrated as an active precatalyst for the HBpin-derived hydroboration of a range of alkyl and aryl nitriles to form bis(boryl)amines. Catalysis proceeds under mild conditions, with reasonable catalyst loadings and is proposed to occur through a sequence of magnesium-mediated B–H insertion and metathesis steps that are crucially dependent on a variety of pre-equilibration steps that are dictated by minor variations in substrate basicity and the stability of mono- and dimeric intermediates. These observations indicate that, somewhat counter to historical prejudice, there is likely to be considerable variation across even superficially identical reactions when catalysed by alkaline earth reagents.

## Supplementary Material

Supplementary information

Crystal structure data
