# Supplementary material for: Magnesium-catalysed nitrile hydroboration
Source: Chem Sci. 2015 Oct 20;7(1):628–41. doi: 10.1039/c5sc03114a (PMC5952893; doi:10.1039/c5sc03114a)
Supplement: Supplementary file 1 [file SC-007-C5SC03114A-s001.pdf]

Electronic Supplementary Material (ESI) for Chemical Science.
This journal is © The Royal Society of Chemistry 2015

Supporting Experimental Information for

Magnesium-Catalysed Nitrile Hydroboration

Catherine Weetman, Mathew D. Anker, Merle Arrowsmith, Michael S. Hill,* Gabriele Kociok-Köhn, David J. Liptrot and Mary F. Mahon

Department of Chemistry, University of Bath, Claverton Down, Bath, BA2 7AY, UK

Experimental Data

General Experimental Procedures

All manipulations were carried out using standard Schlenk line and glovebox techniques under an inert atmosphere of argon. NMR experiments were conducted in Youngs tap NMR tubes made up and sealed in a Glovebox. NMR spectra were collected on a Bruker AV300 spectrometer operating at 300.2 MHz (^{1}H), 75.5 MHz (^{13}C), 96.3 MHz (^{11}B). The spectra were referenced relative to residual solvent resonances or an external BF_3.OEt_2 standard (^{11}B). Solvents (Toluene, THF, hexane) were dried by passage through a commercially available (Innovative Technologies) solvent purification system, under nitrogen and stored in ampoules over molecular sieves. C_6D_6 and d_8-toluene were purchased from Fluorochem Ltd. and dried over molten potassium before distilling under nitrogen and storing over molecular sieves. Di-*n*-butylmagnesium (1.0 M solution in n-heptane) and pinacolborane were purchased from Sigma-Aldrich Ltd. and used without further purification. [HC{(Me)CN(2,6-$^{i}Pr_2C_6H_3$)}$_2$Mg*n*Bu] was synthesised by a literature procedure.[1]

Stoichiometric Reactions

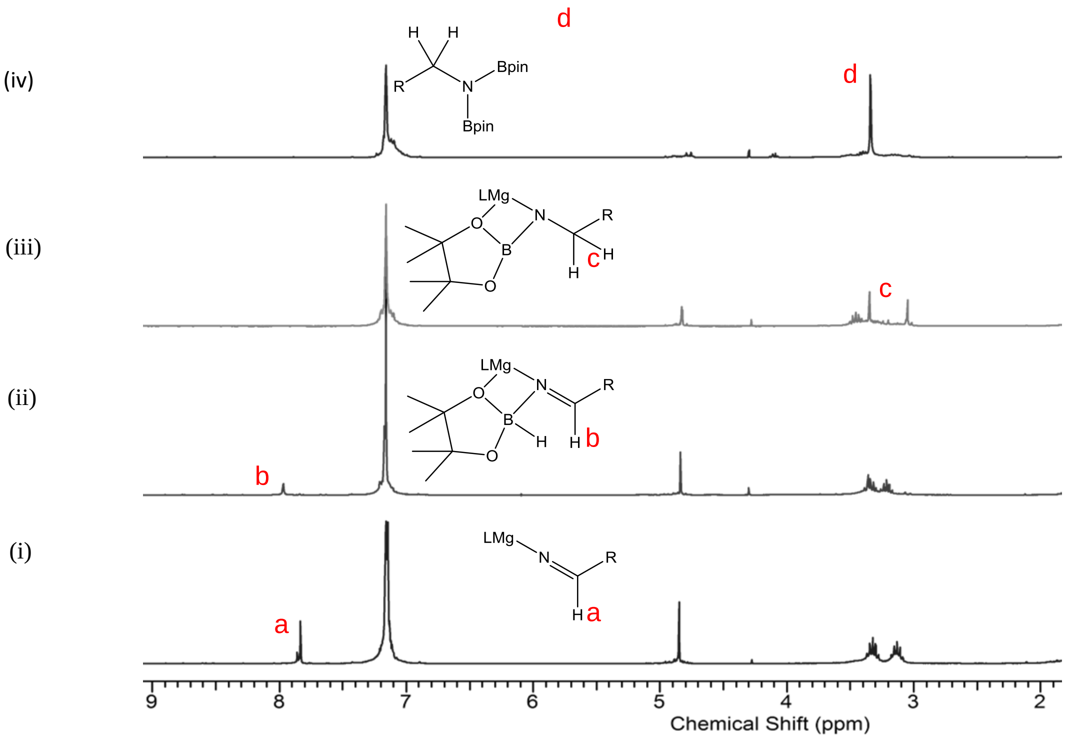

Scheme S1: Stacked ^{1}H NMR spectra in C_6D_6 recorded during the stoichiometric reduction of *t*-BuCN with HBpin. (i) Magnesium aldimide formation after addition of HBpin and *t*-BuCN to **V**; (ii) Magnesium aldimidoborate formation on addition of a further equivalent of HBpin; (iii) Intramolecular hydride transfer with formation of magnesium borylamide; (iv) Bis(boryl)amine formation after addition of a further equivalent of HBpin.

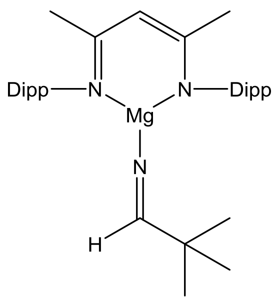

NMR Scale: LMgBu (**V**) (0.04 mmol, 20 mg) was dissolved in 0.5 ml of C_6D_6 along with HBpin (0.04 mmol, 5.8 μL). This was left at room temperature for 5 minutes to form LMgH in situ before adding ^{t}BuCN (0.04 mmol, 4.4 μL). This was heated at 60 ^{o}C overnight to yield the insertion product, LMgNCH^{t}Bu. ^{1}H NMR (C_6D_6, 300 MHz): 7.83 (1H, s, N=C*H*), 7.21 – 7.11 (6H, m, Ar-*H*), 4.85 (1H, s, NC(CH_3)C*H*), 3.32 (2H, sept, J_{HH} = 6 Hz, C*H*(CH_3)$_2$), 3.13 (2H, sept, J_{HH} = 6 Hz, C*H*(CH_3)$_2$), 1.66 (6H, s, NC(CH_3)CH), 1.43 (6H, d, J_{HH} = 9 Hz, CH(CH_3)$_2$), 1.23 (6H, d, J_{HH} = 9 Hz, CH(CH_3)$_2$), 1.20 (6H, d, J_{HH} = 9 Hz, CH(CH_3)$_2$), 1.12 (6H, d, J_{HH} = 9 Hz, CH(CH_3)$_2$), 1.03 (9H, s, C(CH_3)$_3$. ^{13}C{^{1}H} NMR (C_6D_6, 75 MHz): 174.5 (N=*C*H), 170.5 (N*C*(CH_3)), 147.0 (*ipso*-*C*-Ar), 142.8 (*ortho*-*C*-Ar), 142.5 (*ortho*-*C*-Ar), 126.2 (*para*-*C*-Ar), 124.6 (*meta*-*C*-Ar), 96.1 (NC(CH_3)*C*H), 37.5 (N=CH*C*(CH_3)$_3$), 29.2 (*C*H(CH_3)$_2$), 27.2, 27.1, 26.1, 25.7, 25.0, 24.8.

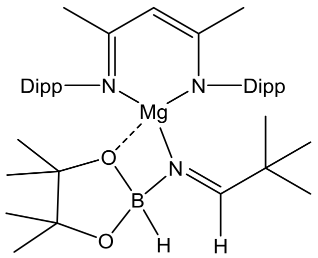

Compound 4: NMR Scale: To the previous solution additional HBpin (0.04 mmol, 5.8 μL) was added and left overnight at room temperature to yield the borate intermediate. ^{1}H NMR (C_6D_6, 300 MHz): 7.97 (1H, s, N=C*H*), 7.21 – 7.10 (6H, m, Ar-*H*), 4.84 (1H, s, NC(CH_3)C*H*), 3.34 (2H, sept, J_{HH} = 6 Hz, C*H*(CH_3)$_2$, 3.21 (2H, sept, J_{HH} = 6 Hz, C*H*(CH_3)$_2$), 1.63 (6H, s, NC(CH_3)CH), 1.40 (6H, d, J_{HH} = 9 Hz, CH(CH_3)$_2$), 1.37 (6H, d, J_{HH} = 9 Hz, CH(CH_3)$_2$), 1.23 (6H, d, J_{HH} = 9 Hz, CH(CH_3)$_2$), 1.21 (6H, d, J_{HH} = 9 Hz, CH(CH_3)$_2$), 1.07 (12H, s, OC(CH_3)$_2$), 1.00 (9H, s, C(CH_3)$_3$). ^{13}C{^{1}H} NMR (C_6D_6, 75 MHz): 178.4 (N=*C*H), 170.5 (N*C*(CH_3)), 145.7 (*ipso*-*C*-Ar), 143.3 (*ortho*-*C*-Ar), 142.6 (*ortho*-*C*-Ar), 126.1 (*para*-*C*-Ar), 124.7 (*meta*-*C*-Ar), 124.4 (*meta*-*C*-Ar), 96.0 (NC(CH_3)*C*H), 83.0 (O*C*(CH_3)$_2$), 82.6 (O*C*(CH_3)$_2$), 37.8 (N=CH*C*(CH_3)$_3$), 29.17 (*C*H(CH_3)$_2$), 28.4 (*C*H(CH_3)$_2$), 27.5, 27.2, 26.1, 25.9, 25.3 (OC(*C*H_3)$_2$), 25.1. ^{11}B NMR (C_6D_6, 96 MHz): 8.5 (d, J_{HB} = 105.6 Hz, N*B*H).

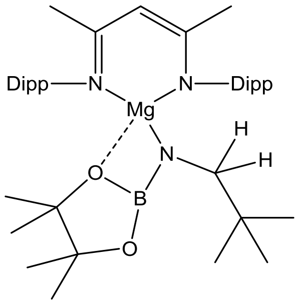

NMR Scale: Previous NMR sample was allowed to stand at room temperature for 48 hrs. ^{1}H NMR (C_6D_6, 300 MHz): 7.21 – 7.10 (6H, m, Ar-*H*), 4.83 (1H, s, NC(CH$_3$)C*H*), 3.46 (2H, sept, J_{HH} = 6 Hz, C*H*(CH$_3$)$_2$), 3.35 (2H, s, NC*H*$_2$), 3.31 (2H, sept, J_{HH} = 6 Hz, C*H*(CH$_3$)$_2$), 1.64 (6H, s, NC(C*H*$_3$)CH), 1.39 (12H, d, J_{HH} = 6 Hz, CH(C*H*$_3$)$_2$), 1.26 (12H, d, J_{HH} = 6 Hz, CH(C*H*$_3$)$_2$), 1.06 (24H, s, OC(C*H*$_3$)$_2$). ^{13}C{^{1}H} NMR (C_6D_6, 75 MHz): 170.6 (N*C*(CH$_3$)CH), 145.8 (*ipso-C*-Ar), 143.1 (*ortho-C*-Ar), 125.8 (*para-C*-Ar), 124.4 (*meta-C*-Ar), 95.8 (NC(CH$_3$)*C*H), 83.1 (O*C*(CH$_3$)$_2$), 82.6 (O*C*(CH$_3$)$_2$), 58.5 (N*C*H$_2$), 34.3 (NCH$_2$*C*(CH$_3$)$_3$), 28.6, 28.4, 27.2, 26.2, 25.9, 25.3, 25.1, 14.7 (C(*C*H$_3$)$_3$). ^{11}B NMR (C_6D_6, 96 MHz): 7.05 (d, J_{HB} = 105.6 Hz, N*B*H).

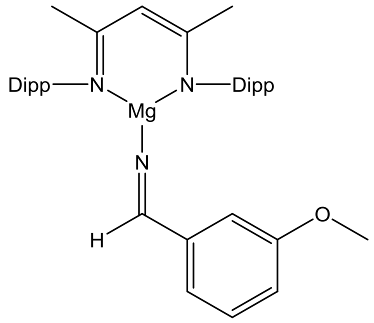

Compound 2: NMR Scale: LMgBu **(V)** (0.06 mmol, 30 mg) was dissolved in 0.5 ml of C_6D_6 along with HBpin (0.06 mmol, 8.4 μL). This was left at room temperature for 5 minutes to form LMgH in situ before adding (3-MeO)PhCN (0.06 mmol, 7.3 μL). This was heated at 60 ^{o}C overnight to yield the insertion product, LMgNCHPh(3-MeO). ^{1}H NMR (C_6D_6, 300 MHz): 8.63 (1H, s, NC*H*), 7.56 – 6.80 (10H, m, Ar-*H*), 4.89 (1H, s, NC(CH$_3$)C*H*), 3.42 (3H, s, OC*H*$_3$), 3.29 (4H, m, C*H*(CH$_3$)$_2$), 1.68 (6H, s, NC(C*H*$_3$)CH), 1.42 (6H, d, J_{HH} = 6Hz, CH(C*H*$_3$)$_2$), 1.24 (6H, d, J_{HH} = 6Hz, CH(C*H*$_3$)$_2$), 0.97 (6H, d, J_{HH} = 6Hz, CH(C*H*$_3$)$_2$), 0.90 (6H, d, J_{HH} = 6Hz, CH(C*H*$_3$)$_2$). ^{13}C{^{1}H} NMR (C_6D_6, 75 MHz): 172.5 (N=*C*H), 170.4 (N*C*(CH$_3$)CH), 165.0, 160.9, 147.7, 145.2, 144.1, 142.9, 139.4, 130.3, 126.2, 124.8, 95.2 (NC(CH$_3$)*C*H), 55.6 (O*C*H$_3$), 32.3 (*C*H(CH$_3$)$_2$), 29.6, 28.7, 28.2, 27.2, 24.7, 23.4.

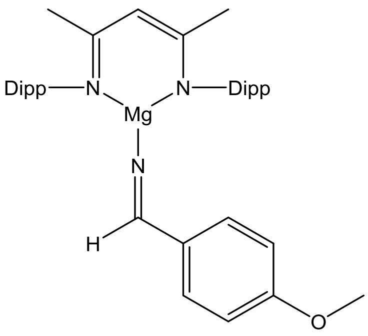

Compound 3: NMR Scale: LMgBu (**V**) (0.06 mmol, 30 mg) was dissolved in 0.5 ml of C_6D_6 along with HBpin (0.06 mmol, 8.4 μL). This was left at room temperature for 5 minutes to form LMgH in situ before adding (4-MeO)PhCN (0.06 mmol, 8.0 mg). This was heated at 60 °C overnight to yield the insertion product, LMgNCHPh(4-MeO). ^{1}H NMR (C_6D_6, 300 MHz): 8.58 (1H, s, NC*H*), 7.80 – 6.81 (10H, m, Ar-*H*), 4.87 (1H, s, NC(CH_3)C*H*), , 3.66 (4H, m, C*H*(CH_3)$_2$), 2.99 (3H, s, OC*H*$_3$) 1.73 (6H, s, NC(C*H*$_3$)CH), 1.47 (6H, d, J_{HH} = 6Hz, CH(C*H*$_3$)$_2$), 1.32 (6H, d, J_{HH} = 6Hz, CH(C*H*$_3$)$_2$), 0.91 (6H, d, J_{HH} = 6Hz, CH(C*H*$_3$)$_2$), 0.89 (6H, d, J_{HH} = 6Hz, CH(C*H*$_3$)$_2$). ^{13}C{^{1}H} NMR (C_6D_6, 75 MHz): 171.7 (N=*C*H), 169.6 (N*C*(CH_3)CH), 162.3, 147.7, 146.7, 144.1, 142.9, 135.0, 126.2, 124.2, 94.7 (NC(CH_3)*C*H), 55.3 (O*C*H$_3$), 32.3 (*C*H(CH_3)$_2$), 29.8, 28.8, 27.2, 26.1, 25.8, 24.9, 24.6, 23.4.

Catalytic reactions

NMR scale: 10 mg (0.02 mmol, ie. 10 mol%) of LMgBu was dissolved in 0.5 ml of C_6D_6, 60.9 µL (0.42 mmol) of pinacolborane was then added followed by 0.2 mmol of nitrile. This mixture was then transferred to a sealed Youngs tap NMR tube and the reaction was kept in an oil bath at 60 °C. These were regularly monitored by ^{1}H and ^{11}B NMR spectroscopy until complete conversion was observed.

Scale up: In a Schlenk flask 50mg (0.1 mmol, ie. 10 mol%) of LMgBu was dissolved in 5ml of toluene, 304.7 µL (2.1 mmol) of pinacolborane was then added followed by 1 mmol of nitrile. This mixture was then transferred to an oil bath at 60°C, for the observed NMR reaction time. Toluene was then removed *in vacuo* and the remaining solid was redissolved in the minimum volume of hexane and left to crystallize in the freezer overnight.

***N-{B(OCMe$_2$)$_2$}* -propan-1-amine**

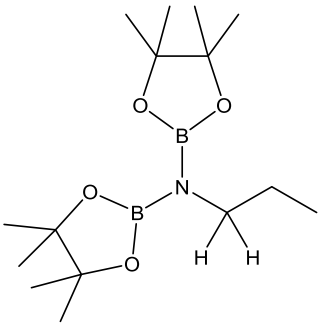

NMR scale: 14.3 µL of propionitrile. ^{1}H NMR (C_6D_6, 300 MHz): 3.42 (2H, t, J_{HH} = 6 Hz, NC*H$_2$*), 1.75 (2H, m, J_{HH} = 6 Hz, C*H$_2$*CH$_3$), 1.07 (24H, s, OCC*H$_3$*), 0.96 (3H, J_{HH} = 9 Hz, CH$_2$C*H$_3$*). ^{13}C{^{1}H} NMR (75.5 MHz, C_6D_6, 298 K): 82.6 (O*C*(CH$_3$)$_2$), 46.5 (N*C*H$_2$), 27.2 (CH$_2$*C*H$_2$CH$_3$), 25.1 (OC(*C*H$_3$)$_2$), 11.9 (CH$_2$*C*H$_3$). ^{11}B NMR (96.3 MHz, C_6D_6, 298 K): 29.5 N*B*. **Scale up:** 71.3 µL of propionitrile, 60 °C for 1 hr. Isolated as yellow crystals (228 mg, 70% yield). Elemental analysis: calcd. (found) for $C_{15}H_{31}B_2NO_4$: C 57.92 (57.68); H 10.05 (10.14); N 4.50 (4.40).

***N-{B(OCMe$_2$)$_2$}* - 2-methylpropan-1-amine**

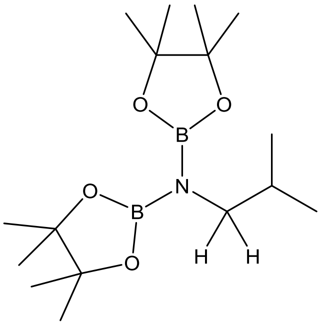

NMR scale: 18.0 µL of isobutyronitrile, 60 °C for 1 hr. ^{1}H NMR (300 MHz, C_6D_6, 298 K): 3.28 (2H, d, J_{HH} = 6 Hz, NC*H$_2$*), 2.05 (1H, m, J_{HH} = 6 Hz, C*H*(CH$_3$)$_2$), 1.07 (24H, s, OC(C*H$_3$*)$_2$), 1.01 (6H, d, J_{HH} = 6 Hz, CH(C*H$_3$*)$_2$). ^{13}C{^{1}H} NMR (75.5 MHz, C_6D_6, 298 K): 82.6 (O*C*(CH$_3$)$_2$), 52.1 (N*C*H$_2$), 31.6 (*C*H(CH$_3$)$_2$), 25.1 (OC(*C*H$_3$)$_2$), 20.7 (CH(*C*H$_3$)$_2$). ^{11}B NMR (96.3 MHz, C_6D_6, 298 K): 29.6 N*B*. **Scale up:** 89.8 µL of isobutyronitrile. Isolated as a yellow oil (313 mg, 96% yield). An accurate microanalysis could not be obtained for this compound.

***N-{B(OCMe₂)₂}* - 2,2-dimethylpropan-1-amine**

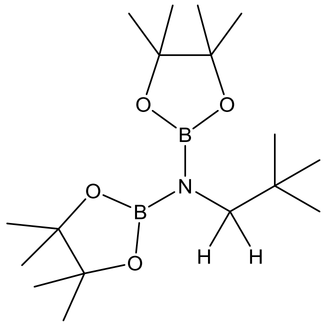

NMR scale: 22.1 μL trimethylacetonitrile, 60 °C for 5.5 hr. ^{1}H NMR (300 MHz, C_6D_6, 298 K): 3.30 (2H, s, NCH_2), 1.08 (24H, s, OC(CH_3)$_2$), 1.03 (9H, s, C(CH_3)$_3$). ^{13}C{^{1}H} NMR (75.5 MHz, C_6D_6, 298 K): 82.6 (O*C*(CH$_3$)$_2$), 55.3 (N*C*H$_2$), 34.0 (*C*(CH$_3$)$_3$), 28.4 (C(*C*H$_3$)$_3$), 25.1 (OC(*C*H$_3$)$_2$)). ^{11}B NMR (96.3MHz, C_6D_6, 298 K) δB(ppm): 29.5 N*B*. **Scale up:** 110.5 μL of trimethylacetonitrile. Isolated as colorless crystals (181 mg, 54% yield). Elemental analysis: calcd. (found) for $C_{17}H_{35}B_2NO_4$: C 60.22 (60.11); H 10.40 (10.55); N 4.13 (3.95).

***N-{B(OCMe₂)₂}* – cyclohexylmethanamine**

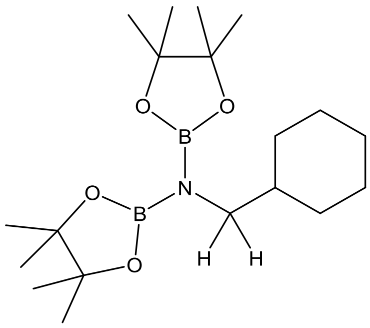

NMR scale: 23.8 μL cyclohexanitrile, 60 °C for 1 hr. ^{1}H NMR (300 MHz, C_6D_6, 298 K): 3.31 (2H, d, J_{HH} = 6 Hz, NCH_2), 1.92 (2H, m, NCH$_2$C*H*) 1.70 – 1.20 (10H, m, Cy-*H*), 1.07 (24H, s, OCCH_3). ^{13}C{^{1}H} NMR (75.5MHz, C_6D_6, 298 K): 82.6 (O*C*(CH$_3$)$_2$), 50.8 (N*C*H$_2$), 41.3 (NCH$_2$*C*H), 31.5 (Cy-*C*), 27.6 (Cy-*C*), 27.0 (Cy-*C*), 25.1 (OC(*C*H$_3$)$_2$). ^{11}B NMR (96.3 MHz, C_6D_6, 298 K): 29.7 N*B*. **Scale up:** 118.8 μL of cyclohexanitrile, 60 °C for 1 hr. Isolated as colorless crystals (265 mg, 75% yield). An accurate microanalysis could not be obtained for this compound.

***N-{B(OCMe$_2$)$_2$}* – phenylmethanamine**

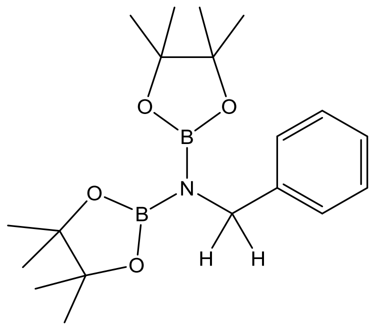

NMR scale: 19.5 µL benzonitrile, 60 °C for 12 hr. ^{1}H NMR (300 MHz, C_6D_6, 298 K): 7.57 (2H, m, *o*-H), 7.25 (2H, m, *m-H*), 7.11 (1H, m, *p-H*), 4.60 (2H, s, NC*H*$_2$), 1.02 (24H, s, OC(C*H*$_3$)$_2$). ^{13}C{^{1}H} NMR (75.5 MHz, C_6D_6, 298 K): 144.1 (*o-C*), 128.4 (*p*-C), 127.0 (*m-C*), 82.9 O*C*(CH$_3$)$_2$, 48.2 (N*C*H$_2$), 25.1 (OC(*C*H$_3$)$_2$). ^{11}B NMR (96.3 MHz, C_6D_6, 298 K): 29.5 N*B*. **Scale up:** 103.12 µL of Benzonitrile, 60 °C for 15 hrs. Isolated as colorless crystals (202 mg, 56% yield). Elemental analysis: calcd. (found) for $C_{19}H_{31}B_2NO_4$: C 63.55 (63.38); H 8.70 (8.82); N 3.90 (4.00).

***N-{B(OCMe$_2$)$_2$}* - *o*-tolylmethanamine**

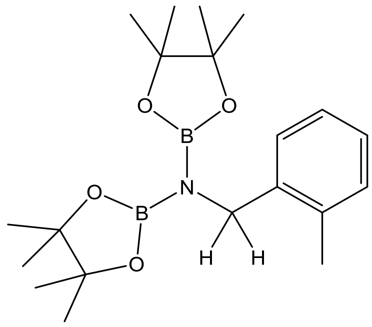

NMR scale: 23.7 µL o-tolunitrile, 60 °C for 15 hr. ^{1}H NMR (300 MHz, C_6D_6, 298 K): 7.64 (1H, d, J_{HH} = 6 Hz, *o-H*), 7.24 (1H, m, *p*-H), 7.08 (1H, m, *m-H*), 7.00 (1H, m, *m-H*), 4.60 (2H, s, NC*H*$_2$), 2.12 (3H, s, *o*-C*H*$_3$), 1.03 (24H, s, OC(C*H*$_3$)$_2$). ^{13}C{^{1}H} NMR (75.5 MHz, C_6D_6, 298 K): 141.5 (*o-C*), 135.7 (*o-C*CH$_3$), 130.5 (*p-C*), 126.3 (*m-C*), 125.9 (*m-C*HC(CH$_3$), 82.9 (O*C*(CH$_3$)$_2$), 45.8 (N*C*H$_2$), 25.0 (OC(*C*H$_3$)$_2$), 19.4 (*o-C*H$_3$). ^{11}B NMR (96.3 MHz, C_6D_6, 298 K): 29.8 N*B*. **Scale up:** 118.5 µL of *o*-tolunitrile, 60 °C for 15 hrs. Isolated as colorless crystals (275 mg, 73% yield). Elemental Analysis for $C_{20}H_{33}B_2NO_4$: Calculated (found): C 64.38 (64.45); H 8.92 (8.85); N 3.75 (3.63).

***N*-{B(OCMe$_2$)$_2$} - *m*-tolylmethanamine**

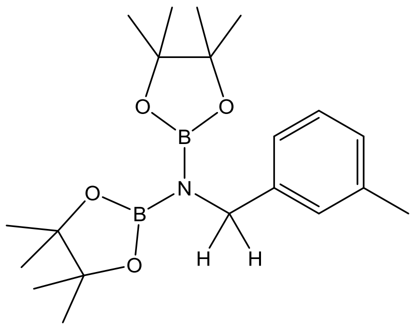

NMR scale: 22.0 µL m-tolunitrile, 60 °C for 15 hr. ^{1}H NMR (300 MHz, C_6D_6, 298 K): 7.43 (1H, d, J_{HH} = 6 Hz, *o-H*), 7.38 (1H, s, *o-H*), 7.20 (1H, m, *p-H*), 6.95 (1H, m, *m-H*), 4.60 (2H, s, NC*H*$_2$), 2.19 (3H, s, *m*-C*H*$_3$), 1.04 (24H, s, OC(C*H*$_3$)$_2$). ^{13}C{^{1}H} NMR (75.5 MHz, C_6D_6, 298 K): 144.0 (*o-C*), 137.8 (*o*-*C*HC(CH$_3$)), 129.3 (*p-C*), 128.7 (*i*-C) 127.7 (*m*-*C*CH$_3$), 125.4 (*m-C*), 82.9 (O*C*(CH$_3$)$_2$), 48.2 (N*C*H$_2$), 25.1 (OC(*C*H$_3$)$_2$), 21.9 (Ar*C*H$_3$). ^{11}B NMR (96.3 MHz, C_6D_6, 298 K): 29.9 N*B*. **Scale up:** 120.0 µL of *m*-tolunitrile, 60 °C for 15 hrs. Isolated colorless crystals (305 mg, 81% yield). An accurate microanalysis could not be obtained for this compound.

***N*-{B(OCMe$_2$)$_2$} - *3*-(fluoro)phenylmethanamine**

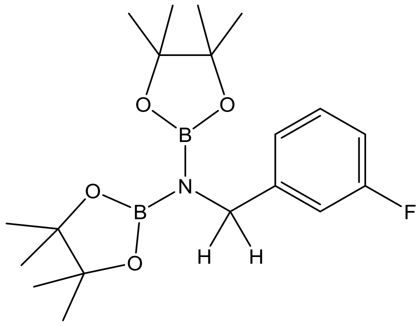

NMR scale: 21.4 µL 3-(fluoro)benzonitrile, 60 °C for 14 hr. ^{1}H NMR (300 MHz, C_6D_6, 298 K): 7.40 (1H, m, *o*-C*H*), 7.25 (1H, d, J_{HH} = 7.3 Hz, *o*-C*H*), 7.00 (2H, m, *m*-C*H*, *p*-C*H*), 4.51 (2H, s, NC*H*$_2$), 1.02 (24H, s, OC(C*H*$_3$)$_2$). ^{13}C{^{1}H} NMR (75.5 MHz, C_6D_6, 298 K): 130.2 (*o-C*), 128.7 (*o-C*), 128.5 (*p-C*), 128.5 (*m-C*), 128.3 (*m-C*), 83.1 (O*C*(CH$_3$)$_2$), 47.8 (N*C*H$_2$), 25.0 (OC(*C*H$_3$)$_2$). ^{11}B NMR (96.3 MHz, C_6D_6, 298 K): 29.5 N*B*. **Scale up:** 106.9 µL of 3-(fluoro)benzonitrile, 60 °C for 14 hrs. Isolated pale yellow crystals (215 mg, 59% yield). Elemental analysis: calcd. (found) for $C_{19}H_{30}B_2FNO_4$: C 60.52 (60.55); H 8.02 (7.93); N 3.71 (3.85).

N-{B(OCMe$_2$)$_2$} - 3-**(methoxy)phenylmethanamine**

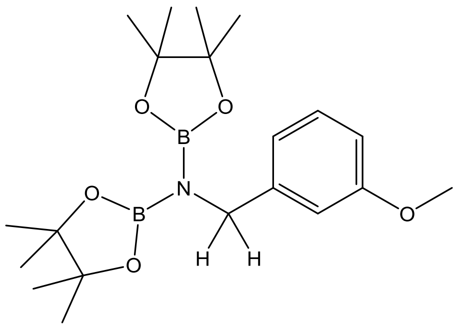

NMR scale: 24.5 µL 3-(methoxy)benzonitrile, 60 °C for 15 hr. ^{1}H NMR (300 MHz, C_6D_6, 298 K): 7.26 (1H, m, *o*-C*H*), 7.25 (1H, m, *o*-C*H*), 6.78 (1H, m, *p*-C*H*), 6.64 (1H, m, *m*-C*H*), 4.64 (2H, s, NC*H*$_2$), 3.41 (3H, s, OC*H*$_3$), 1.05 (24H, s, OC(C*H*$_3$)$_2$). ^{13}C{^{1}H} NMR (75.5 MHz, C_6D_6, 298 K): 129.7 (*o-C*), 128.7 (*o-C*), 128.5 (*p-C*), 128.3 (*m-C*, 82.9 (O*C*(CH$_3$)$_2$), 55.0 (O*C*H$_3$), 48.3 (N*C*H$_2$), 25.1 (OC(*C*H$_3$)$_2$). ^{11}B NMR (96.3 MHz, C_6D_6, 298 K): 30.0 N*B*. **Scale up:** 122.3 µL of 3-(methoxy)benzonitrile, 60 °C for 15 hrs. Isolated as pale yellow crystals (308 mg, 81% yield). An accurate microanalysis could not be obtained for this compound.

N-{B(OCMe$_2$)$_2$} - p-**tolylmethanamine**

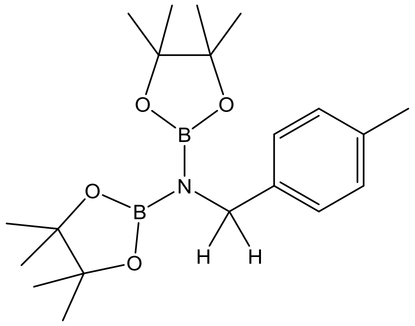

NMR scale: 23.9 µL *p*-tolunitrile, 60 °C for 13 hr. ^{1}H NMR (300 MHz, C_6D_6, 298 K): 7.50 (2H, d, J_{HH} = 9 Hz, *o-H*), 7.06 (2H, d, J_{HH} = 9 Hz, *m-H*), 4.58 (2H, s, NC*H*$_2$), 2.15 (3H, s, *p*-C*H*$_3$), 1.04 (24H, s, OC(C*H*$_3$)$_2$). ^{13}C{^{1}H} NMR (75.5 MHz, C_6D_6, 298 K): 141.2 (*o-C*), 136.1 (*p-C*), 129.4 (*i-C*), 128.5 (*m-C*), 82.9 (O*C*(CH$_3$)$_2$), 47.9 (N*C*H$_2$), 25.1 (OC(*C*H$_3$)$_2$), 21.5 (*p-C*H$_3$). ^{11}B NMR (96.3 MHz, C_6D_6, 298 K): 29.7 N*B*. **Scale up:** 119.4 µL of *p*-tolunitrile, 60 °C for 15 hrs. Isolated as colorless crystals (270 mg, 72% yield). Elemental analysis: calcd. (found) for $C_{20}H_{33}B_2NO_4$: C 64.38 (64.20); H 8.92 (8.80); N 3.75 (3.87).

***N-{B(OCMe$_2$)$_2$} – 4-*(fluoro)phenylmethanamine**

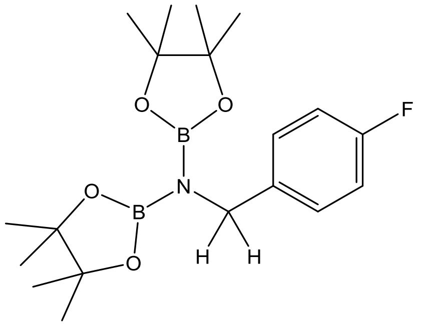

NMR scale: 21.9 μL 4-(fluoro)benzonitrile, 60 °C for 12 hr. ^{1}H NMR (300 MHz, C_6D_6, 298 K): 7.40 (2H, m, *o-H*), 6.88 (2H, t, J_{HH} = 9 Hz, *m*-H), 4.45 (2H, s, NC*H$_2$*), 1.02 (24H, s, OC(C*H*$_3$)$_2$). ^{13}C{^{1}H} NMR (75.5 MHz, C_6D_6, 298 K): 164.2 (*p-C*), 139.9 (*o-C*), 130.1 (*i-C*), 115.5 (*m-C*), 82.9 (O*C*(CH$_3$)$_2$), 47.5 (N*C*H$_2$), 25.1 (OC(*C*H$_3$)$_2$). ^{11}B NMR (96.3 MHz, C_6D_6, 298 K): 29.7 N*B*. ^{19}F NMR (376.5 MHz, C_6D_6, 298 K) δF (ppm): -116.89 4-*F*. **Scale up:** 109.4 μL of 4-(fluoro)benzonitrile, 60 °C for 15 hrs. Isolated as colorless crystals (230 mg, 61% yield). Elemental analysis: calcd. (found) for $C_{19}H_{30}B_2FNO_4$: C 60.52 (60.55); H 8.02 (7.93); N 3.74 (3.83).

***N-{B(OCMe$_2$)$_2$} – 4-*(Chloro)phenylmethanamine**

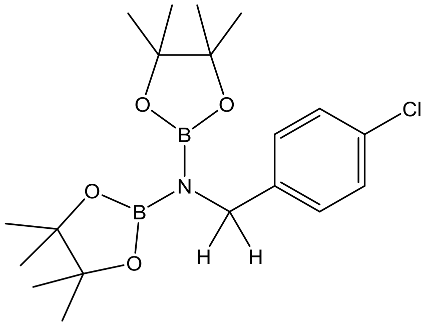

NMR scale: 27.5 mg 4-(chloro)benzonitrile, 60 °C for 12 hr. ^{1}H NMR (300 MHz, C_6D_6, 298 K): 7.36 (2H, d, J_{HH} = 7.3 Hz, *o*-C*H*), 7.20 (2H, d, J_{HH} = 7.3 Hz, *m*-C*H*), 4.46 (2H, s, NC*H$_2$*), 1.02 (24H, s, OC(C*H$_3$*)$_2$). ^{13}C{^{1}H} NMR (75.5 MHz, C_6D_6, 298 K): 129.9 (*o-C*), 128.9 (*p-C*), 128.7 (*m-C*), 83.0 (O*C*(CH$_3$)$_2$), 47.5 (N*C*H$_2$), 25.1 (OC(*C*H$_3$)$_2$). ^{11}B NMR (96.3 MHz, C_6D_6, 298 K): 29.0 (N*B*). **Scale up:** 137.6 mg of 4-(chloro)benzonitrile, 60 °C for 12 hrs. Isolated as pale yellow crystals (231 mg, 59% yield). Elemental analysis: calcd. (found) for $C_{19}H_{30}B_2ClNO_4$: C 57.99 (57.47); H 7.68 (7.40); N 3.56 (3.52).

***N*-{B(OCMe$_2$)$_2$} – *4*-(trifluoromethyl)phenylmethanamine**

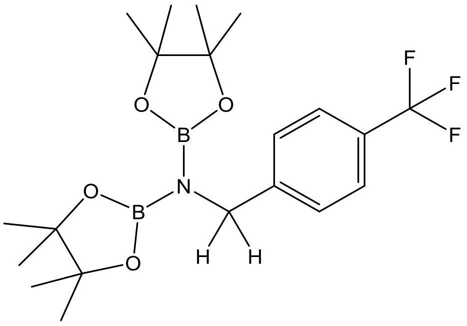

NMR scale: 26.8 µL 4-(trifluoromethyl)benzonitrile, 60 °C for 12.5 hr. ^{1}H NMR (300 MHz, C_6D_6, 298 K): 7.41 (4H, s, Ar-*H*), 4.46 (2H, s, NC*H*$_2$), 1.01 (24H, s, OC(C*H*$_3$)$_2$). ^{13}C{^{1}H} NMR (75.5 MHz, C_6D_6, 298 K): 148.1 (*o*-*C*), 128.5 (*p*-C), 125.6 (*m*-*C*), 83.1 (O*C*(CH$_3$)$_2$), 47.8 (N*C*H$_2$), 25.0 (OC(*C*H$_3$)$_2$). ^{11}B NMR (96.3 MHz, C_6D_6, 298 K): 29.6 N*B*. ^{19}F NMR (376.5 MHz, C_6D_6, 298 K) δF (ppm): -61.94 C*F*$_3$. **Scale up:** 133.9 µL of 4-(trifluoromethyl)benzonitrile, 60 °C for 15 hrs. Isolated as colorless crystals (325 mg, 76% yield). An accurate microanalysis could not be obtained for this compound.

***N*-{B(OCMe$_2$)$_2$} – *4*-(methoxy)phenylmethanamine**

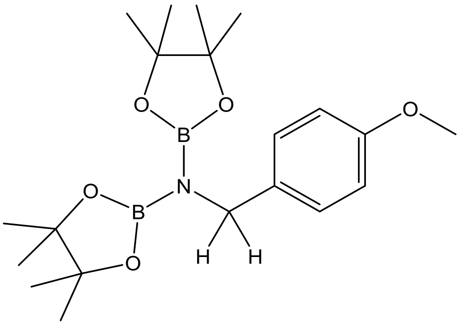

NMR scale: 26.6 mg 4-methoxybenzonitrile, 60 °C for 13.5 hr. ^{1}H NMR (300 MHz, C_6D_6, 298 K): 7.53 (2H, d, J_{HH} = 6 Hz, *o-H*), 6.85 (2H, d, J_{HH} = 9 Hz, *m-H*), 4.54 (2H, s, NC*H*$_2$), 3.36 (3H, s, OC*H*$_3$), 1.04 (24H, s, OC(C*H*$_3$)$_2$). ^{13}C{^{1}H} NMR (75.5 MHz, C_6D_6, 298 K): 159.3 (*p-C*), 136.4 (*o-C*), 129.8 (*i-C*), 114.2 (*m-C*), 82.9 (O*C*(CH$_3$)$_2$), 55.1 (O*C*H$_3$), 47.6 (N*C*H$_2$), 25.1 (OC(*C*H$_3$)$_2$). ^{11}B NMR (96.3 MHz, C_6D_6, 298 K): 29.7 N*B*. **Scale up:** 133.2 mg of 4-methoxybenzonitrile, 60 °C for 15 hrs. Isolated as colorless crystals (225 mg, 58% yield). Elemental analysis: calcd. (found) for $C_{20}H_{33}B_2NO_4$: C 61.74 (61.60); H 8.55 (8.50); N 3.60 (3.50).

***N*-{B(OCMe$_2$)$_2$} – diphenylacetoamine**

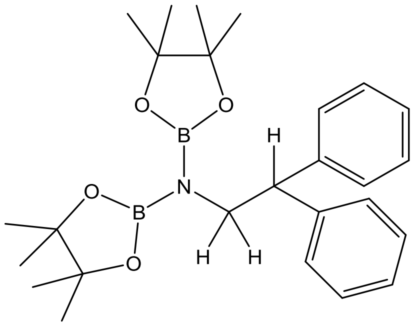

NMR scale: 38.6 mg diphenylacetonitrile, 60 °C for 30 hr. ^{1}H NMR (300 MHz, C_6D_6, 298 K): 7.43 (4H, d, J_{HH} = 6 Hz, *o-H*), 7.05 (4H, m, *m-H*), 6.94 (2H, m, *p-H*), 4.65 (1H, t, J_{HH} = 6 Hz, NCH$_2$C*H*), 4.10 (2H, d, J_{HH} = 9 Hz, NC*H$_2$*CH), 1.02 (24H, s, C(C*H$_3$*)$_2$). ^{13}C{^{1}H} NMR (75.5 MHz, C_6D_6, 298 K): 144.28 (*ipso-C*), 129.62 (*o-C*), 128.87 (*p-C*), 126.71 (*m-C*), 82.70 (*C*(CH$_3$)$_2$), 54.44 (NCH$_2$*C*H), 49.55 (N*C*H$_2$CH), 25.07 (C(*C*H$_3$)$_2$). ^{11}B NMR (96.3 MHz, C_6D_6, 298 K): 29.6 N*B*. **Scale up:** 133.2 mg of diphenylacetonitrile, 60 °C for 30 hrs. Isolated as colorless crystals (199 mg, 43% yield). An accurate microanalysis could not be obtained for this compound.

^{1}H and ^{13}C{^{1}H} NMR spectra

N-{B(OCMe$_2$)$_2$} -propan-1-amine

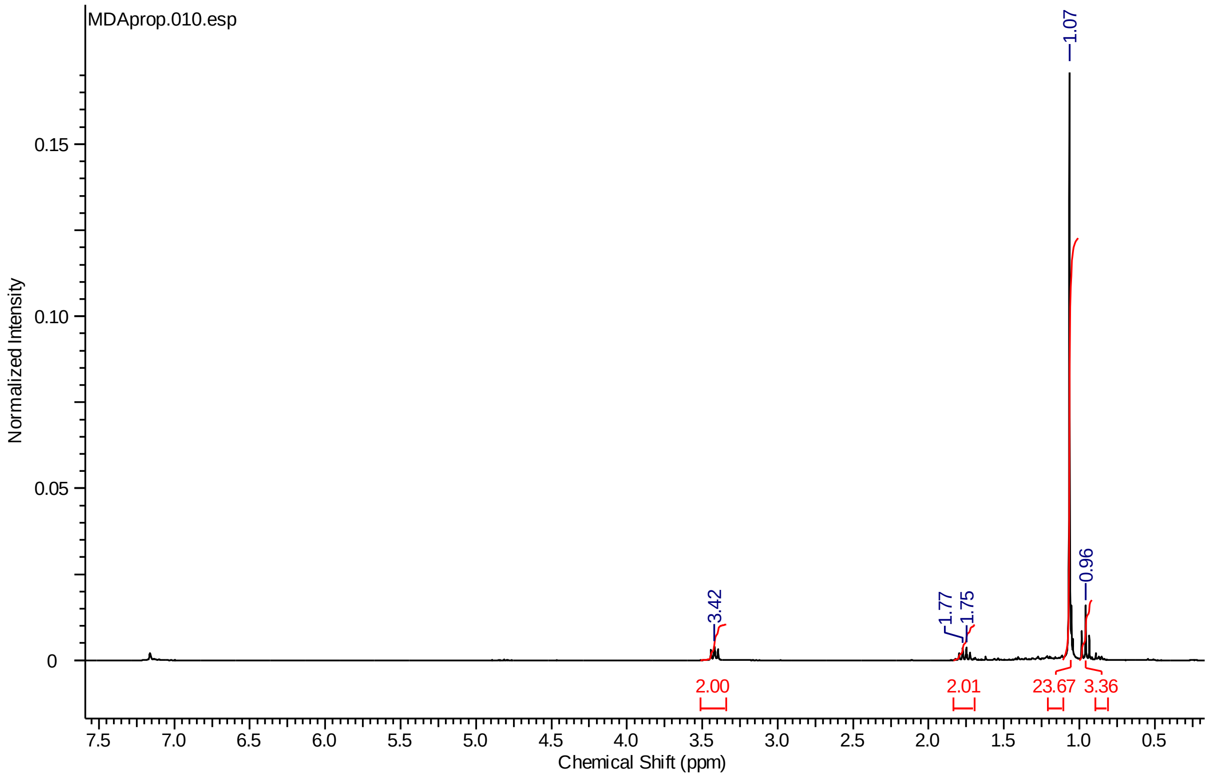

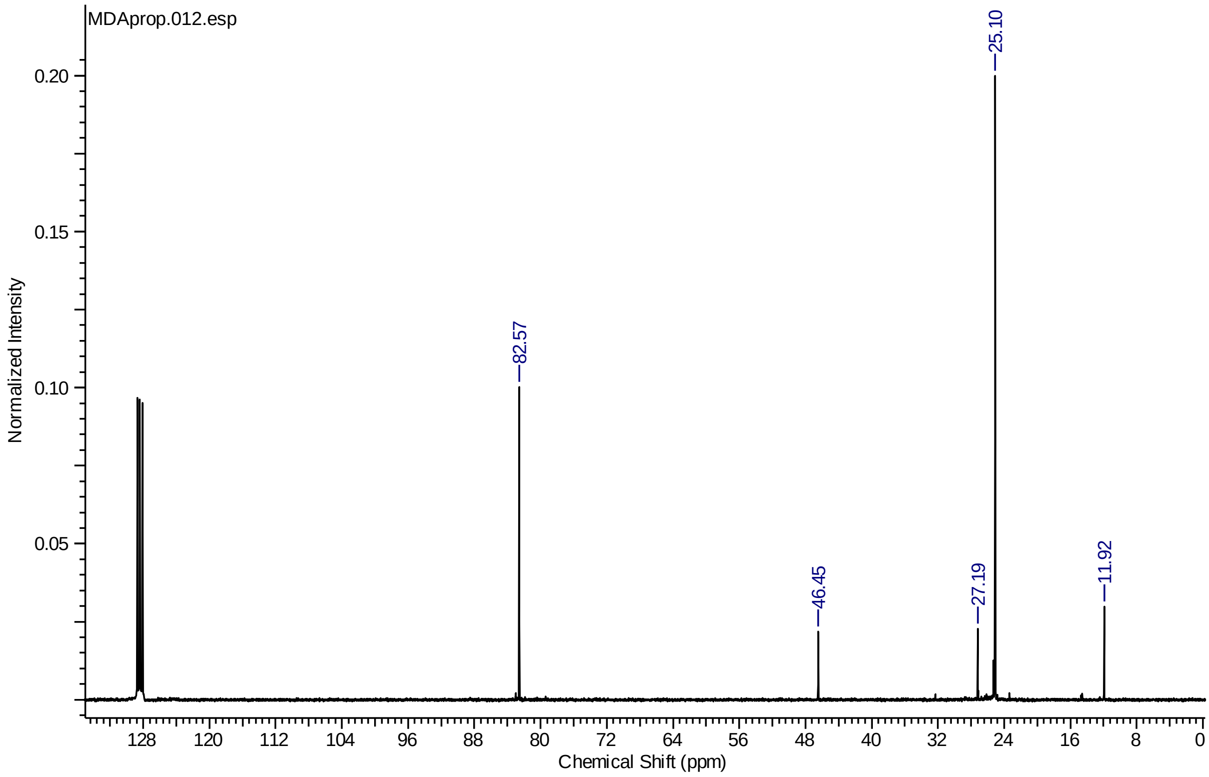

***N*-{B(OCMe$_2$)$_2$} - 2-methylpropan-1-amine**

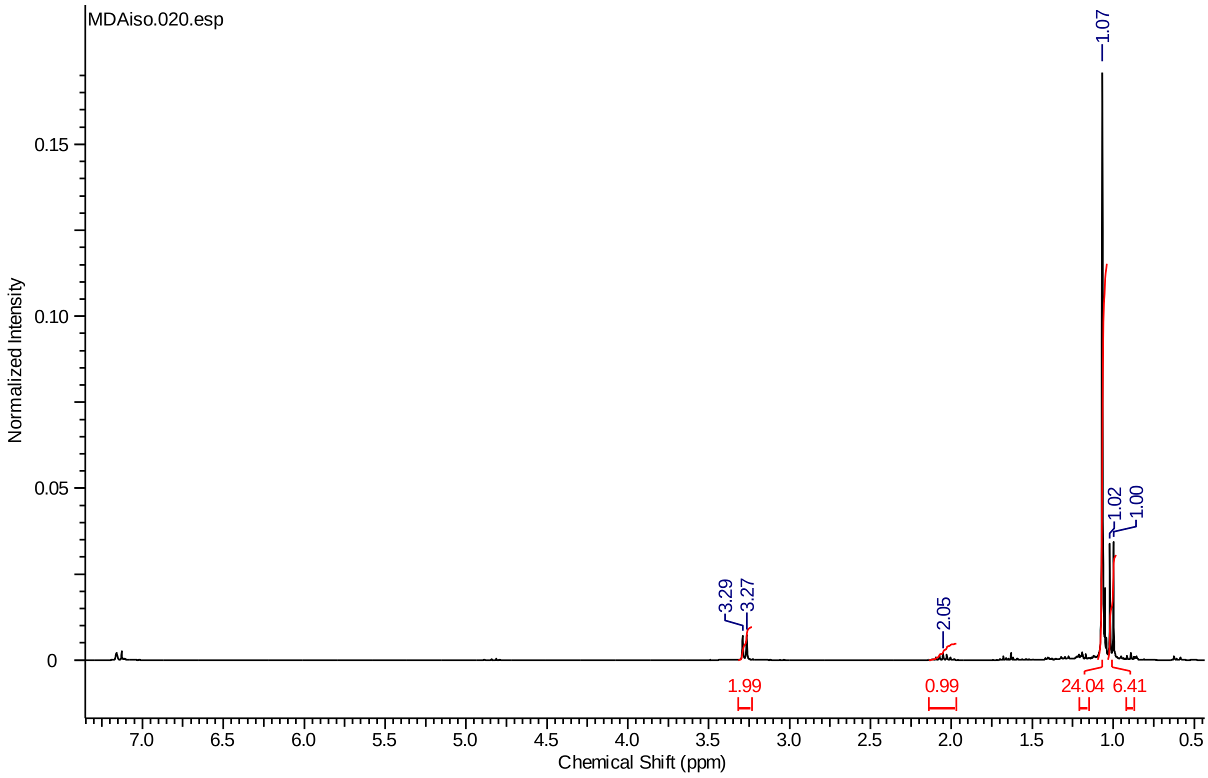

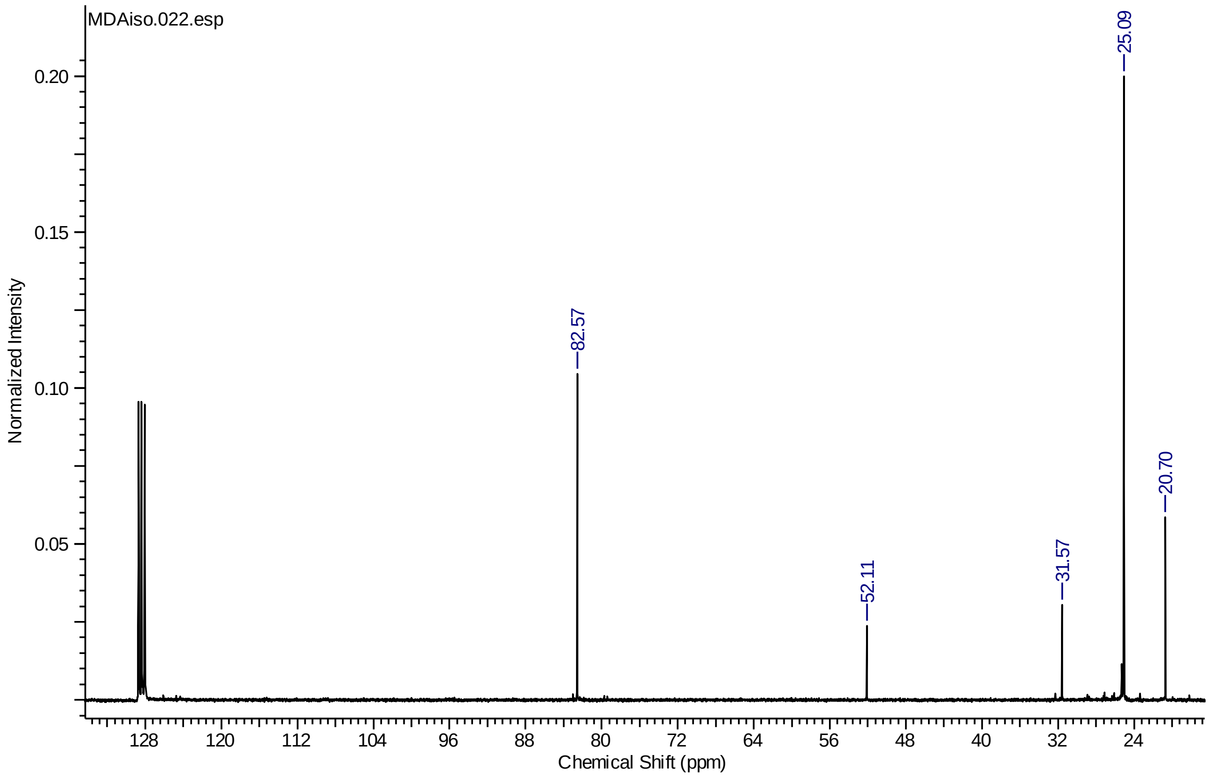

***N*-{B(OCMe$_2$)$_2$} - 2,2-dimethylpropan-1-amine**

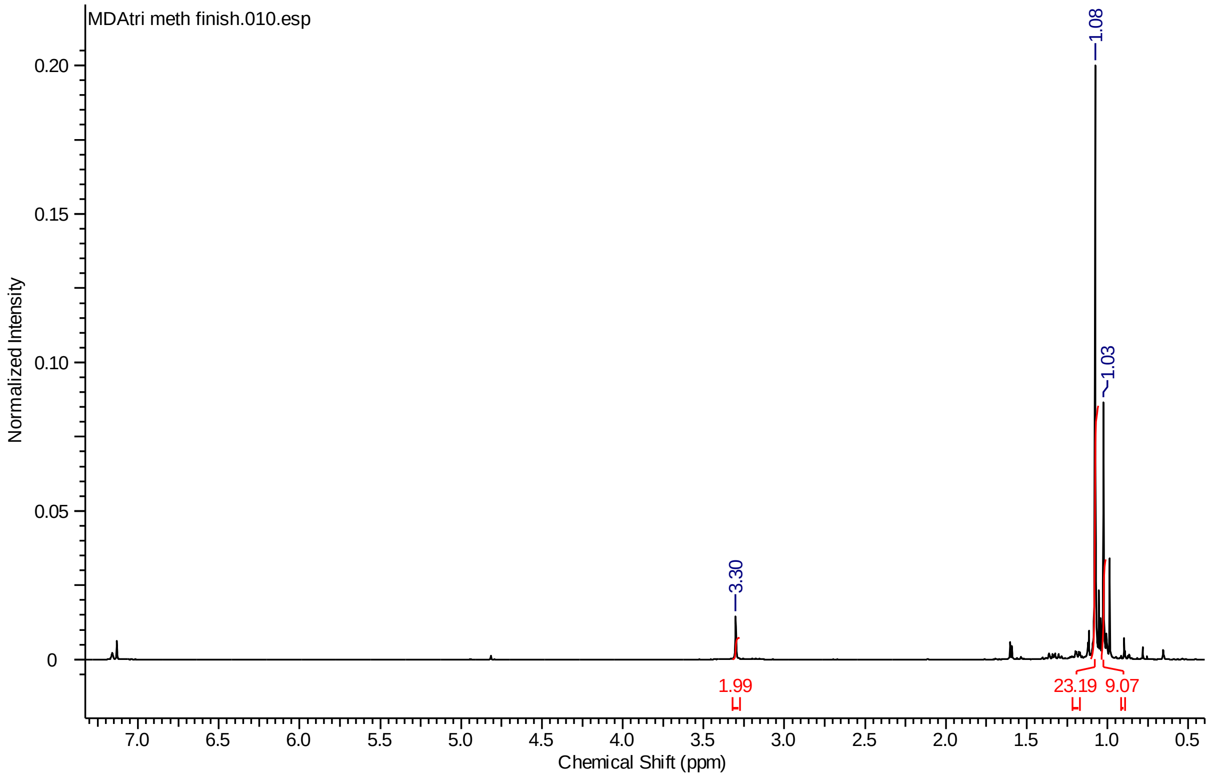

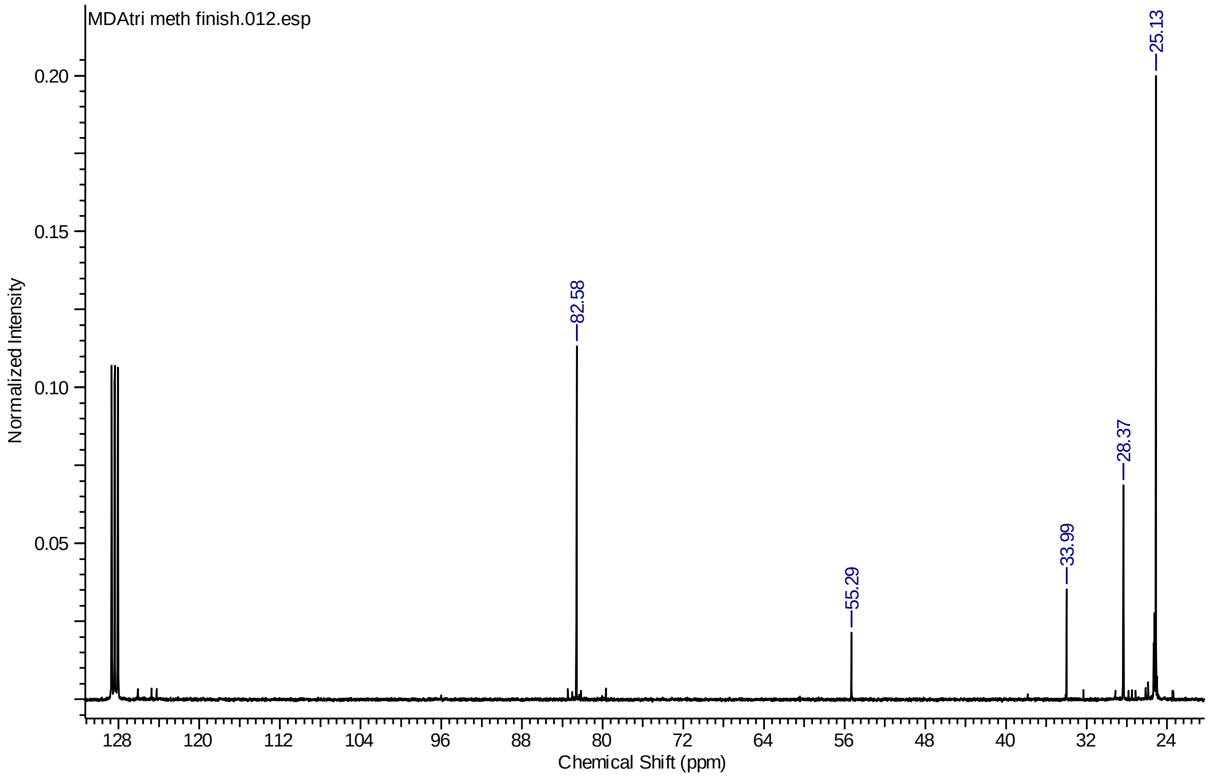

***N*-{B(OCMe$_2$)$_2$}** – **cyclohexylmethanamine**

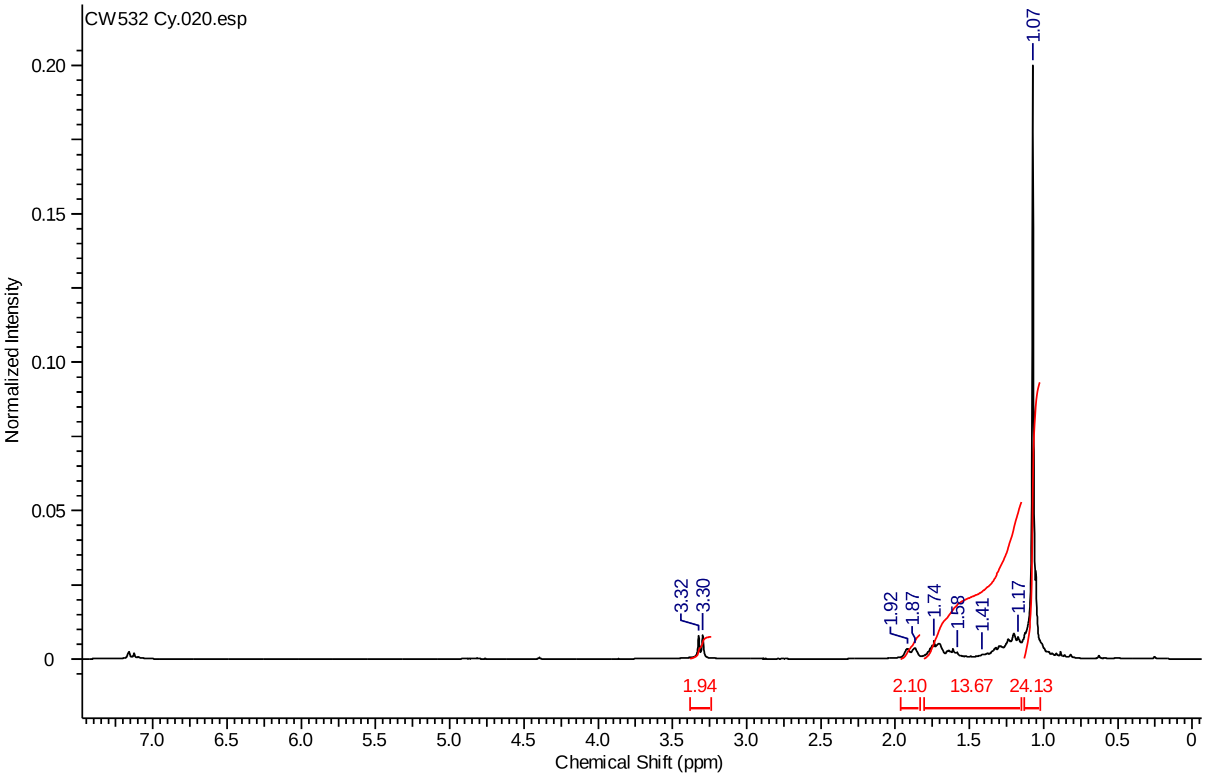

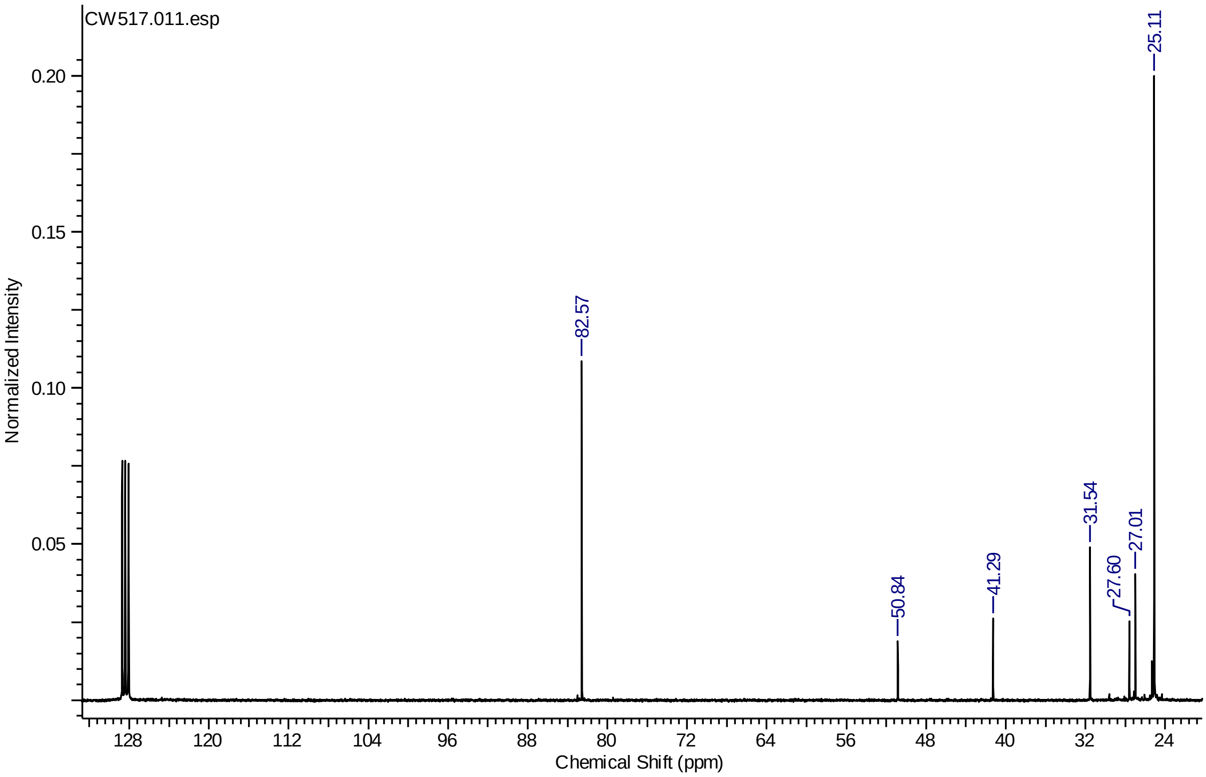

N-{B(OCMe$_2$)$_2$} – phenylmethanamine

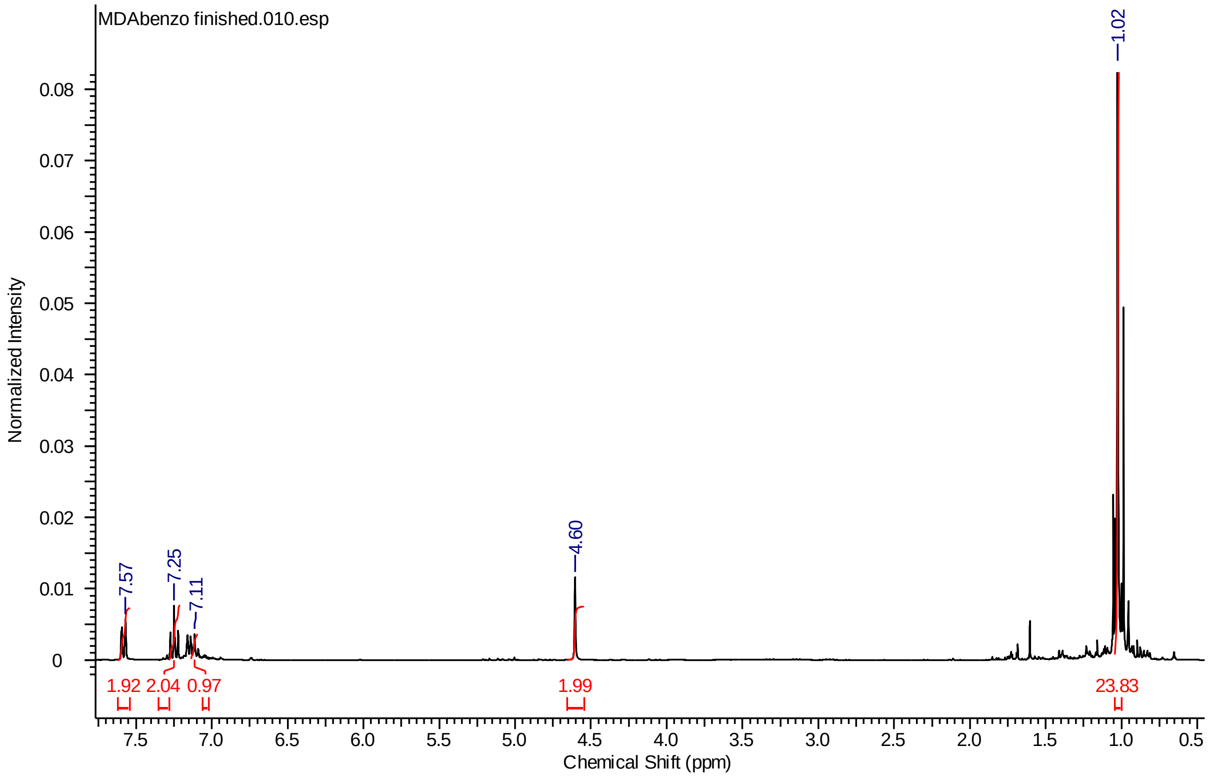

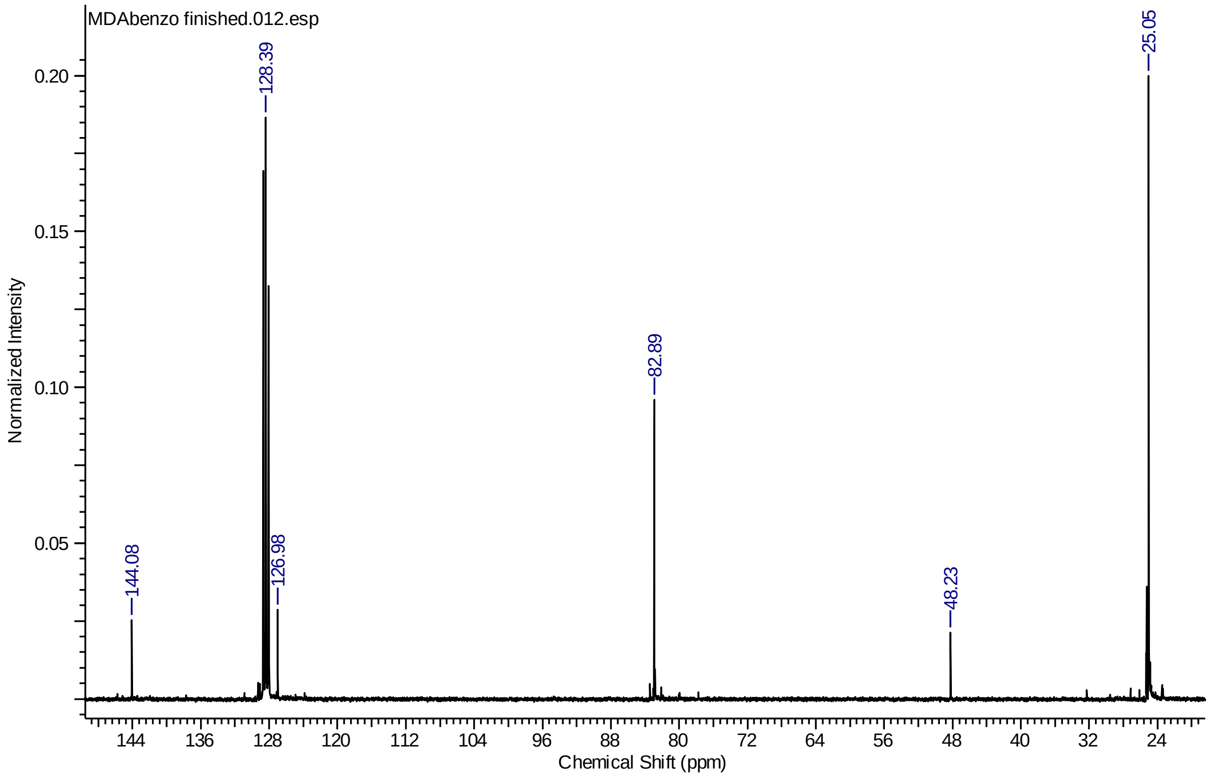

***N*-{B(OCMe$_2$)$_2$} - *o*-tolylmethanamine**

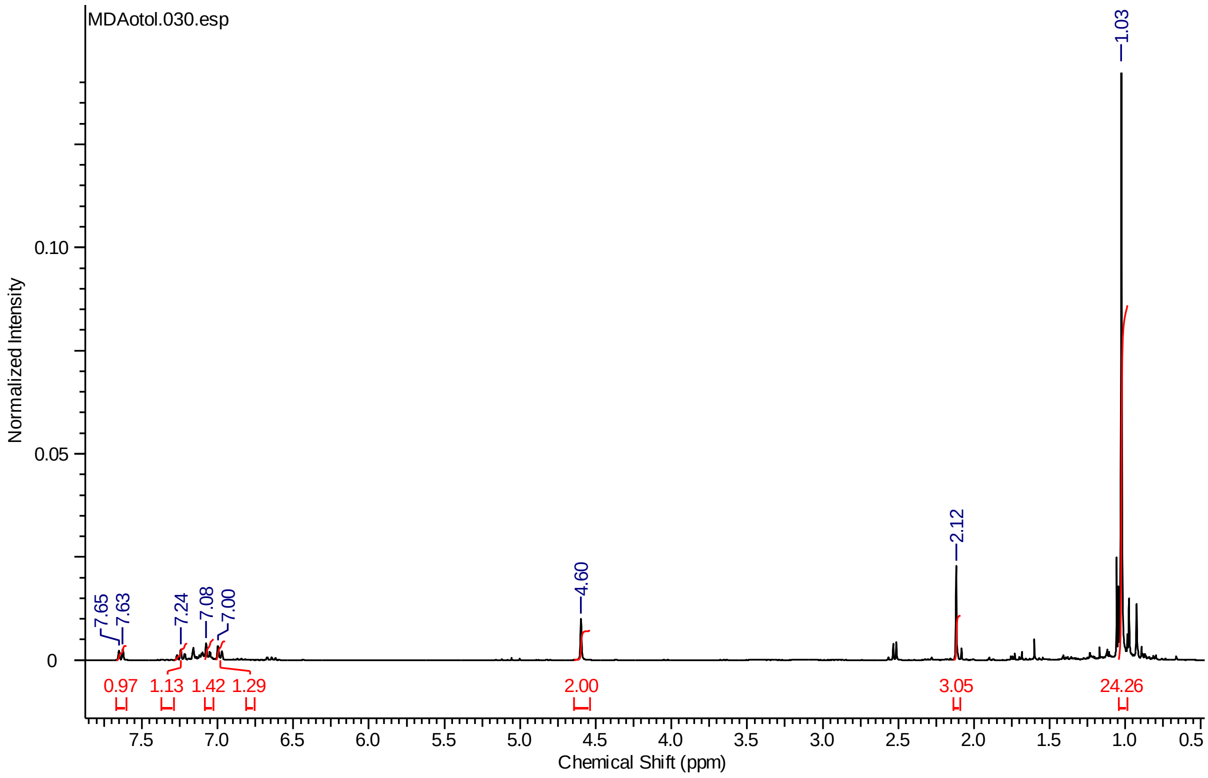

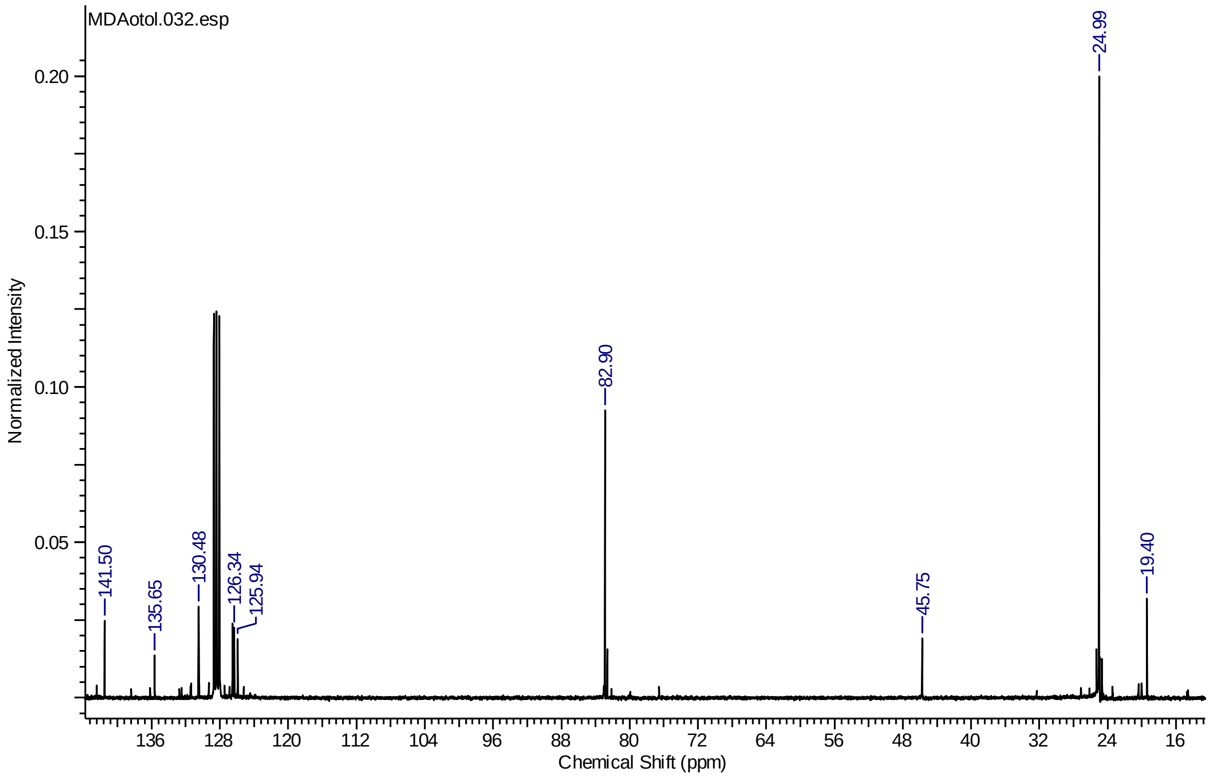

***N*-{B(OCMe$_2$)$_2$} - *m*-tolylmethanamine**

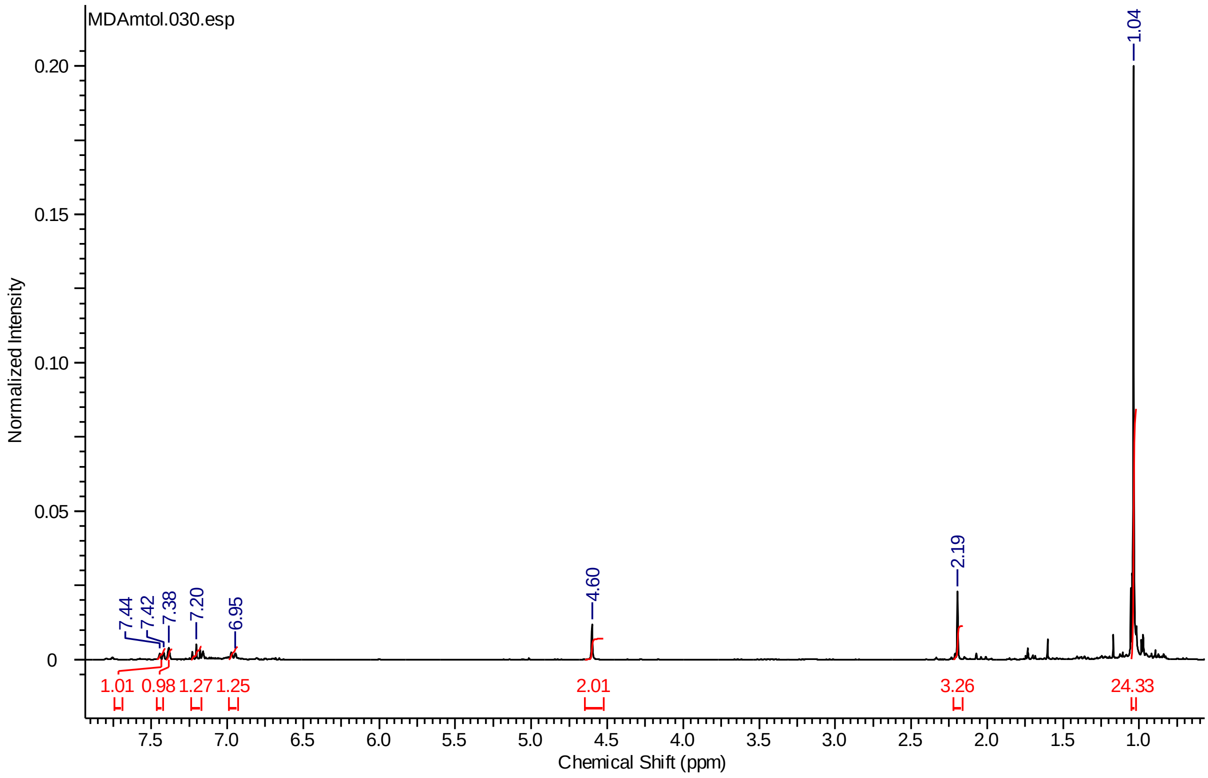

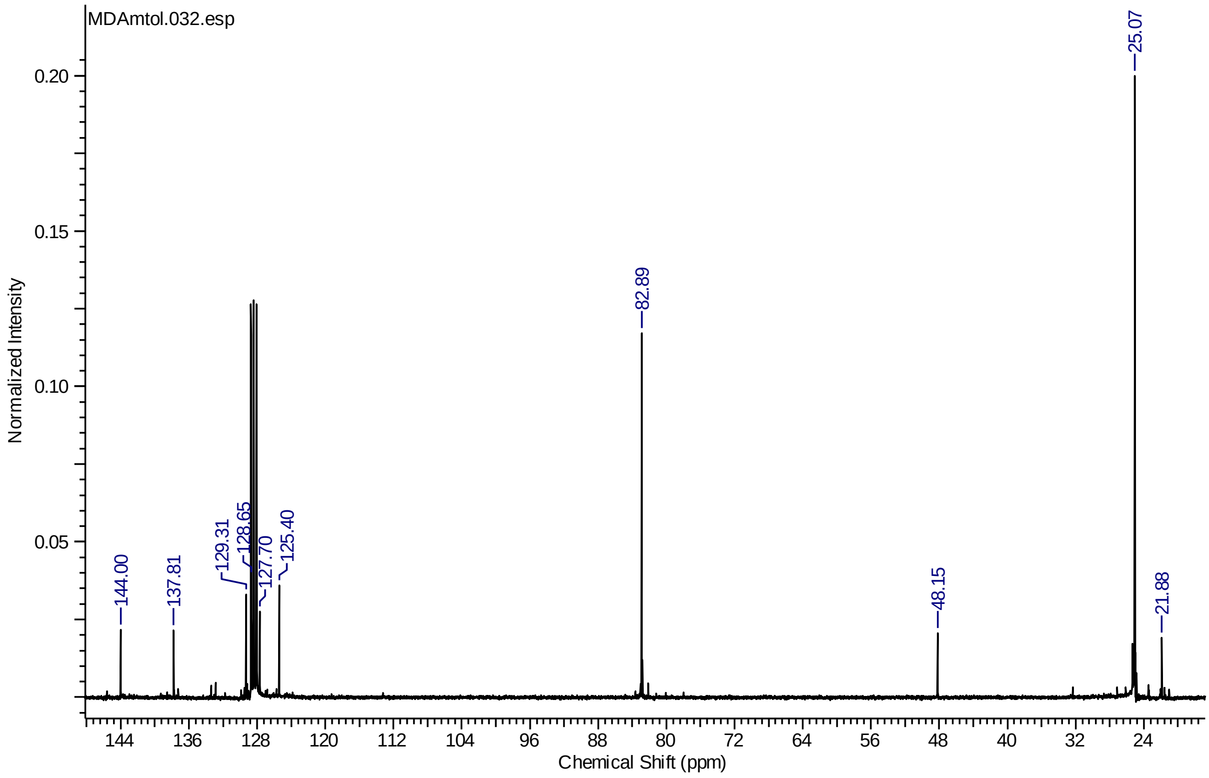

N-{B(OCMe$_2$)$_2$}-*p*-tolylmethanamine

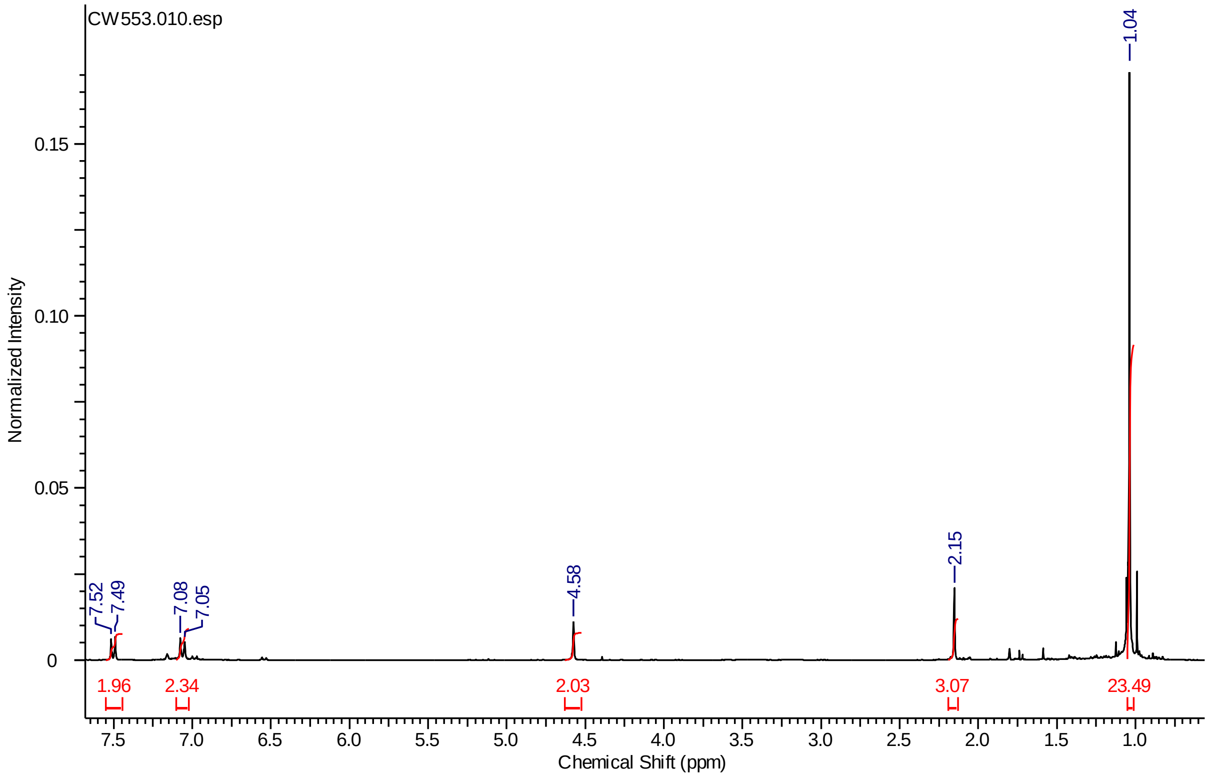

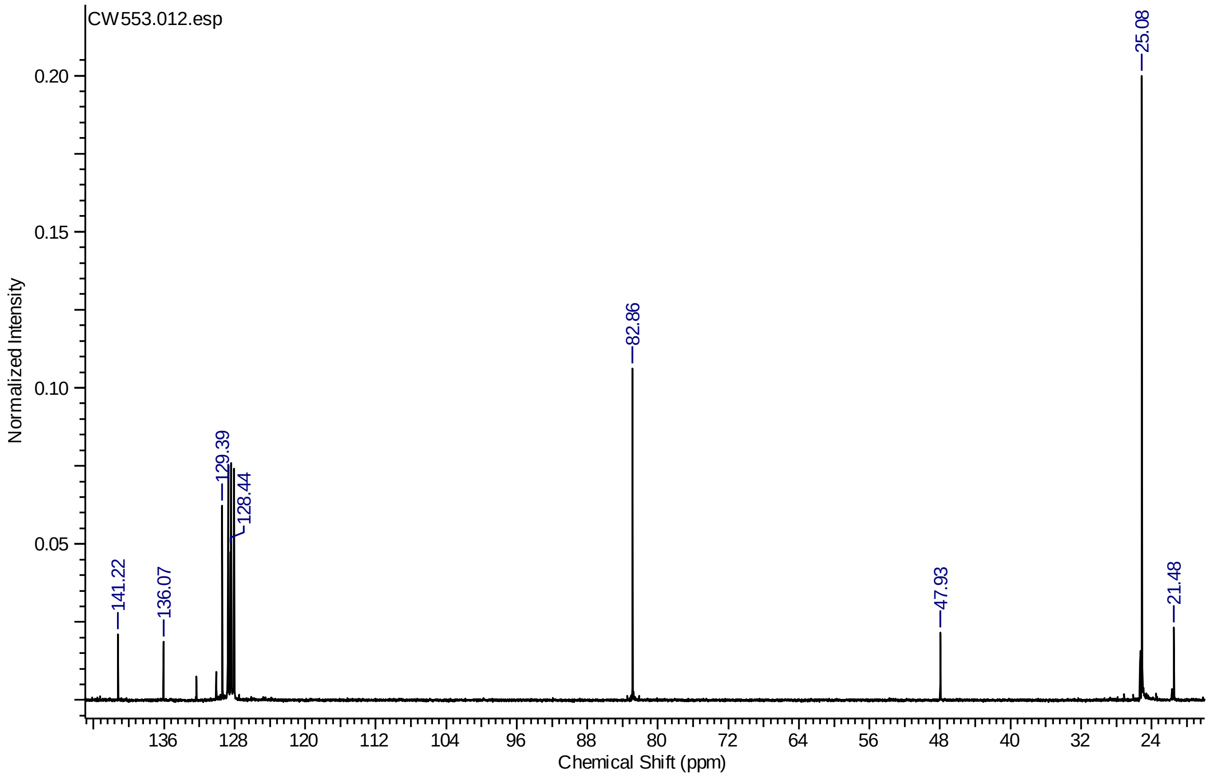

***N*-{B(OCMe$_2$)$_2$}-4-fluorophenylmethanamine**

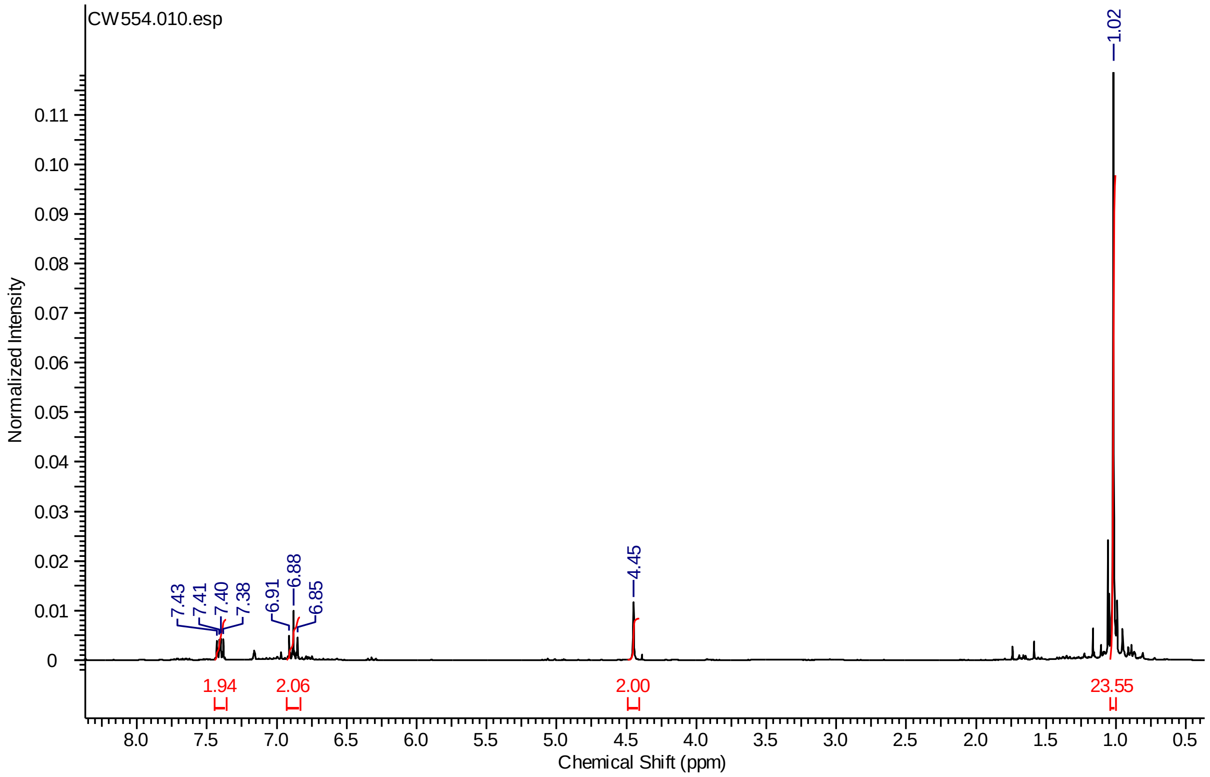

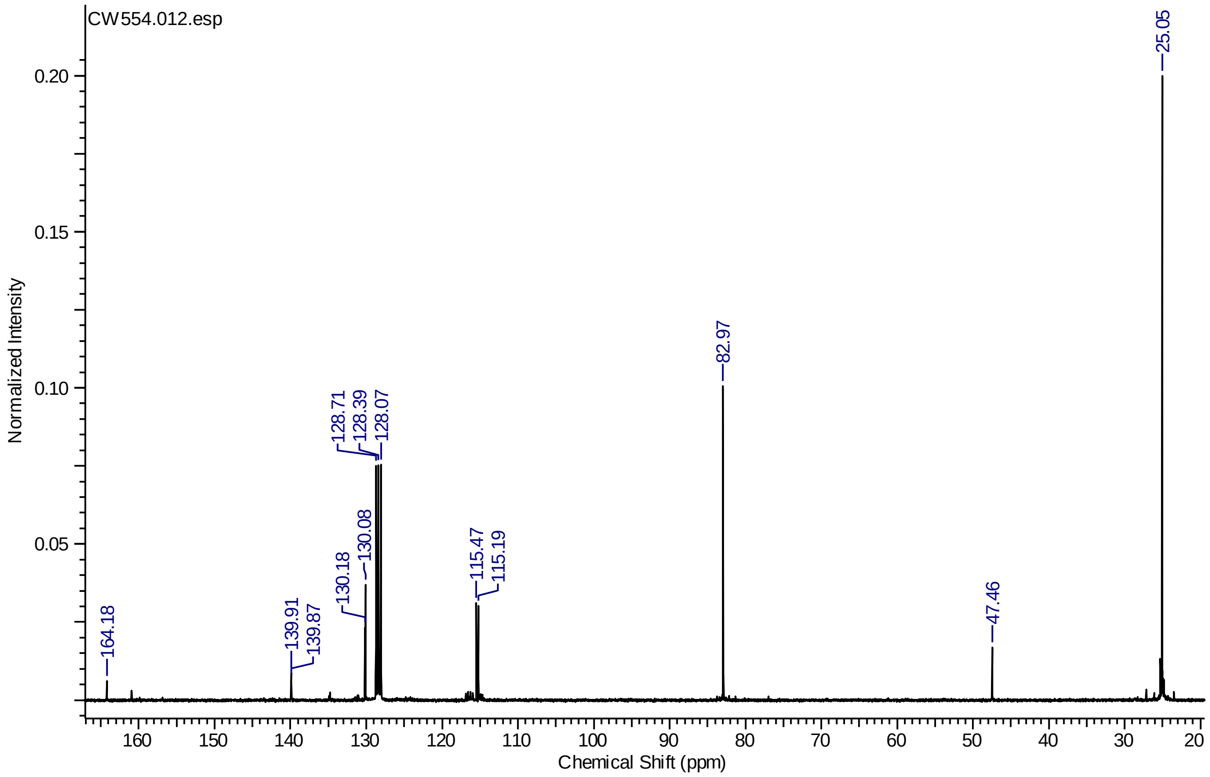

N-{B(OCMe$_2$)$_2$}-4-(trifluoromethyl)phenylmethanamine

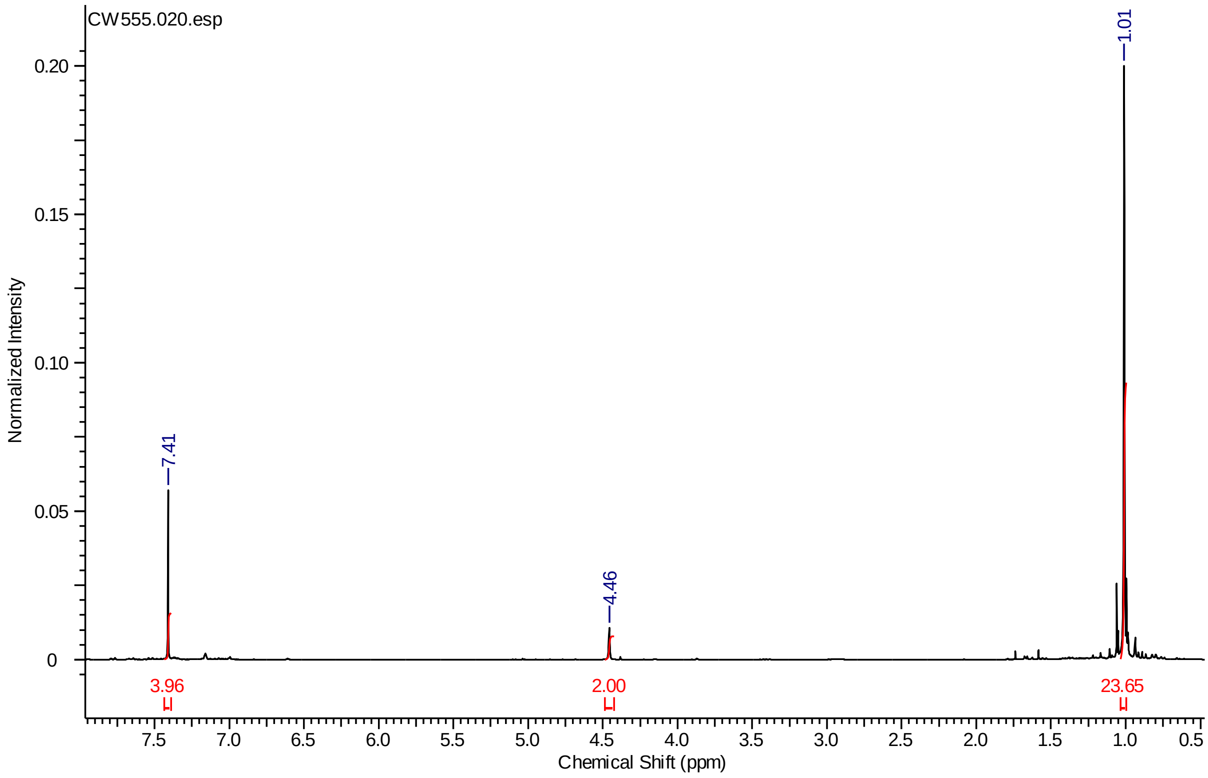

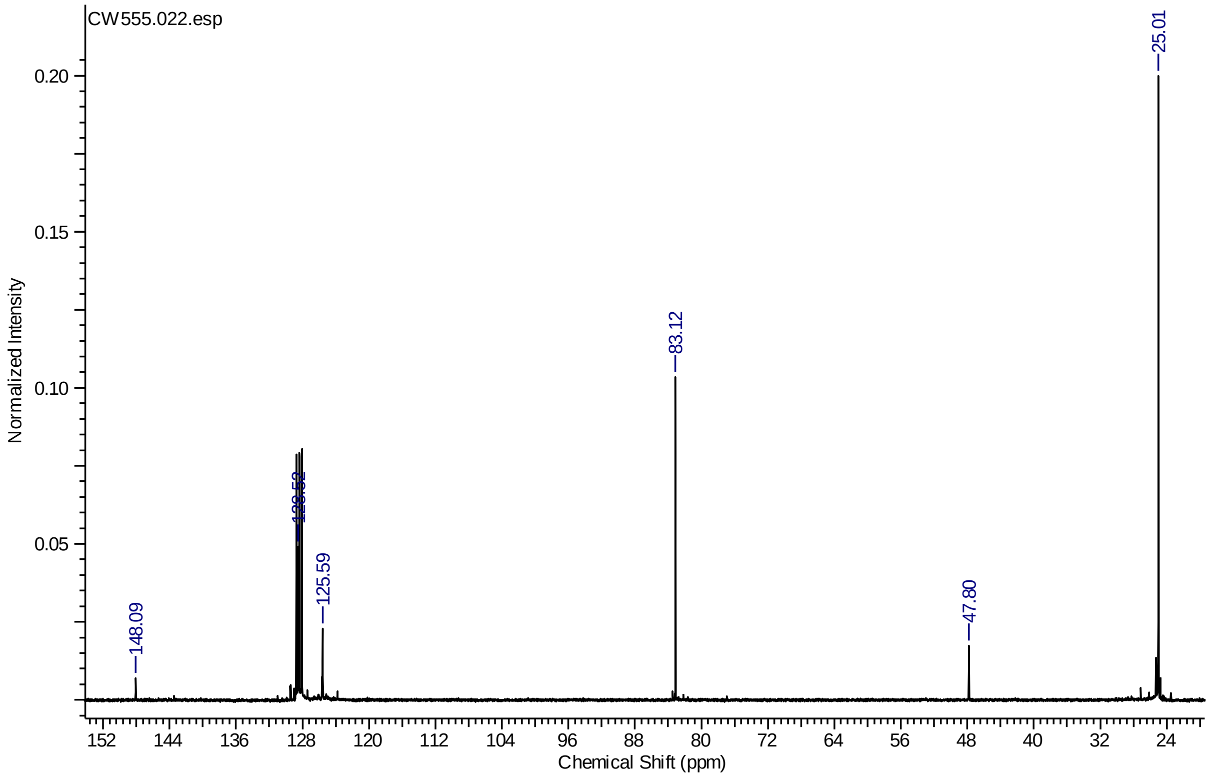

N-{B(OCMe$_2$)$_2$} – 4-(Methoxy)phenylmethanamine

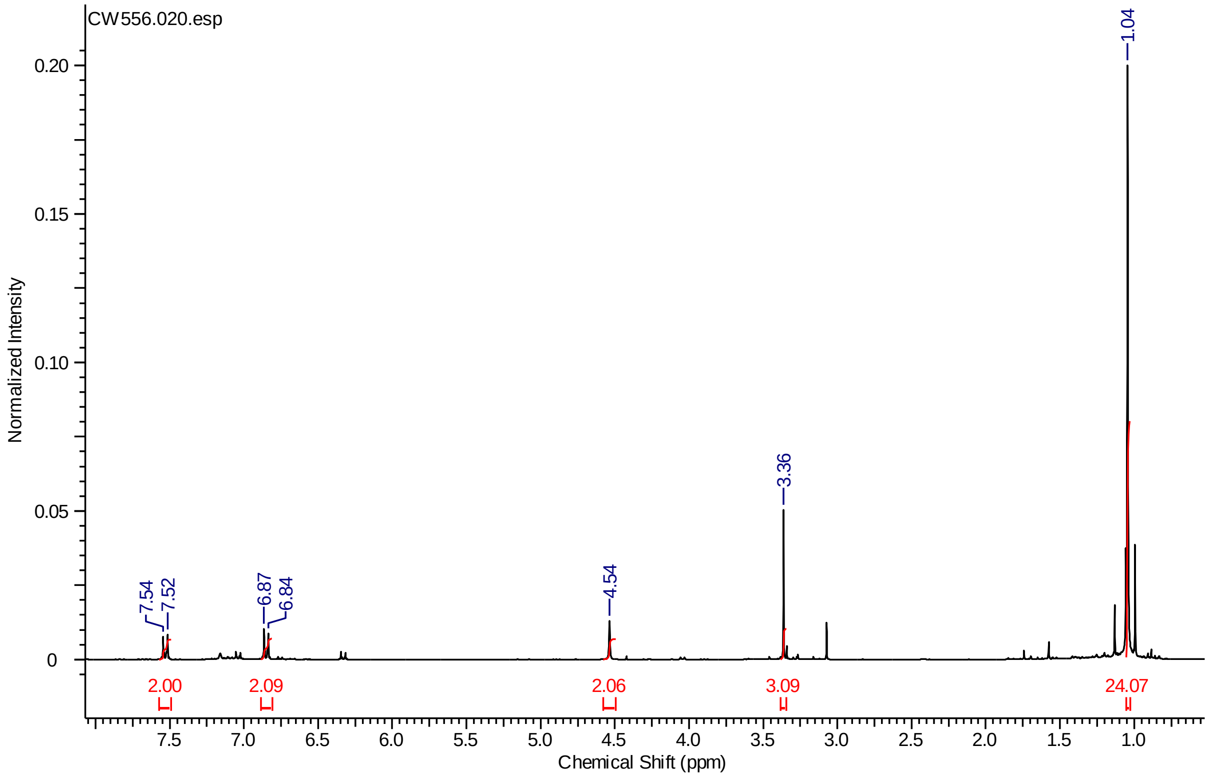

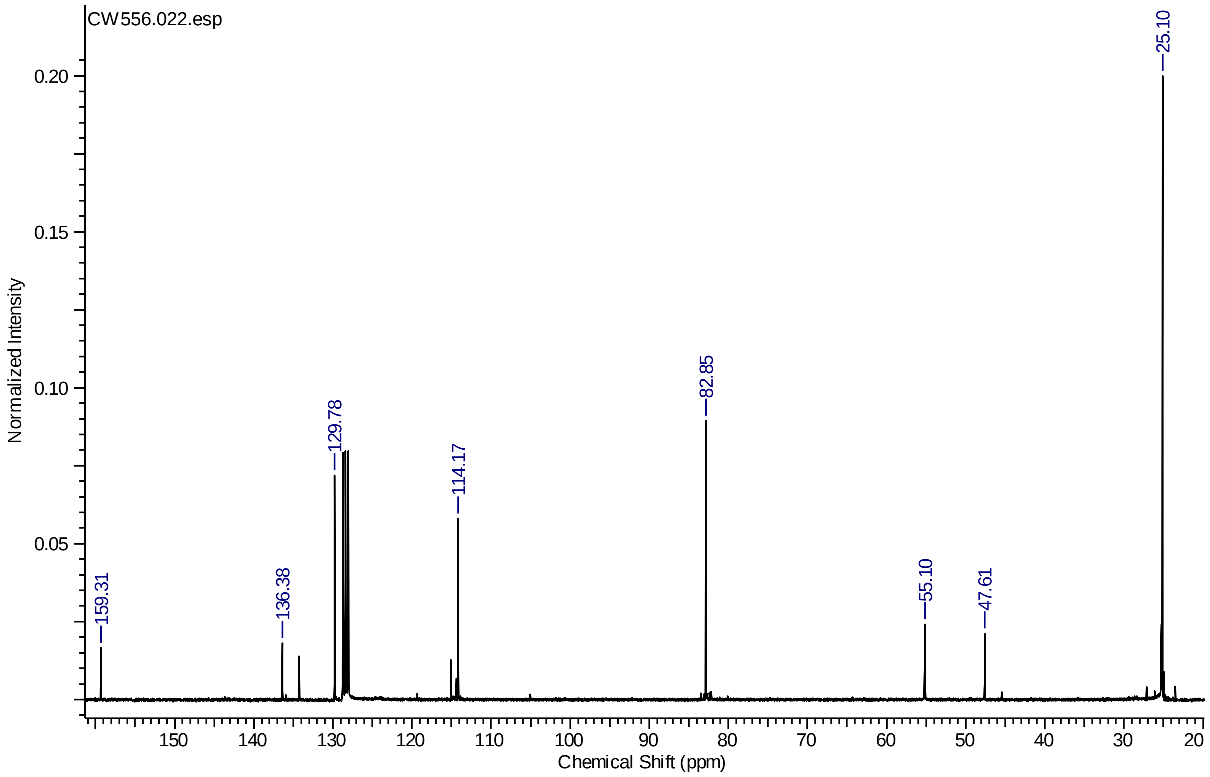

N-{B(OCMe$_2$)$_2$} – diphenylacetoamine

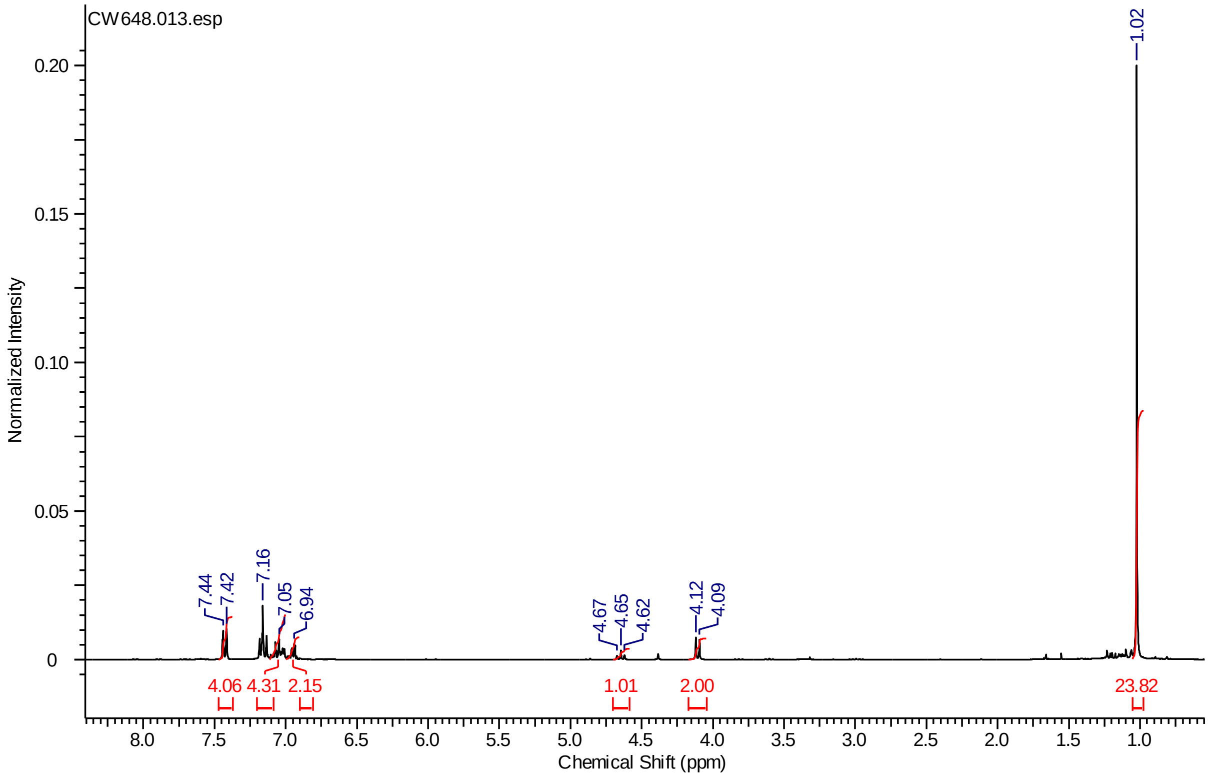

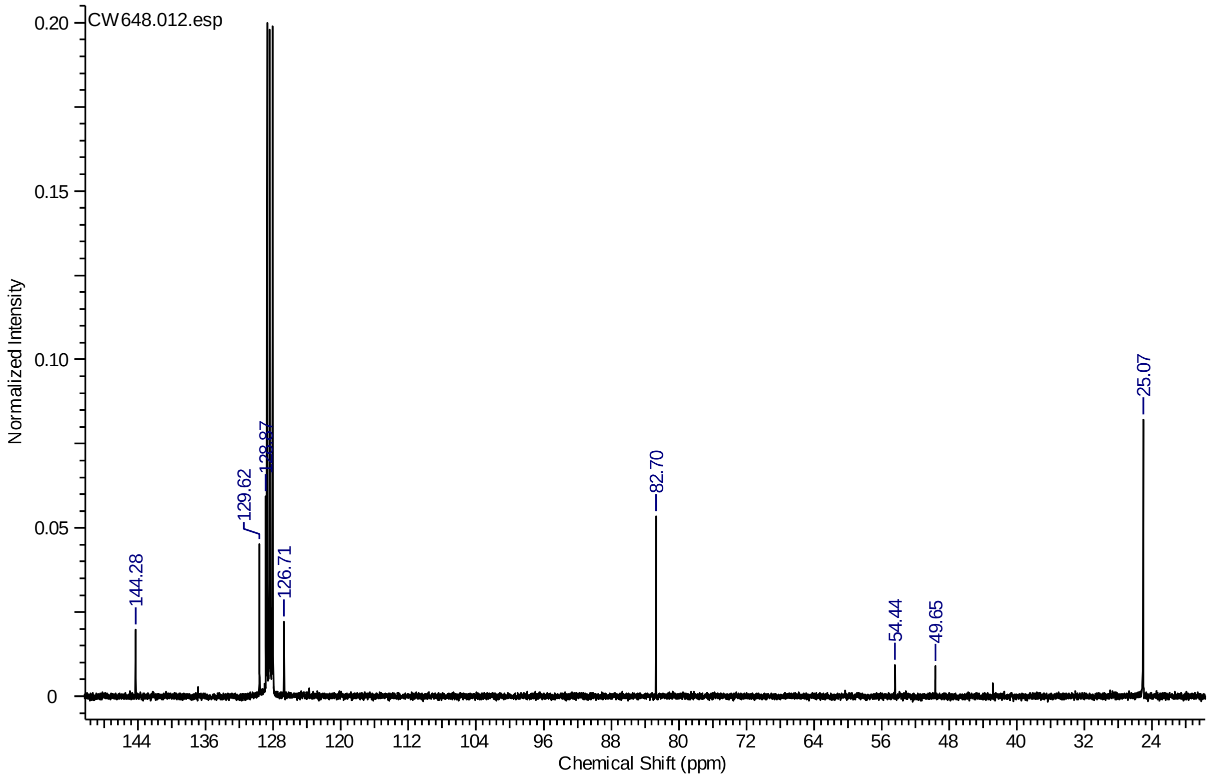

X-ray Structural Analyses

Diffraction data for compounds **1** – **5** were collected on a Nonius Kappa CCD with a low temperature device at 150 K, utilizing Mo-Kα radiation monochromated with graphite (λ = 0.71070 Å). Processing utilized the Nonius software,[2] with structure solution and refinement using XSeed[3] or WINGX-1.70[4] suite of programs throughout and visualized utilizing ORTEP 3.[5] The asymmetric unit of **2** comprises half of a dimer which straddles a crystallographic inversion center. Compound **5** co-crystallized with one molecule of toluene and a molecule of hexane which was half occupied. All bond lengths in the hexane molecule have been restrained. The phenyl group in the toluene molecule has been refined using constraints.

References

1. A. P. Dove, V. C. Gibson, P. Hormnirun, E. L. Marshall, J. A. Segal, A. J. P. White and D. J. Williams, *Dalton. Trans.* 2003, 3088.

2. DENZO-SCALEPACK Z. Otwinowski and W. Minor, " Processing of X-ray Diffraction Data Collected in Oscillation Mode ", Methods in Enzymology, Volume 276: Macromolecular Crystallography, part A, p.307-326, 1997,C.W. Carter, Jr. & R. M. Sweet, Eds., Academic Press.

3. G. M. Sheldrick, *SHELXL97-2, Program for Crystal Structure Refinement*, Universität Göttingen, Göttingen, Germany, 1998.

4. L. J. Barbour, *X-Seed - A Software Tool for Supramolecular Crystallography, J. Supramol. Chem.* 2001, **1**, 189.

5. C. Barnes, *J. Appl. Cryst*. 1997, **30**, 568.

Kinetic Experiments

All NMR Data were recorded on a Bruker AV400 NMR operating at 400.13 MHz (^{1}H) and were recorded at 323 K unless stated otherwise. All data were processed using ACD/Labs group spectra common integral analysis software.

In a glovebox a stock solution of the precatalyst was made to the relevant concentration, 0.5 mL of the catalyst solution was transferred to a Youngs tap NMR tube followed by addition of the relevant quantity of HBpin, followed by the chosen substrate. The tube was sealed, removed from the glovebox, immediately frozen with liquid nitrogen and thawed just prior to loading into the NMR spectrometer which had been preheated to a chosen temperature (if required). ^{1}H NMR spectra were recorded at regular intervals. Reaction kinetics were monitored using the intensity changes in the substrate resonances over three or more half-lives on the basis of substrate consumption. Data was normalised against the initial substrate concentration [Substrate]$_{t=0}$ so that:

$$Ct = \frac{[Substrate]_{t=0}}{[Substrate]_{t=0} + [Substrate]_t}$$

Reaction rates were derived from the plot of Ct vs time (or Ln(Ct), 1/Ct) by using linear trendlines generated by Microsoft Excel software. To obtain Arrhenius and Eyring plots, kinetic analyses were conducted at 4-5 different temperatures, each separated by approximately 5 K.

Propionitrile Hydroboration Kinetics

Determination of Catalyst order

Figure S1 *Pseudo*-zero order kinetics of propionitrile dihydroboration with varying [**V**]

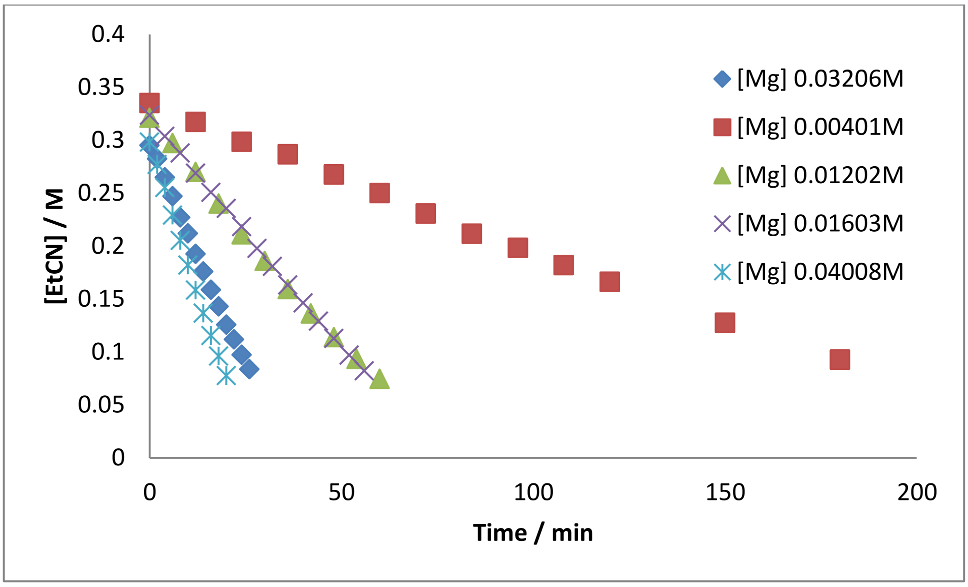

	[Mg] 0.03206 M	
	Value	Error
m_1	0.296000	0.001437
m_2	-0.008360	0.000094
Chisq	0.00020266	n/a
R^2	0.99804	n/a

	[Mg] 0.00401 M	
	Value	Error
m_1	0.331970	0.001682
m_2	-0.001370	0.000018
Chisq	0.001751	n/a
R^2	0.99804	n/a

	[Mg] 0.01202 M	
	Value	Error
m_1	0.317320	0.003780
m_2	-0.004210	0.000097
Chisq	0.001958	n/a
R^2	0.99601	n/a

	[Mg] 0.01063 M	
	Value	Error
m_1	0.321200	0.001232
m_2	-0.004340	0.000035
Chisq	0.000804	n/a
R^2	0.99951	n/a

	[Mg] 0.04008 M	
	Value	Error
m_1	0.297150	0.001701
m_2	-0.011260	0.000144
Chisq	0.000197	n/a
R^2	0.99854	n/a

Figure S2. ln([EtCN]$_0$/[EtCN]$_t$) vs time; non-linear kinetics

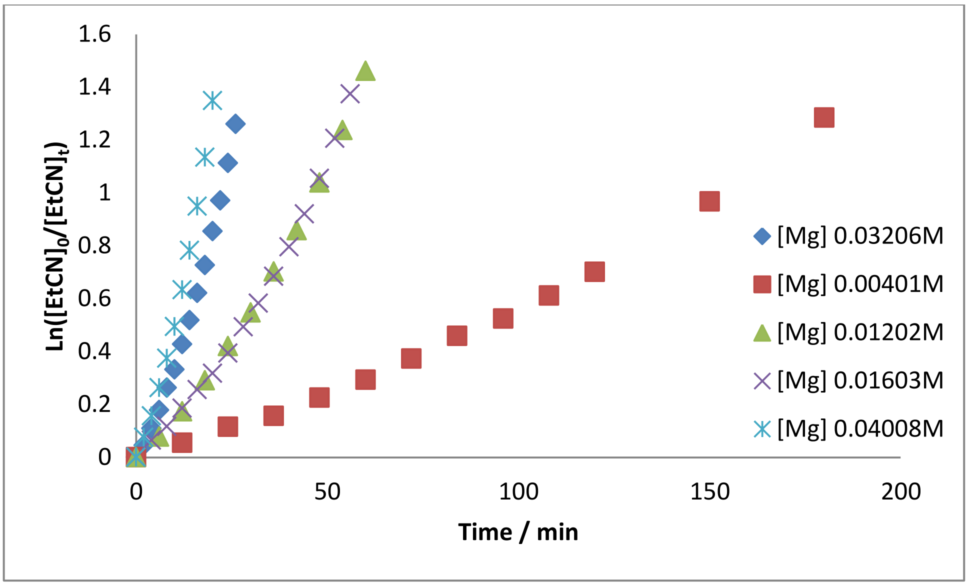

Figure S3. 1/[EtCN] vs time; non-linear kinetics

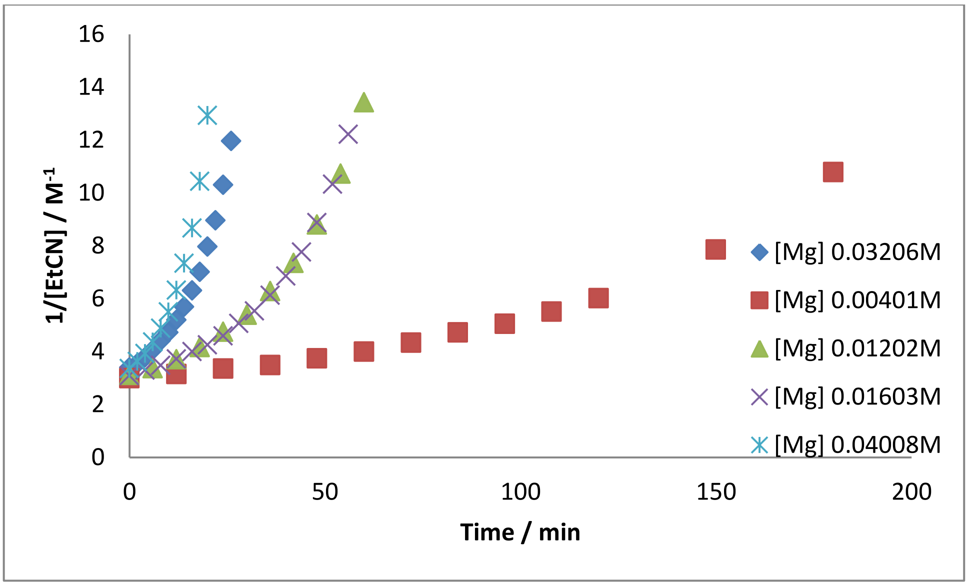

Figure S4. [Mg] vs k_{obs} (from Fig. S1); shows first order dependence

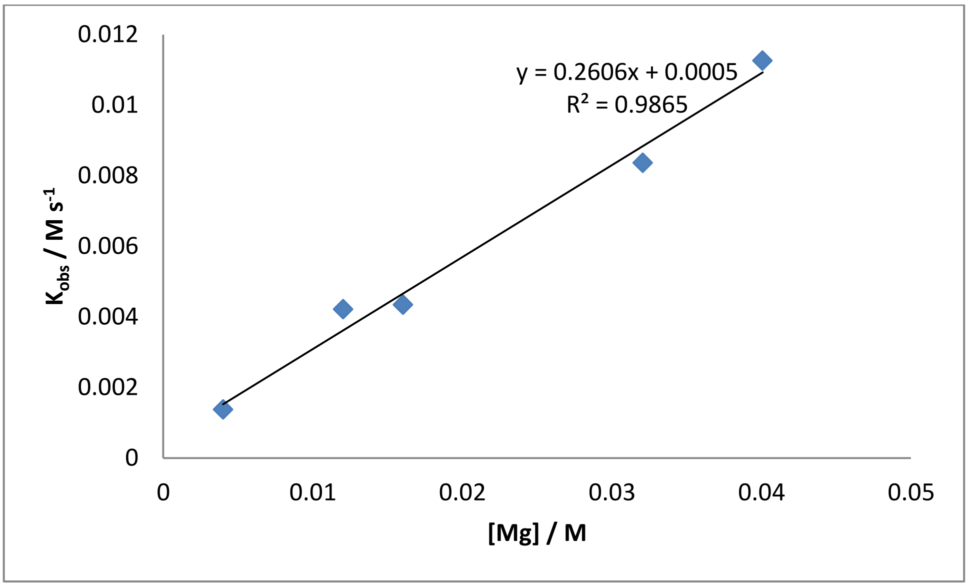

	Value	Error
m_1	0.000477	0.000435
m_2	0.260600	0.017616
Chisq	0.018455	n/a
R^2	0.9865	n/a

Determination of reaction order with respect to [HBpin]

Varying concentrations of starting reagent HBpin whilst keeping constant [Mg] = 0.04 M and pseudo first order conditions in EtCN (8.0 M).

Figure S5. [$EtCH_2N(Bpin)_2$] vs time for varying [HBpin]

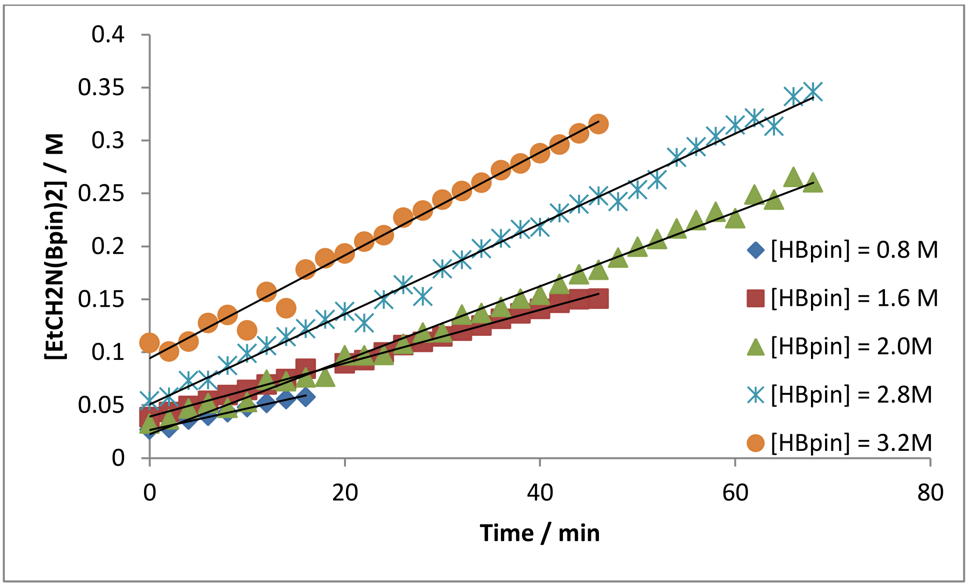

	[HBpin] = 0.8M	
	Value	Error
m_1	0.027214	0.001114
m_2	0.001908	0.000104
Chisq	0.00267759	n/a
R^2	0.976633	n/a

	[HBpin] = 1.6M	
	Value	Error
m_1	0.038840	0.000530
m_2	0.002547	0.000020
Chisq	8.696E-05	n/a
R^2	0.998720	n/a

	[HBpin] = 2.0M	
	Value	Error
m_1	0.022683	0.002139
m_2	0.003491	0.000054
Chisq	0.0192224	n/a
R^2	0.992143	n/a

	[HBpin] = 2.8M	
	Value	Error
m_1	0.050577	0.002271
m_2	0.004265	0.000057
Chisq	0.0013212	n/a
R^2	0.994052	n/a

	[HBpin] = 3.2M	
	Value	Error
m_1	0.094123	0.003163
m_2	0.004866	0.000118
Chisq	0.044993949	n/a
R^2	0.987265	n/a

Figure S6. ln([EtCH$_2$N(Bpin)$_2$]$_0$/[EtCH$_2$N(Bpin)$_2$]$_t$) vs time; non-linear kinetics

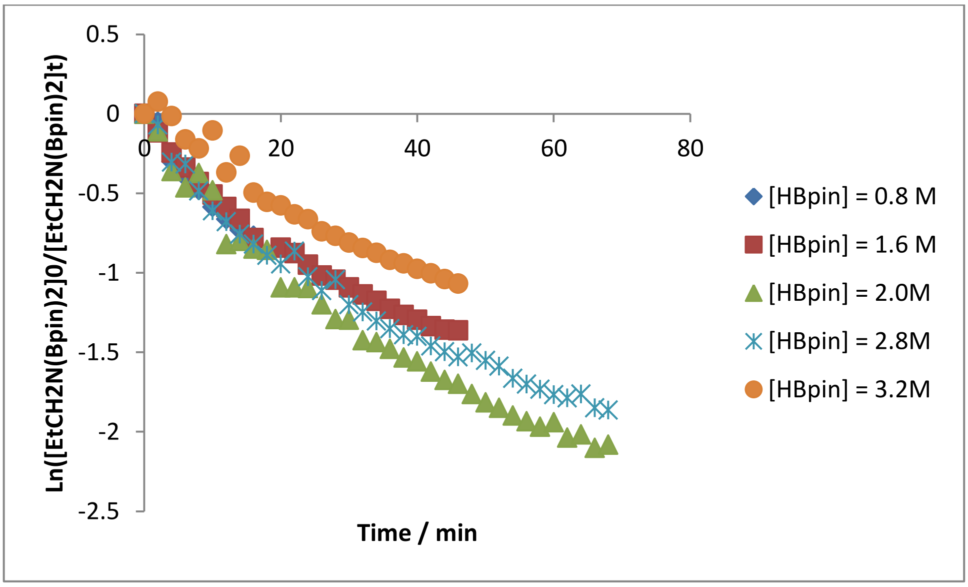

Figure S7. 1/[$EtCH_2N(Bpin)_2$] vs time; non-linear kinetics

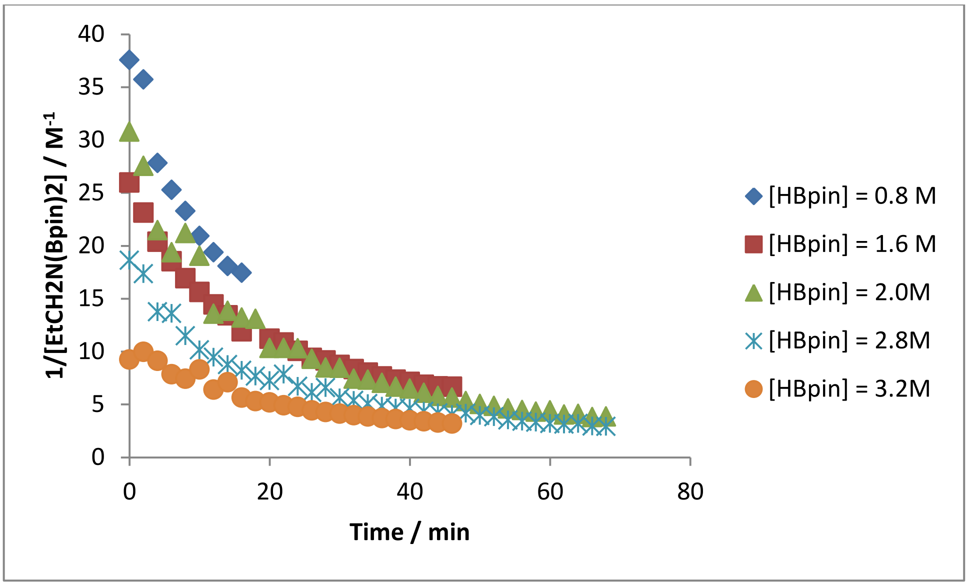

Figure S8. [HBpin] vs k_{obs}; linear fit indicates 1st order dependence on [HBpin]

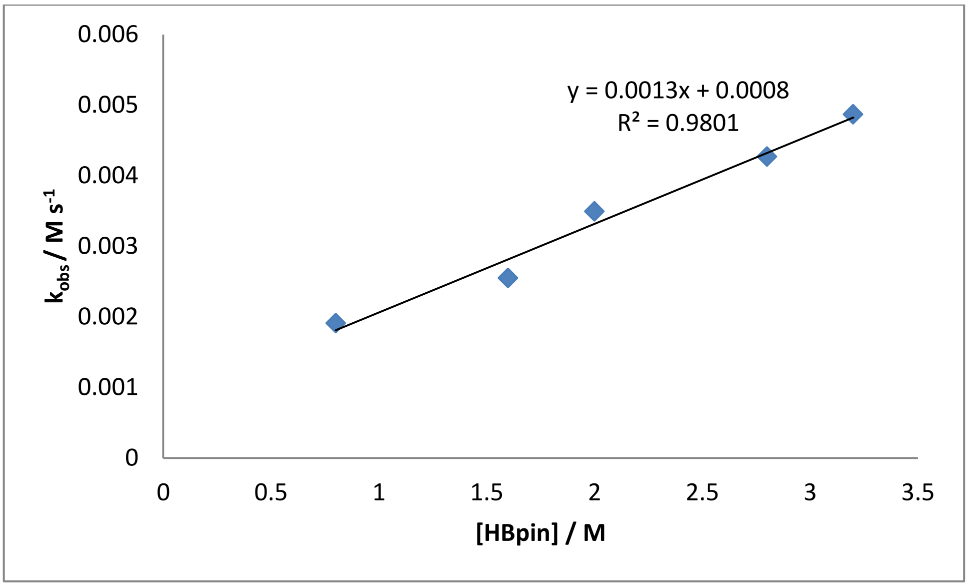

	Value	Error
m_1	0.000806	0.000232
m_2	0.001255	0.000103
Chisq	0.008197128	n/a
R^2	0.980118	n/a

Determination of reaction order with respect to [EtCN]

Varying concentrations of starting reagent EtCN whilst keeping constant [Mg] = 0.04M and pseudo first order conditions of HBpin (8.0M).

Figure S9. [$EtCH_2N(Bpin)_2$] vs time; variable [EtCN]

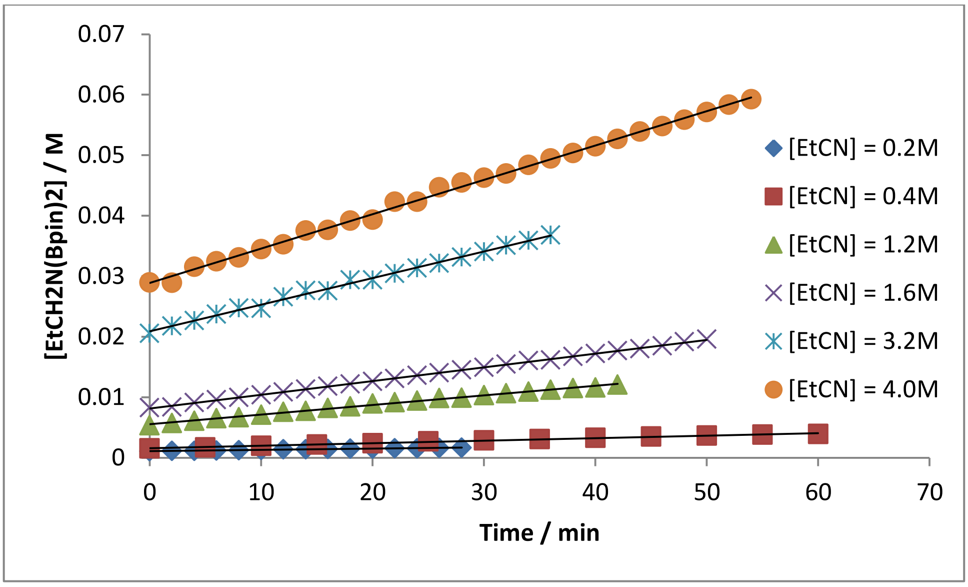

	[EtCN] = 0.2M	
	Value	Error
m_1	0.001066	0.000012
m_2	0.000021	0.000001
Chisq	0.009468	n/a
R^2	0.984067	n/a

	[EtCN] = 0.4M	
	Value	Error
m_1	0.001572	0.000035
m_2	0.000041	0.000001
Chisq	0.005337	n/a
R^2	0.993577	n/a

	[EtCN] = 1.2M	
	Value	Error
m_1	0.005545	0.000057
m_2	0.000158	0.000002
Chisq	0.009731	n/a
R^2	0.995669	n/a

	[EtCN] = 1.6M	
	Value	Error
m_1	0.008134	0.000038
m_2	0.000227	0.000001
Chisq	0.000912	n/a
R^2	0.999219	n/a

	[EtCN] = 3.2M	
	Value	Error
m_1	0.020904	0.000144
m_2	0.000440	0.000007
Chisq	0.00585	n/a
R^2	0.995901	n/a

	[EtCN] = 4.0M	
	Value	Error
m_1	0.028883	0.000179
m_2	0.000568	0.000006
Chisq	7.38E-05	n/a
R^2	0.997399	n/a

Figure S10. ln([EtCH$_2$N(Bpin)$_2$]$_0$/[EtCH$_2$N(Bpin)$_2$]$_t$) vs time; non-linear kinetics

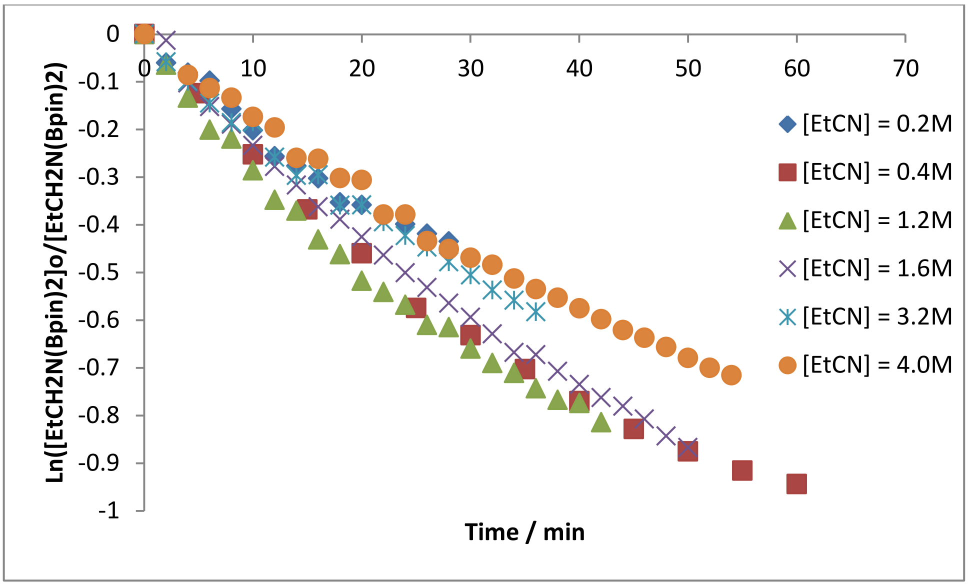

Figure S11. 1/[EtCH$_2$N(Bpin)$_2$] vs time; non-linear kinetics

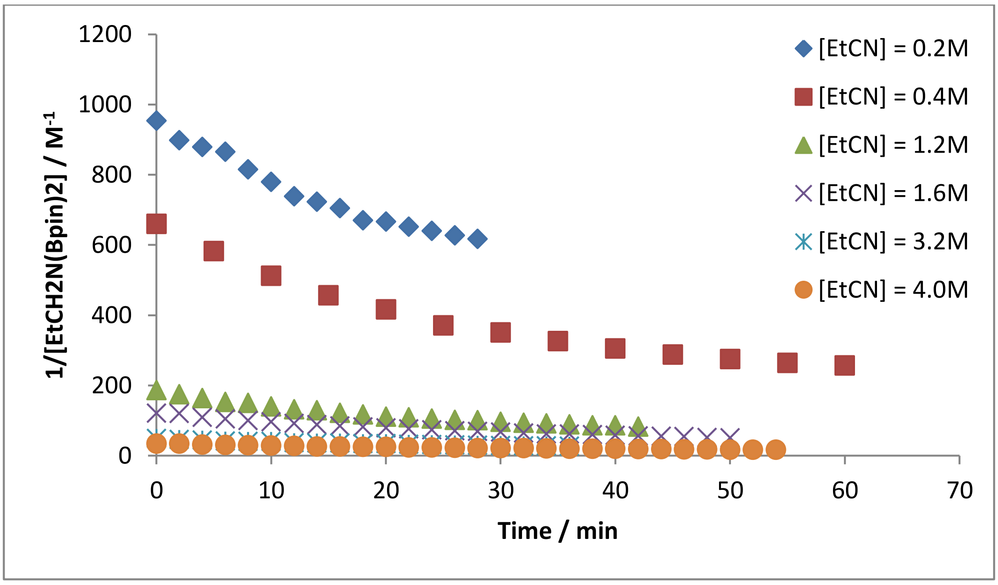

Figure S12. [EtCN] vs k_{obs}; indicates a first order dependence on [EtCN] under *pseudo*-first order conditions in HBpin

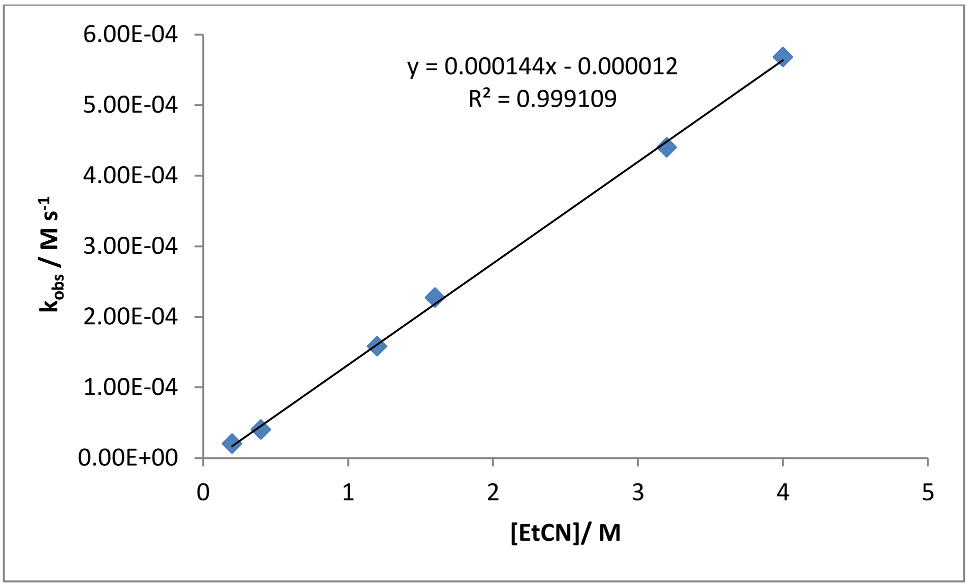

	Value	Error
m_1	-0.000012	0.000005
m_2	0.000144	0.000002
Chisq	0.000253	n/a
R^2	0.999109	n/a

Variable [Mg] under *pseudo*-first order in [HBpin]

Figure S13. [$EtCH_2N(Bpin)_2$] vs time; variation in [Mg] whilst under *pseudo*-first order conditions in [HBpin] = 8.0M and keeping [EtCN] = 0.4M constant

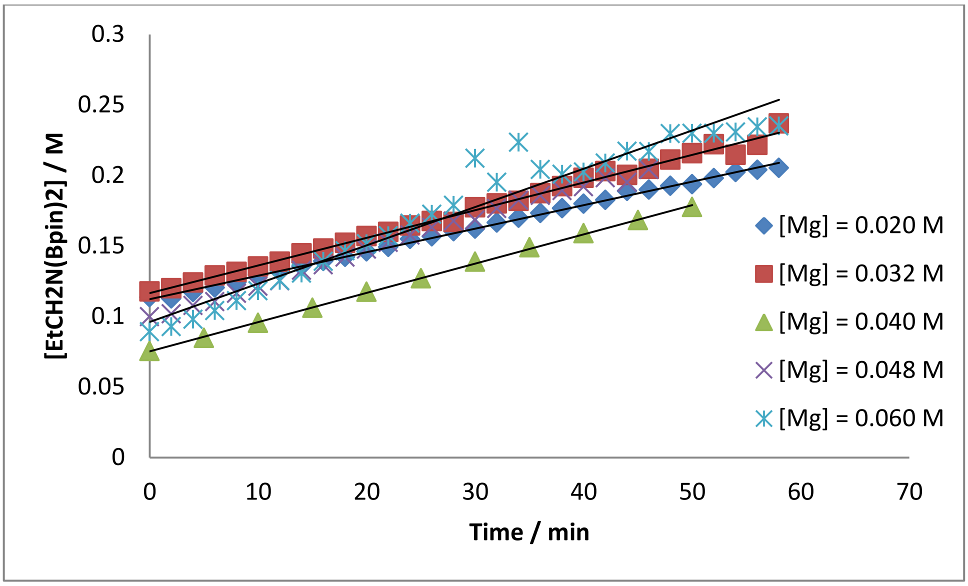

	[Mg] = 0.020 M	
	Value	Error
m_1	0.112321	0.000556
m_2	0.001664	0.000016
Chisq	0.001359	n/a
R^2	0.997272	n/a

	[Mg] = 0.032 M	
	Value	Error
m_1	0.108293	0.001005
m_2	0.001914	0.000030
Chisq	0.001194	n/a
R^2	0.993732	n/a

	[Mg] = 0.040 M	
	Value	Error
m_1	0.075126	0.000482
m_2	0.002078	0.000016
Chisq	1.94E-05	n/a
R^2	0.999448	n/a

	[Mg] = 0.048 M	
	Value	Error
m_1	0.098833	0.000985
m_2	0.002352	0.000037
Chisq	0.000583	n/a
R^2	0.994673	n/a

	[Mg] = 0.060 M	
	Value	Error
m_1	0.090021	0.001977
m_2	0.002954	0.000075
Chisq	0.000654	n/a
R^2	0.943791	n/a

Figure S14. ln($[EtCH_2N(Bpin)_2]_0$/$[EtCH_2N(Bpin)_2]_t$) vs time; non-linear kinetics

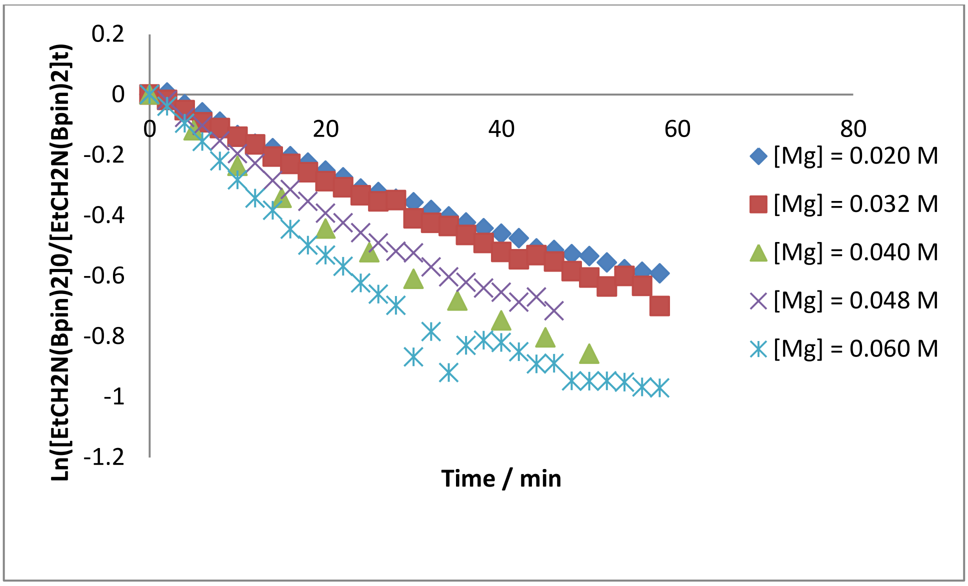

Figure S15. 1/$[EtCH_2N(Bpin)_2]$ vs time; non-linear kinetics

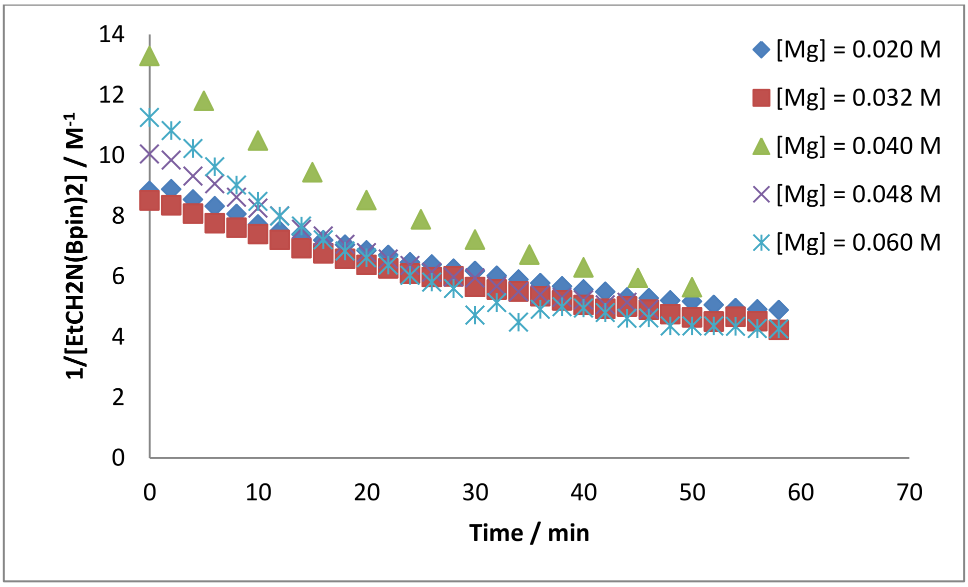

Figure S16. [Mg] vs k_{obs}; non-linear

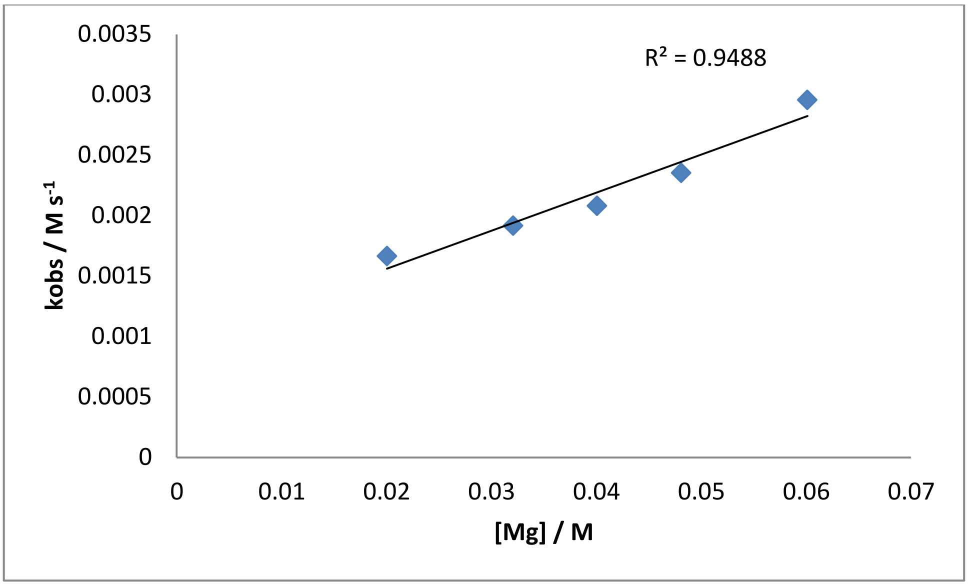

Figure S17. $[Mg]^2$ vs k_{obs}

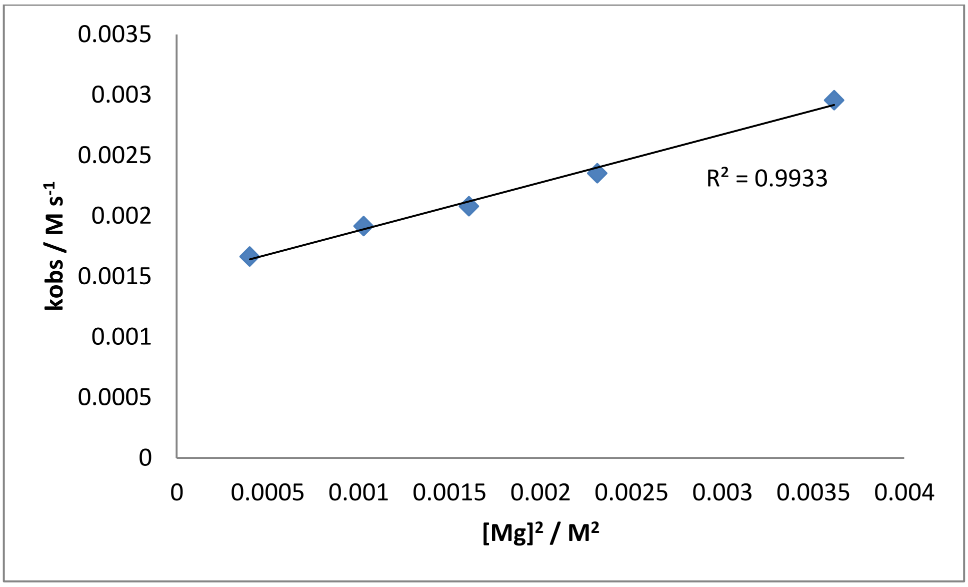

	Value	Error
m_1	0.001481	0.000040
m_2	0.397084	0.018813
Chisq	0.002838	n/a
R^2	0.993311	n/a

Figure S18. $[Mg]^3$ vs k_{obs}

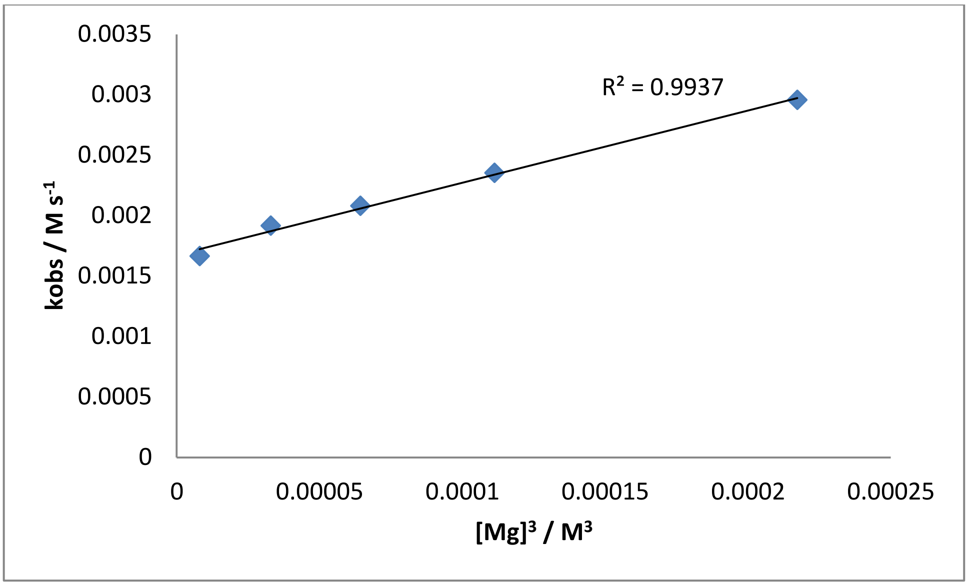

	Value	Error
m_1	0.001675	0.000031
m_2	5.965861	0.273449
Chisq	0.017854	n/a
R^2	0.993737	n/a

Variable [Mg] under *pseudo*-first order in [EtCN]

Figure S19. $[EtCH_2N(Bpin)_2]$ vs time; variable [Mg] under *pseudo*-first order in [EtCN] (4.0M) whilst keeping [HBpin] (0.8 M) invariant

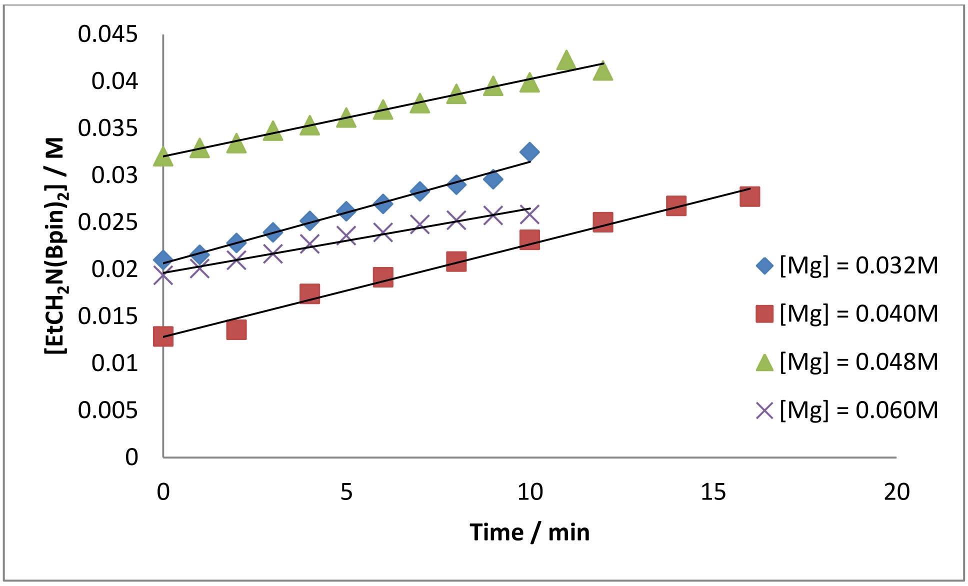

	[Mg] = 0.032M	
	Value	Error
m_1	0.020662	0.000261
m_2	0.001077	0.000044
Chisq	0.007186	n/a
R^2	0.985102	n/a

	[Mg] = 0.040M	
	Value	Error
m_1	0.012806	0.000411
m_2	0.000985	0.000043
Chisq	3.5E-05	n/a
R^2	0.986742	n/a

	[Mg] = 0.048M	
	Value	Error
m_1	0.032003	0.000238
m_2	0.000825	0.000034
Chisq	3.97E-05	n/a
R^2	0.981993	n/a

	[Mg] = 0.060M	
	Value	Error
m_1	0.019622	0.000198
m_2	0.000684	0.000034
Chisq	0.019062	n/a
R^2	0.978871	n/a

Figure S20. ln($[EtCH_2N(Bpin)_2]_0/[EtCH_2N(Bpin)_2]_t$) vs time; non-linear kinetics

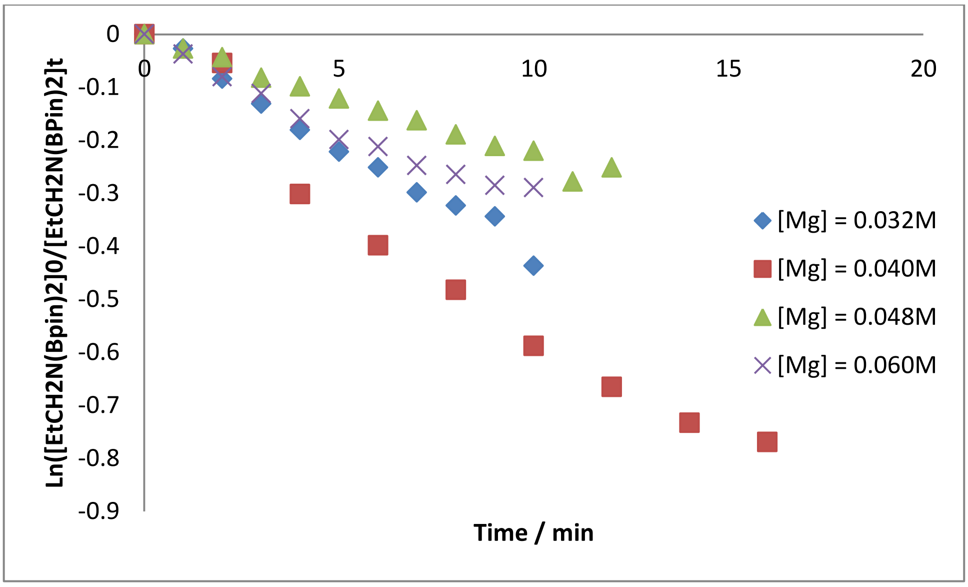

Figure S21. 1/$[EtCH_2N(Bpin)_2]$ vs time; non-linear kinetics

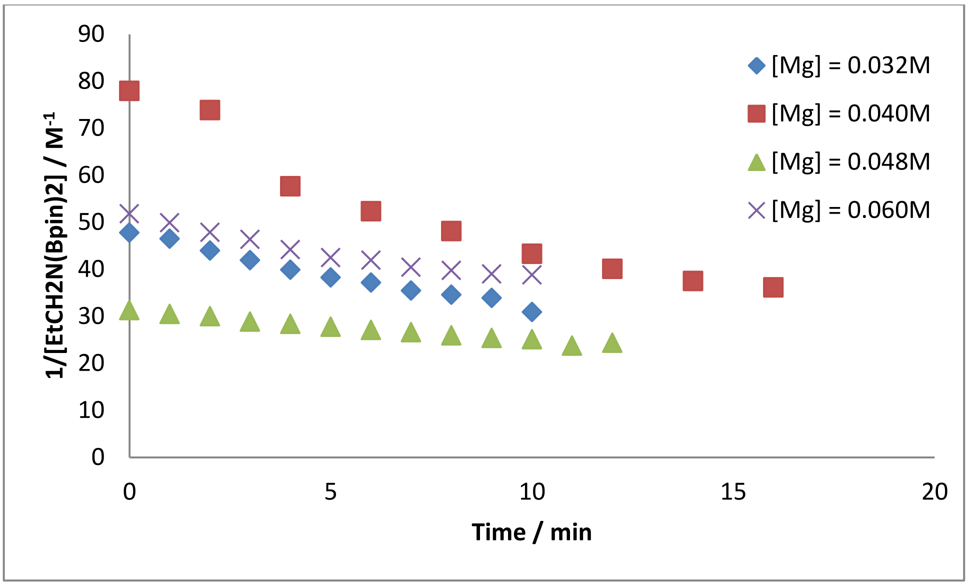

Figure S22. [Mg] vs k_{obs} ; indicates 1st order dependence on [Mg]

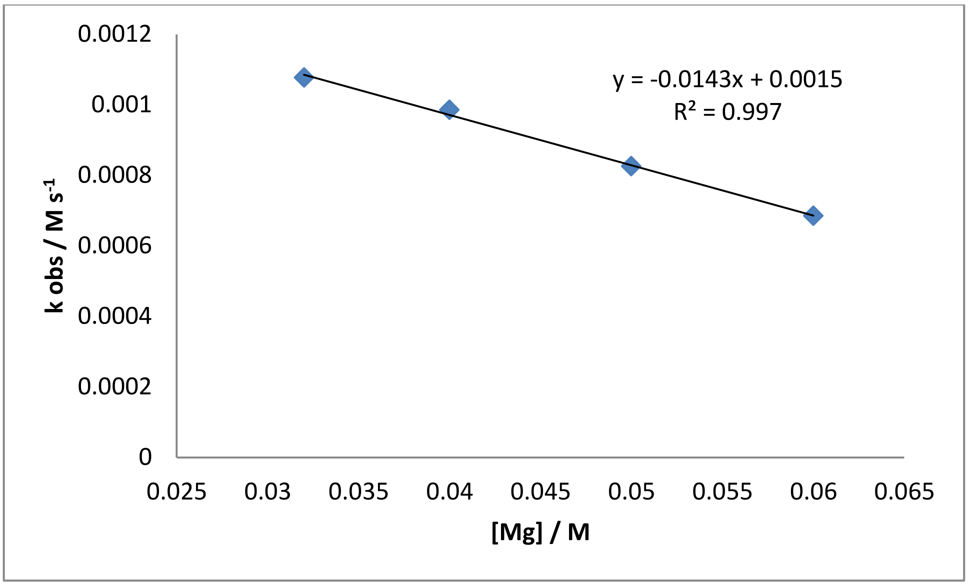

	Value	Error
m_1	0.001543	0.000026
m_2	-0.014281	0.000558
Chisq	0.003582	n/a
R^2	0.996957	n/a

Variable [EtCN] approaching 2:1 reaction stoichiometry

Figure S23. [$EtCH_2N(Bpin)_2$] vs time; variable [EtCN] whilst keeping [Mg] = 0.04M and [HBpin] = 0.84M

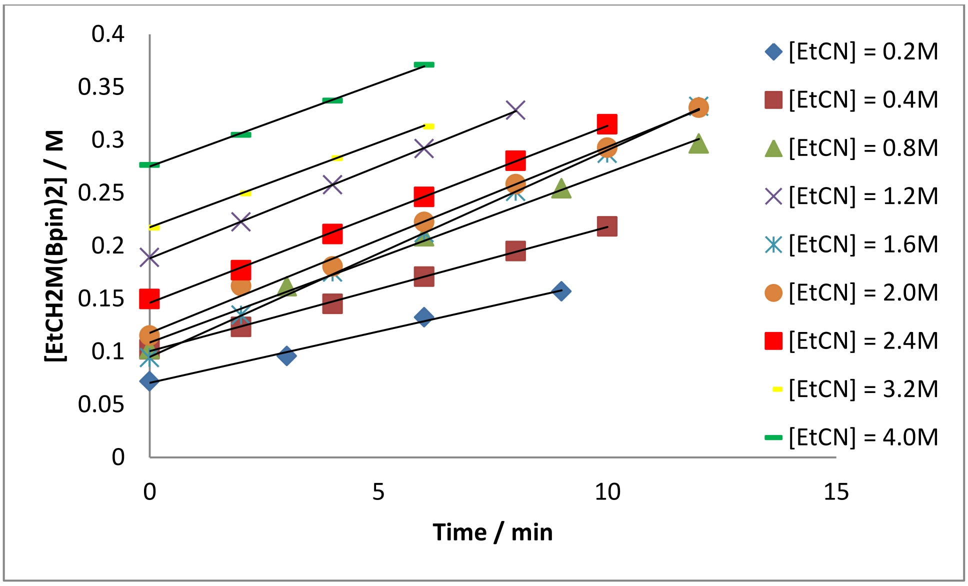

	[EtCN] = 0.2M	
	Value	Error
m_1	0.070346	0.003208
m_2	0.009723	0.000572
Chisq	0.00172655	n/a
R^2	0.993134	n/a

	[EtCN] = 0.4M	
	Value	Error
m_1	0.101748	0.001263
m_2	0.011449	0.000118
Chisq	2.398E-06	n/a
R^2	0.999147	n/a

	[EtCN] = 0.8M	
	Value	Error
m_1	0.108361	0.010890
m_2	0.016036	0.000680
Chisq	0.0031748	n/a
R^2	0.995617	n/a

	[EtCN] = 1.2M	
	Value	Error
m_1	0.188108	0.000651
m_2	0.017388	0.000133
Chisq	0.00023175	n/a
R^2	0.999825	n/a

	[EtCN] = 1.6M	
	Value	Error
m_1	0.094968	0.001308
m_2	0.019539	0.000181
Chisq	9.846E-05	n/a
R^2	0.999569	n/a

	[EtCN] = 2.0M	
	Value	Error
m_1	0.117474	0.003765
m_2	0.017587	0.000522
Chisq	0.0012035	n/a
R^2	0.995613	n/a

	[EtCN] = 2.4M	
	Value	Error
m_1	0.146617	0.001870
m_2	0.016518	0.000309
Chisq	0.00276225	n/a
R^2	0.997931	n/a

	[EtCN] = 3.2M	
	Value	Error
m_1	0.217298	0.000913
m_2	0.016029	0.000244
Chisq	7.272E-05	n/a
R^2	0.999536	n/a

	[EtCN] = 4.0M	
	Value	Error
m_1	0.274767	0.001674
m_2	0.015817	0.000448
Chisq	0.0018655	n/a
R^2	0.998402	n/a

Figure S24. ln([$EtCH_2N(Bpin)_2$]$_0$/[$EtCH_2N(Bpin)_2$]$_t$) vs time; non-linear kinetics

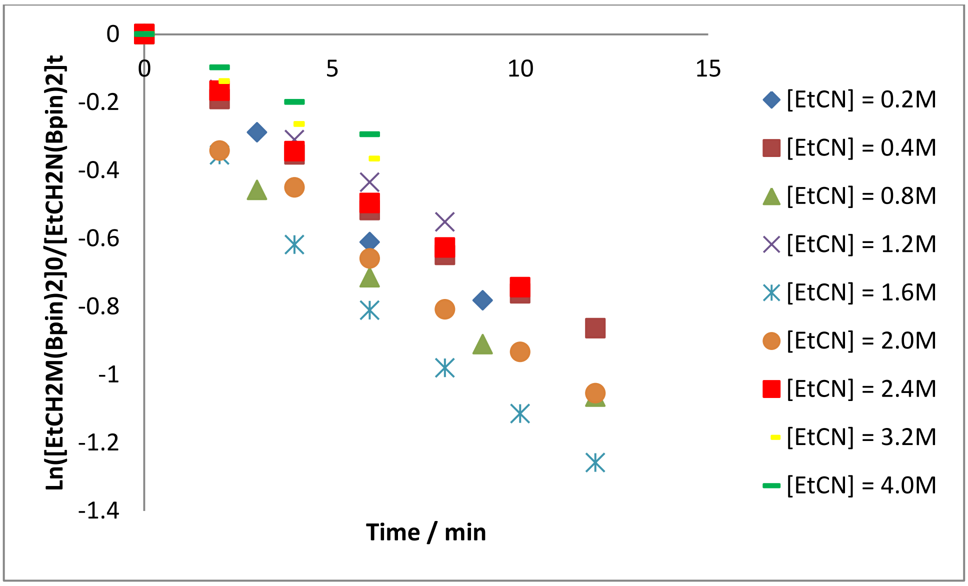

Figure S25. 1/[$EtCH_2N(Bpin)_2$] vs time; non-linear kinetics

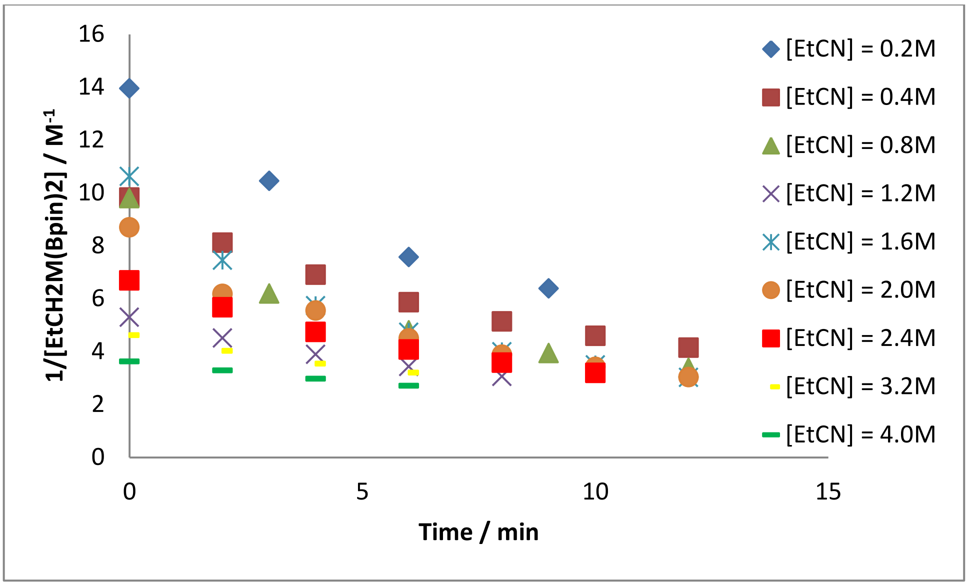

Figure S26. [EtCN] vs k_{obs}; indicating variable dependence on [EtCN] upon rate of reaction

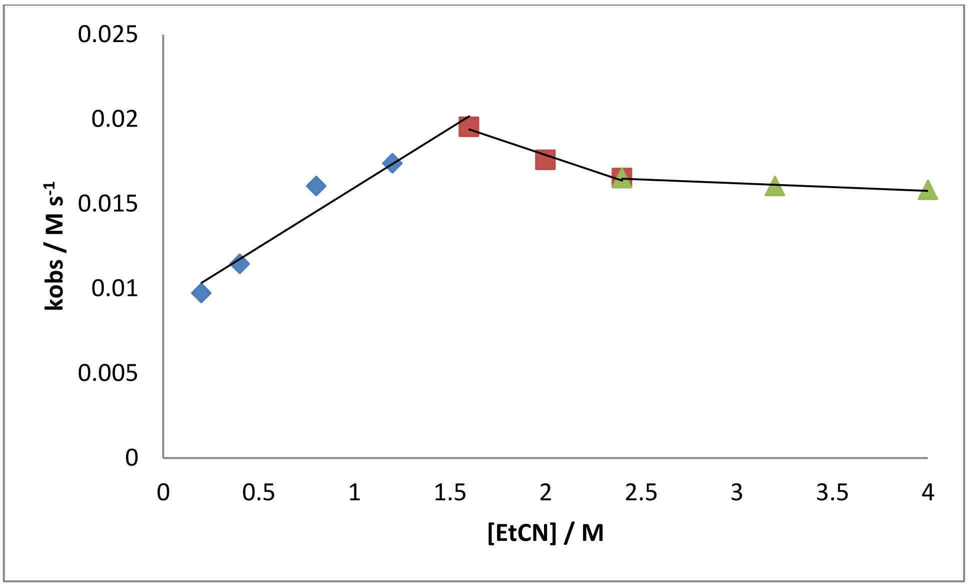

	[EtCN] = 0.2 – 1.6M	
	Value	Error
m_1	0.008930	0.000868
m_2	0.007020	0.000883
Chisq	0.02757781	n/a
R^2	0.95470	n/a

	[EtCN] = 1.6 – 2.4M	
	Value	Error
m_1	0.025434	0.001291
m_2	-0.003776	0.000637
Chisq	0.0137380	n/a
R^2	0.9592	n/a

	[EtCN] = 2.4 – 4.0M	
	Value	Error
m_1	0.017523	0.000326
m_2	-0.000438	0.000100
Chisq	0.0247721	n/a
R^2	0.9262	n/a

Variable [HBpin] approaching 2:1 reaction stoichiometry

Figure S27. [$EtCH_2N(Bpin)_2$] vs time for variable [HBpin] whilst keeping [Mg] = 0.04M and [EtCN] = 0.4M

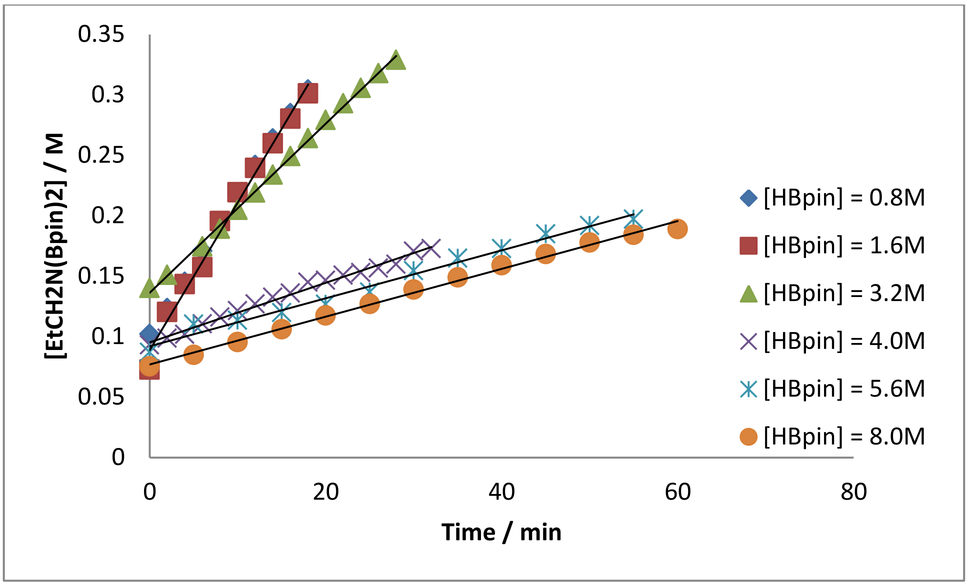

	[HBpin] = 0.8M	
	Value	Error
m_1	0.101748	0.001263
m_2	0.011449	0.000118
Chisq	2.3979E-06	n/a
R^2	0.999147	n/a

	[HBpin] = 1.6M	
	Value	Error
m_1	0.090523	0.004952
m_2	0.011946	0.000419
Chisq	0.0561306	n/a
R^2	0.989071	n/a

	[HBpin] = 3.2M	
	Value	Error
m_1	0.136191	0.001343
m_2	0.007001	0.000079
Chisq	0.0039901	n/a
R^2	0.998473	n/a

	[HBpin] = 4.0M	
	Value	Error
m_1	0.095307	0.098612
m_2	0.002462	0.000767
Chisq	7.5365E-06	n/a
R^2	0.989688	n/a

	[HBpin] = 5.6M	
	Value	Error
m_1	0.091912	0.002417
m_2	0.001987	0.000074
Chisq	0.0205431	n/a
R^2	0.986156	n/a

	[HBpin] = 8.0M	
	Value	Error
m_1	0.076936	0.001491
m_2	0.001976	0.000042
Chisq	0.0020196	n/a
R^2	0.995011	n/a

Figure S28. ln([EtCH$_2$N(Bpin)$_2$]$_0$/[EtCH$_2$N(Bpin)$_2$]$_t$) vs time; non-linear kinetics

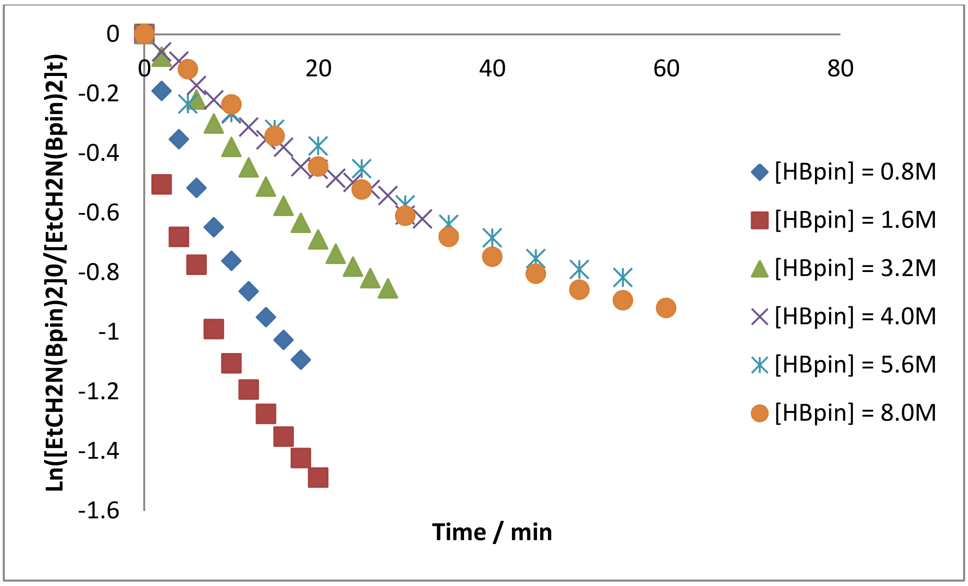

Figure S29. 1/[EtCH$_2$N(Bpin)$_2$] vs time; non-linear kinetics

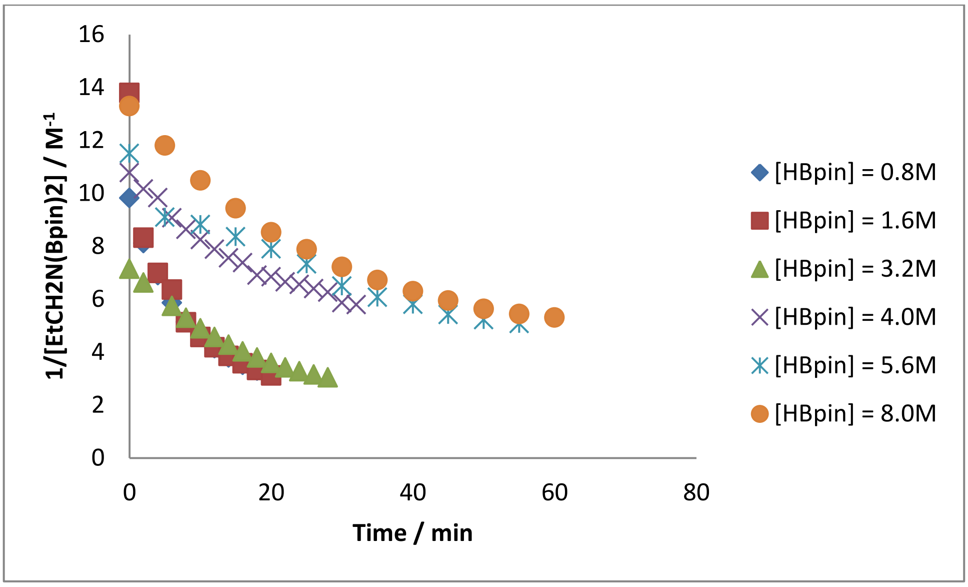

Figure S30. [HBpin] vs k_{obs}; indicates a variable dependence upon rate of reaction with changing [HBpin]

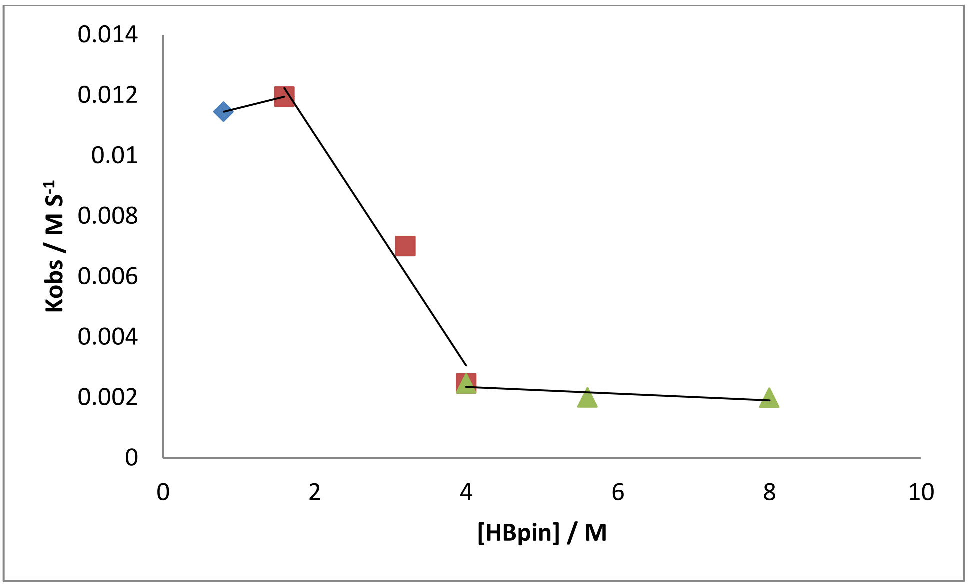

	[HBpin] = 1.6 – 4.0M	
	Value	Error
m_1	0.018367	0.001980
m_2	-0.003829	0.000639
Chisq	0.0057859	n/a
R^2	0.972887	n/a

	[HBpin] = 4.0-8.0M	
	Value	Error
m_1	0.002800	0.000487
m_2	-0.000112	0.000080
Chisq	0.2357439	n/a
R^2	0.99847	n/a

Variable Temperature Studies

Figure S31. [EtCN] vs time; variable temperature

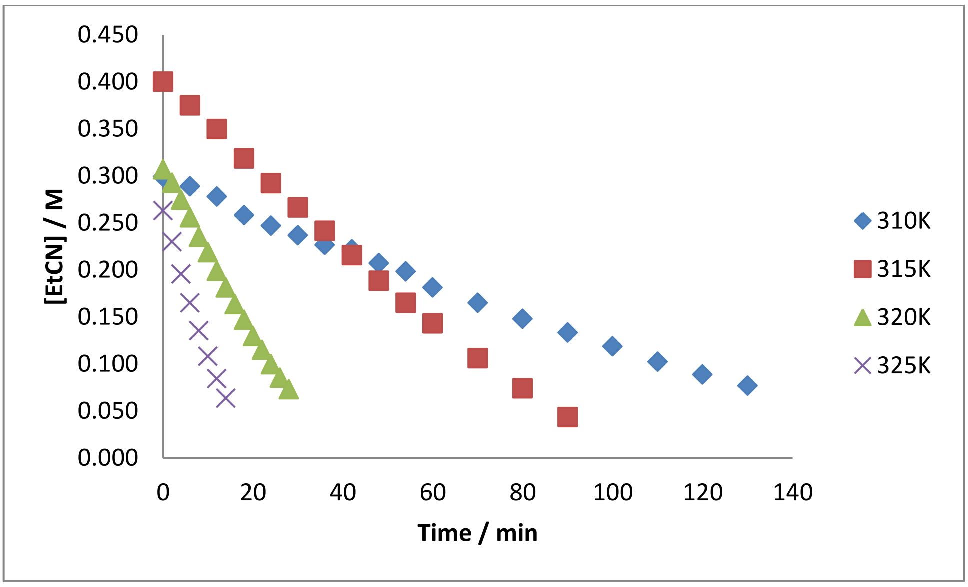

	T = 310 K	
	Value	Error
m_1	0.293231	0.006892
m_2	-0.001765	0.000097
Chisq	0.007775	n/a
R^2	0.995548	n/a

	T = 315 K	
	Value	Error
m_1	0.391690	0.003811
m_2	-0.004048	0.000078
Chisq	0.005843	n/a
R^2	0.995542	n/a

	T = 320 K	
	Value	Error
m_1	0.305263	0.001915
m_2	-0.008599	0.000116
Chisq	0.000411	n/a
R^2	0.997624	n/a

	T = 325 k	
	Value	Error
m_1	0.256095	0.003864
m_2	-0.014385	0.000462
Chisq	0.010242	n/a
R^2	0.993854	n/a

Figure S32. ln($[EtCN]_0/[EtCN]_t$) vs time; non-linear kinetics

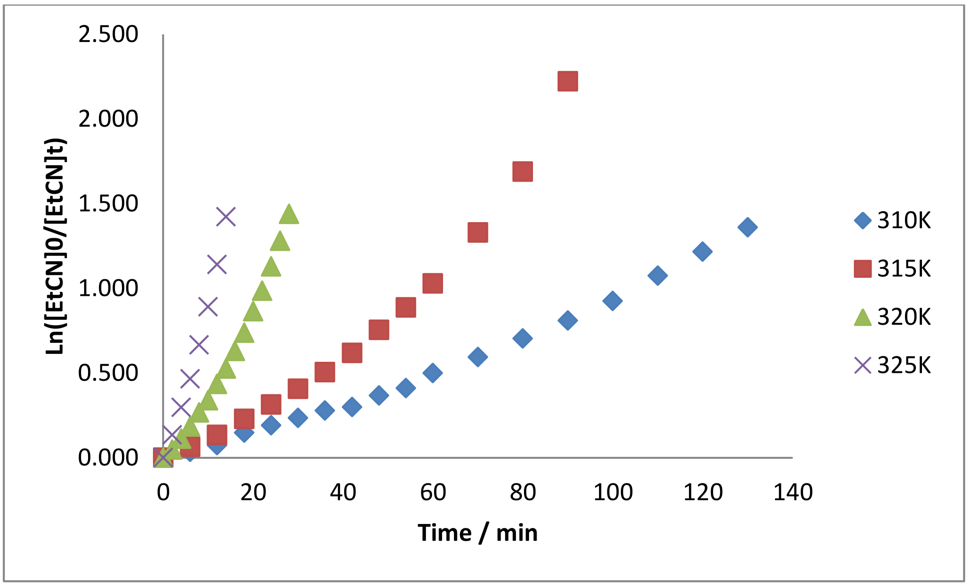

Figure S33. 1/[EtCN] vs time; non-linear kinetics

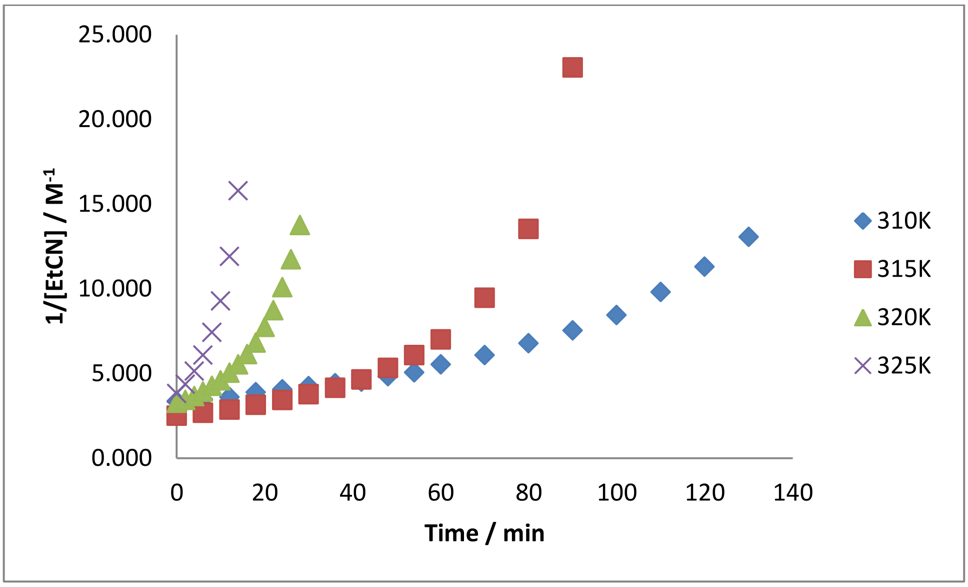

Figure S34. Eyring Plot – Ln(k/T) vs 1/T

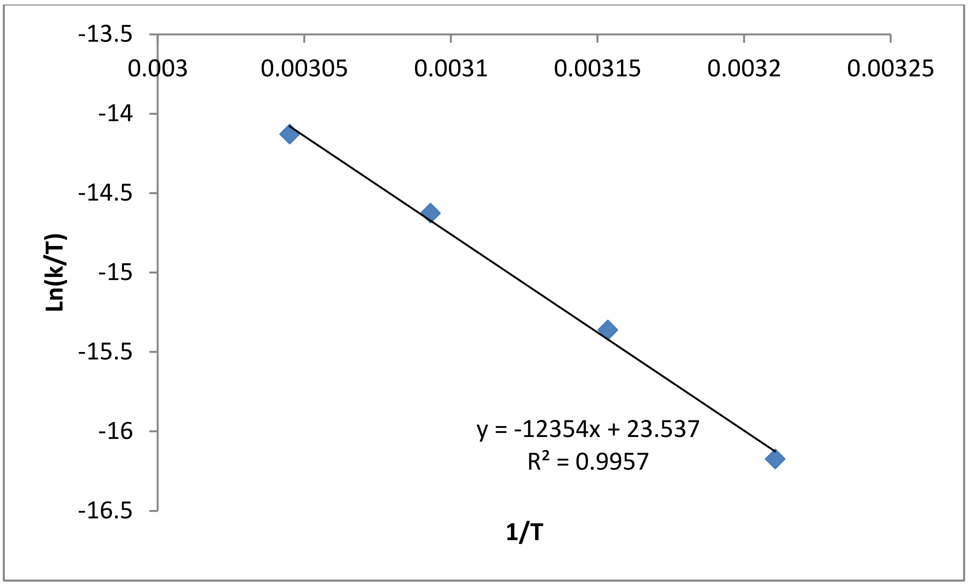

	Value	Error
m_1	23.537	1.790
m_2	-12354	572.60
Chisq	0.00398094	n/a
R^2	0.9957	n/a

This graph was used to calculate the following Activation Energy Parameters, least square error analysis was also carried to provide accurate error information.

	Value	Error
ΔH	102.71 kJ mol^{-1}	± 4.76
ΔS	-1.85 J k^{-1} mol^{-1}	± 14.88

Figure S35. Arrhenius Plot - Ln(k) vs 1/T

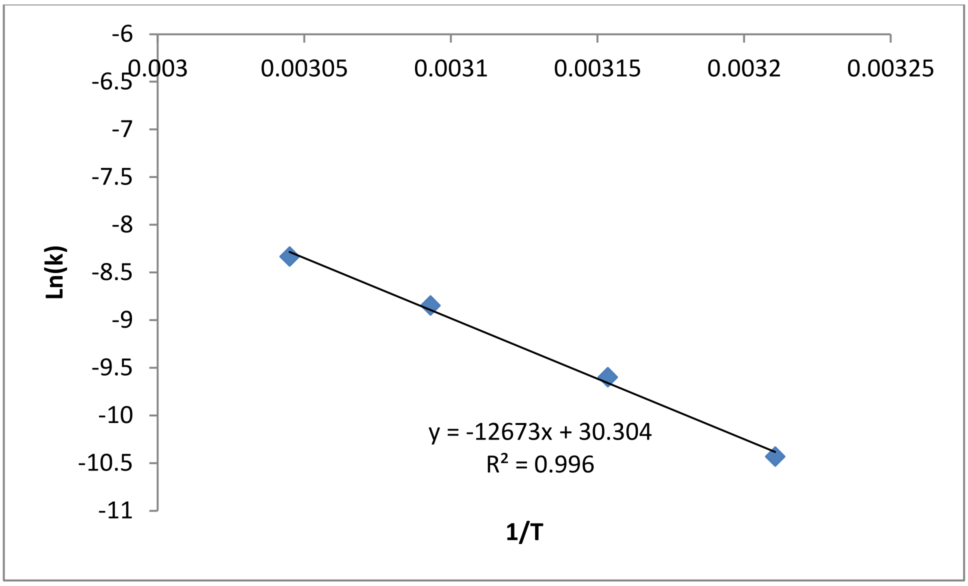

	Value	Error
m_1	30.304	1.785
m_2	-12673	570.876
Chisq	0.004023456	n/a
R^2	0.9960	n/a

This graph was used to calculate the following Activation Energy Parameter; least square error analysis was also carried to provide accurate error information.

	Value	Error
Ea	105.36 kJ mol^{-1}	± 4.75

Aryl Nitrile Kinetics

Hammett plot

Standard reaction used of 10 mg (0.02 mmol, ie. 10 mol%) of LMgBu was dissolved in 0.5 ml of C_6D_6, 60.9 µL (0.42 mmol) of pinacolborane was then added followed by 0.2 mmol of Nitrile. ^{1}H NMR spectra were collected at consistent intervals until reaction reached the desired 3 half-lives (80 % product conversion). Reaction was carried out with 8 different aryl substituted nitriles: 4-methoxybenzonitrile, para-tolunitrile, meta-tolunitrile, 3-methoxybenzonitrile, 3-Fluorobenzonitrile, 4-Chlorobenzonitrile, 4-(trifluoromethyl)benzonitrile, and benzonitrile. All reactions were carried out at 323 K.

Figure S36. [ArCN] vs time; non-linear kinetics

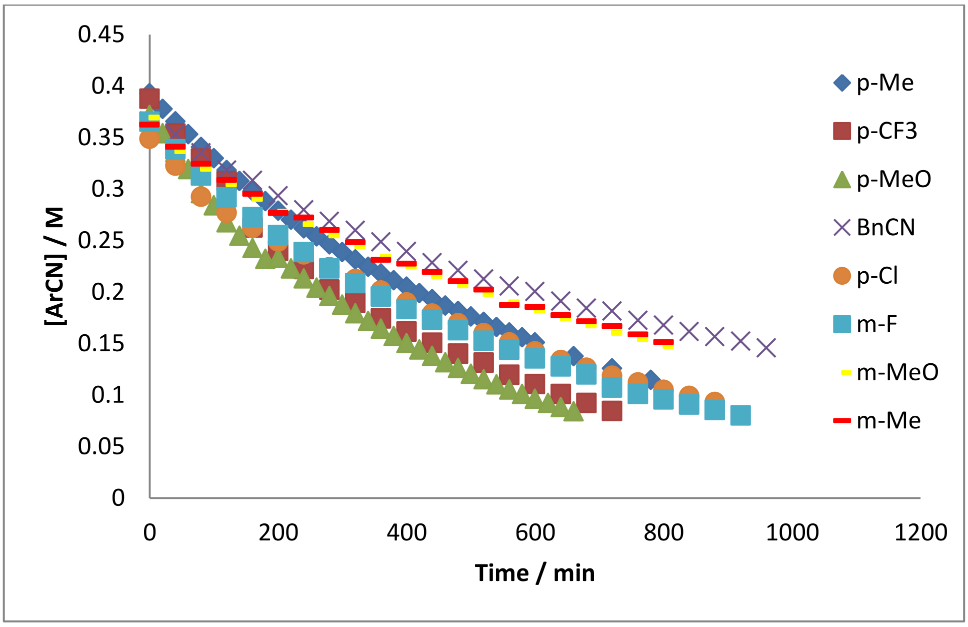

Figure S37. ln([ArCN]$_0$/[ArCN]$_t$) vs time for a series of different aryl nitriles

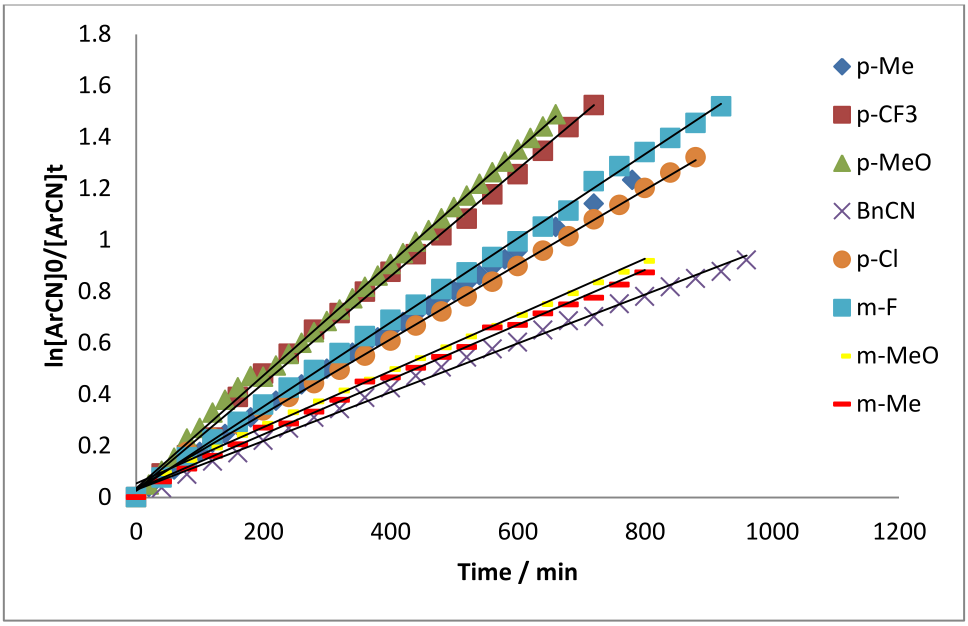

	p-Me	
	Value	Error
m_1	0.021810	0.002473
m_2	0.001560	0.000006
Chisq	0.004459	n/a
R^2	0.99949	n/a

	p-CF_3	
	Value	Error
m_1	0.027300	0.011683
m_2	0.002080	0.000028
Chisq	0.003583	n/a
R^2	0.99699	n/a

	p-MeO	
	Value	Error
m_1	0.035330	0.005828
m_2	0.002190	0.000015
Chisq	0.006753	n/a
R^2	0.99846	n/a

	BnCN	
	Value	Error
m_1	0.029425	0.006618
m_2	0.000949	0.000012
Chisq	0.011518	n/a
R^2	0.996445	n/a

	m-F	
	Value	Error
m_1	0.019040	0.002459
m_2	0.001360	0.000005
Chisq	0.002541	n/a
R^2	0.99975	n/a

	p-Cl	
	Value	Error
m_1	-0.097600	0.005398
m_2	0.048200	0.000011
Chisq	0.064175	n/a
R^2	0.99890	n/a

	m-MeO	
	Value	Error
m_1	0.054630	0.006236
m_2	0.001090	0.000013
Chisq	0.042573	n/a
R^2	0.99717	n/a

	m-Me	
	Value	Error
m_1	0.032360	0.006857
m_2	0.001060	0.000015
Chisq	0.015722	n/a
R^2	0.99640	n/a

Figure S38. 1/[ArCN] vs time; non-linear kinetics

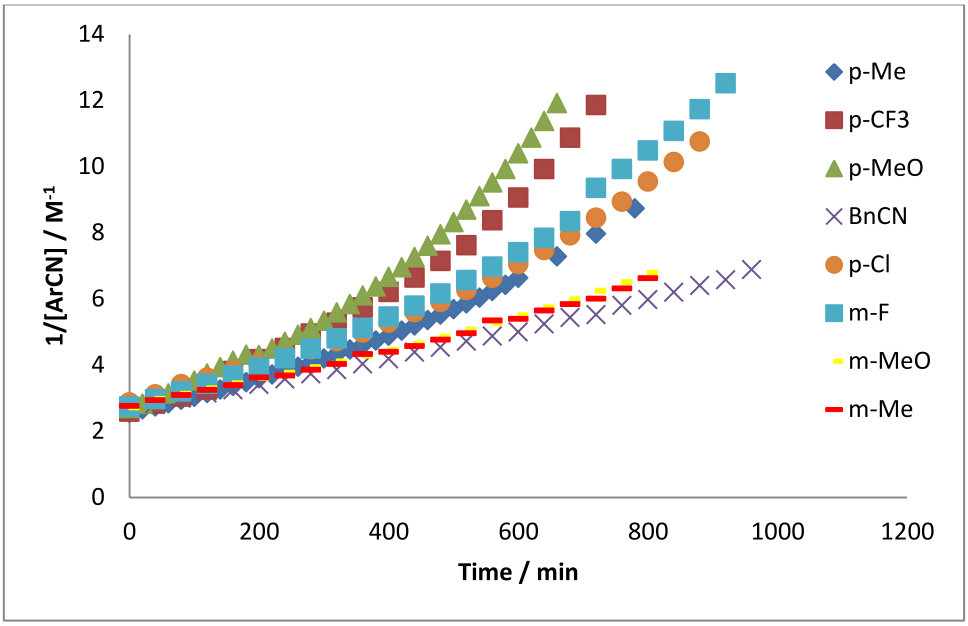

Figure S39. Hammett Plot; k_{obs} taken from 1st order plots

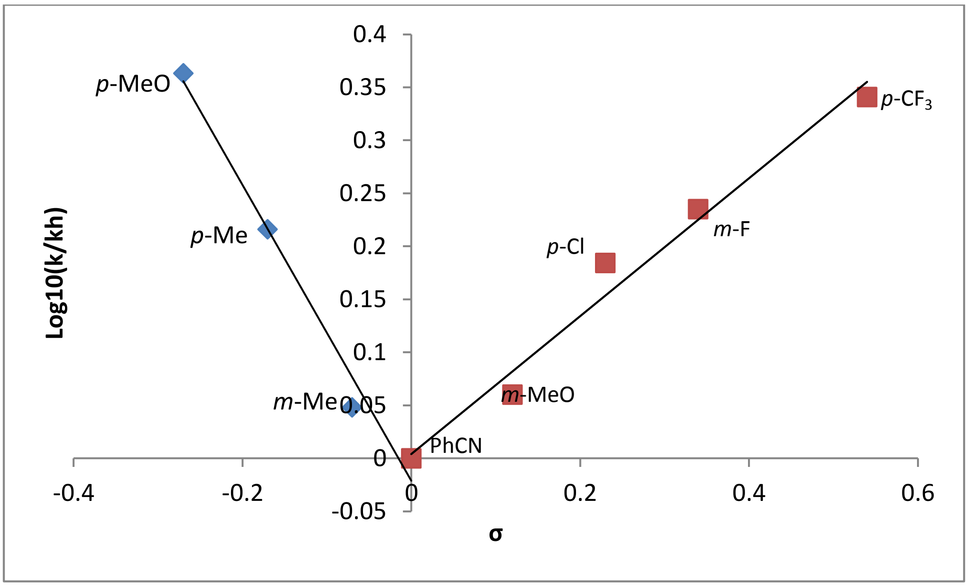

	Electron Donating Groups (EDG)	
	Value	Error
m_1	-0.021180	0.020524
m_2	-1.395670	0.125666
Chisq	0.021751	n/a
R^2	0.98404	n/a

	Electron Withdrawing Groups (EWG)	
	Value	Error
m_1	0.004090	0.017839
m_2	0.650020	0.057909
Chisq	0.001125	n/a
R^2	0.97674	n/a

	**ρ value**
**Electron donating group (EDG)**	-1.40
**Electron withdrawing group (EWG)**	+0.65

Electron Donating Aryl nitrile (*p*-$MeOC_6H_4CN$)

Determination of Catalyst order

Figure S40. [*p*-$MeOC_6H_4CN$] vs time; non-linear kinetics for 1:2 *p*-$MeOC_6H_4CN$:HBpin

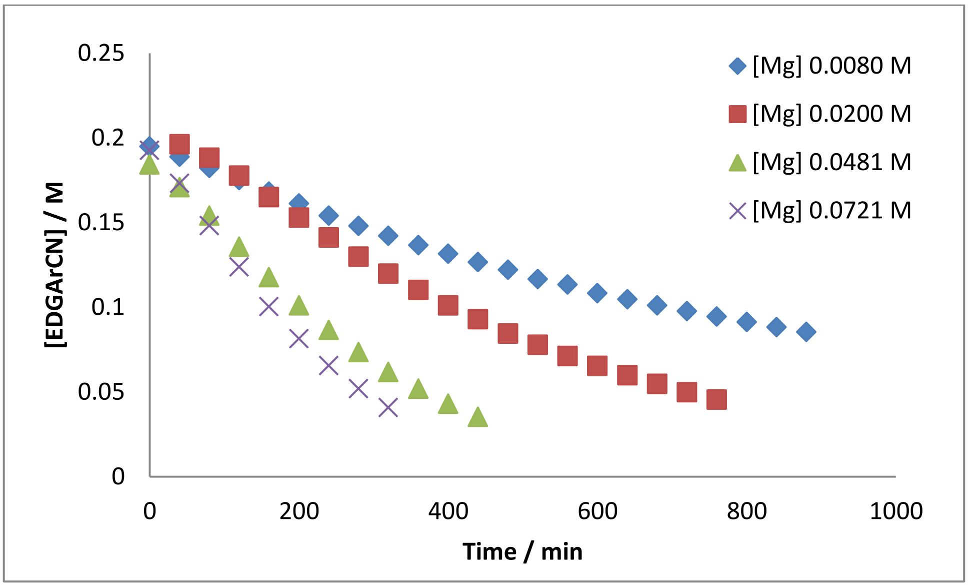

Figure S41. ln([*p*-$MeOC_6H_4CN]_0$/[p-$MeOC_6H_4CN]_t$) vs time for 1:2 *p*-$MeOC_6H_4CN$:HBpin

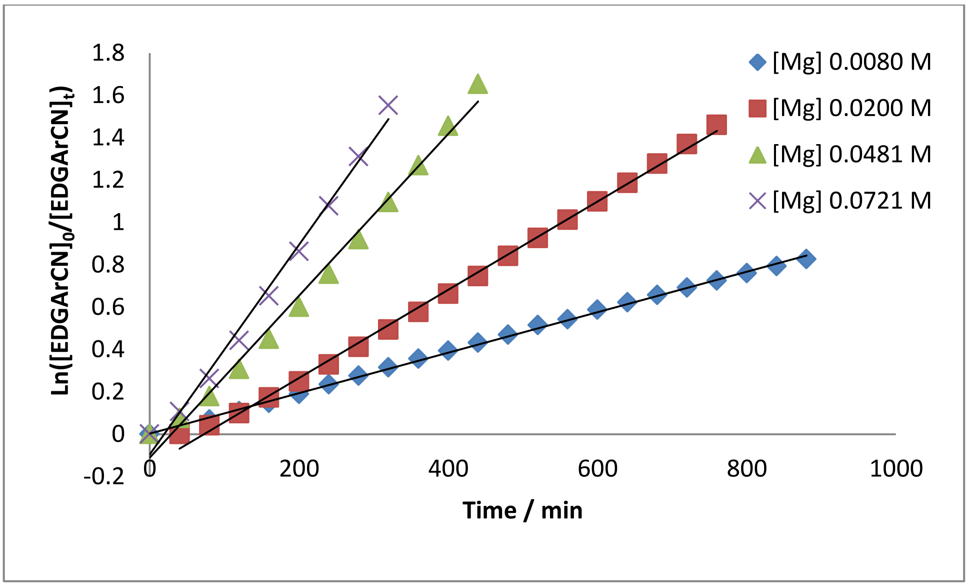

	[Mg] 0.00802 M	
	Value	Error
m_1	0.002229	0.003687
m_2	0.000957	0.000007
Chisq	7.7E-05	n/a
R^2	0.998821	n/a

	[Mg] 0.02004 M	
	Value	Error
m_1	-0.071470	0.010574
m_2	0.002100	0.000024
Chisq	0.021781	n/a
R^2	0.99769	n/a

	[Mg] 0.0481 M	
	Value	Error
m_1	-0.110970	0.031377
m_2	0.003820	0.000121
Chisq	0.043733	n/a
R^2	0.99012	n/a

	[Mg] 0.0721 M	
	Value	Error
m_1	-0.095350	0.034113
m_2	0.004950	0.000179
Chisq	0.034514	n/a
R^2	0.99090	n/a

Figure S42. 1/[*p*-$MeOC_6H_4CN$] vs time; non-linear kinetics for 1:2 *p*-$MeOC_6H_4CN$:HBpin

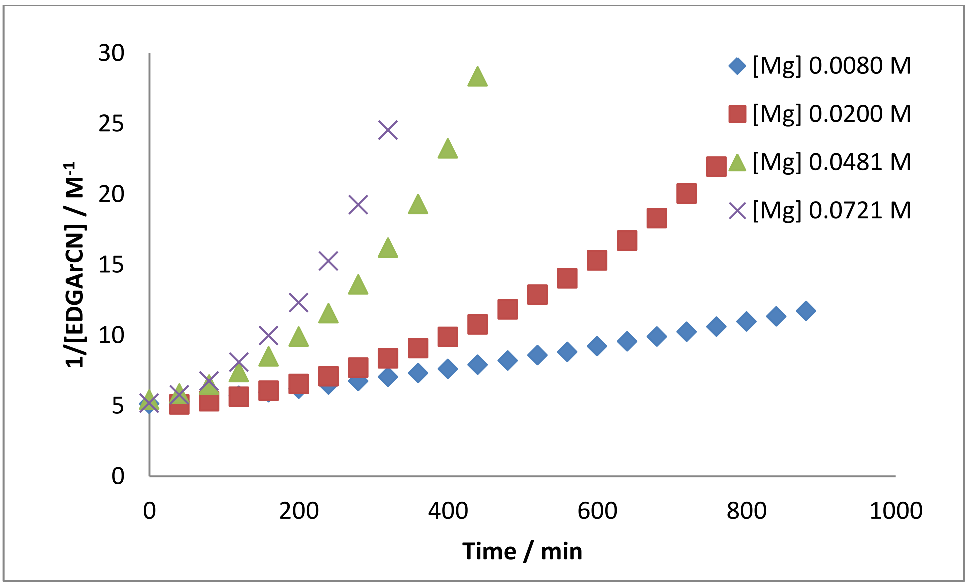

Figure S43. [Mg] vs k_{obs}; indicating a first order dependence on [Mg]

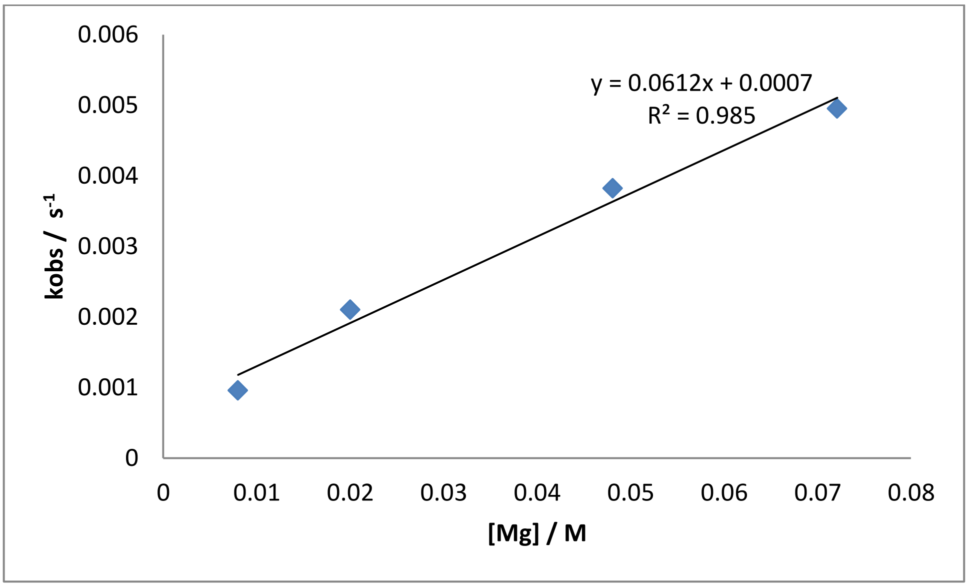

	Value	Error
m_1	0.000688	0.000239
m_2	0.061193	0.005354
Chisq	0.020769	n/a
R^2	0.984918	n/a

Determination of order in [p-MeOC$_6$H$_4$CN]

Figure S44. [p-MeOC$_6$H$_4$CH$_2$N(Bpin)$_2$] vs time; non-linear kinetics

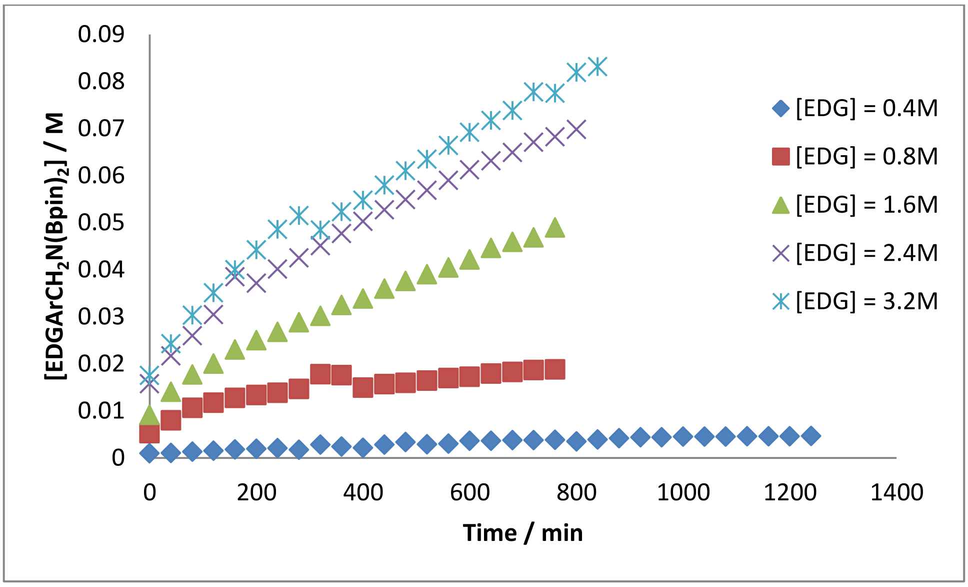

Figure S45. ln([p-MeOC$_6$H$_4$CH$_2$N(Bpin)$_2$]$_0$/[p-MeOC$_6$H$_4$CH$_2$N(Bpin)$_2$]$_t$) vs time; non-linear kinetics

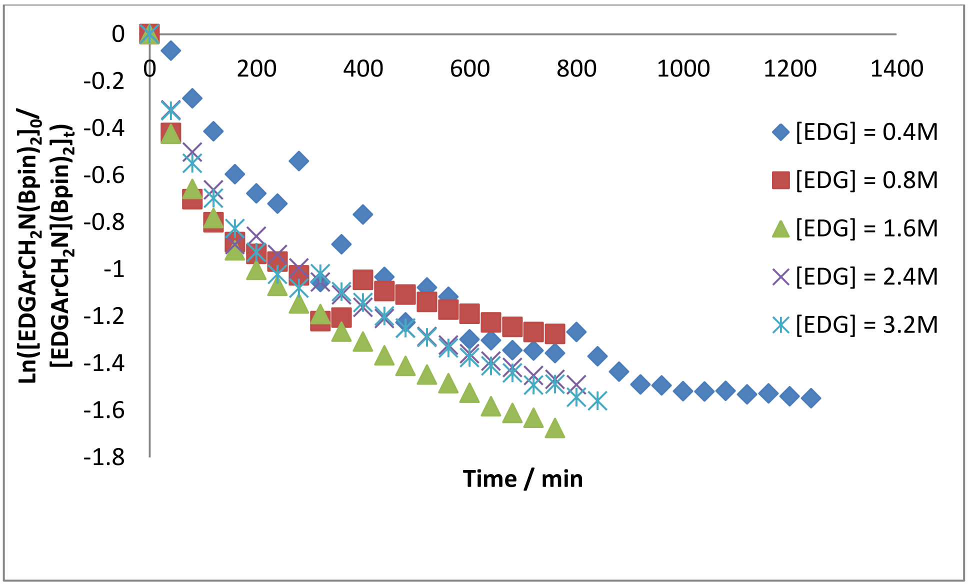

Figure S46. 1/[p-MeOC$_6$H$_4$CH$_2$N(Bpin)$_2$] vs time; non-linear kinetics

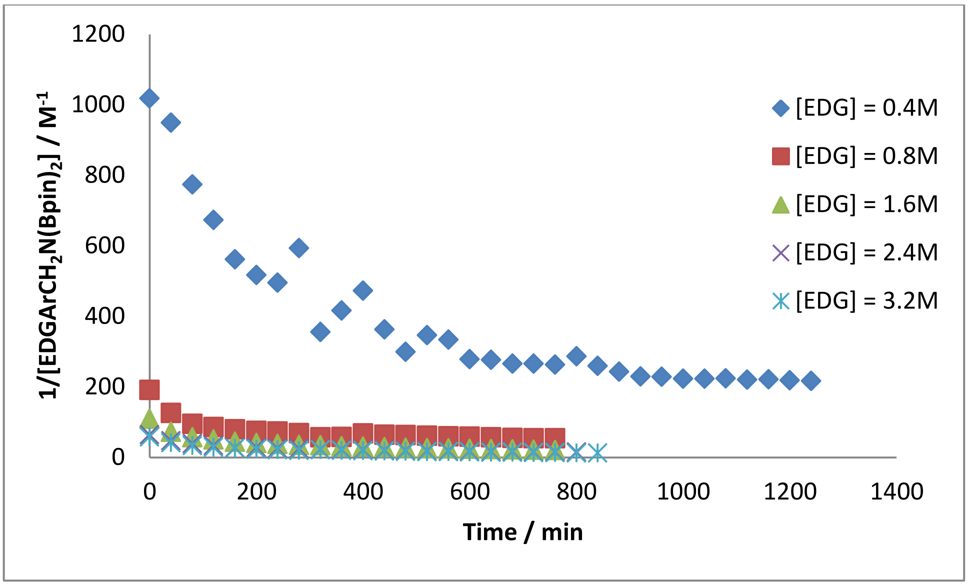

Figure S47. [p-MeOC$_6$H$_4$CH$_2$N(Bpin)$_2$]^2 vs time

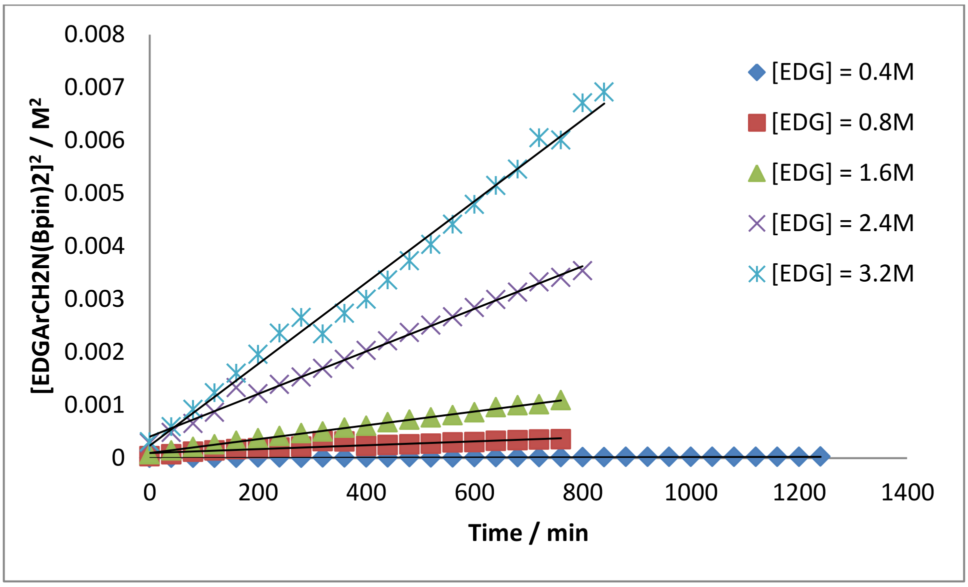

	[EDG] = 0.4M	
	Value	Error
m_1	8.0000E-08	4.89E-07
m_2	2.0000E-08	6.78E-10
Chisq	0.01609483	n/a
R^2	0.961347	n/a

	[EDG] = 0.8M	
	Value	Error
m_1	0.000102	3.018E-05
m_2	0.000000	6.790E-08
Chisq	0.746088	n/a
R^2	0.985122	n/a

	[EDG] = 1.6M	
	Value	Error
m_1	0.000094	6.287E-06
m_2	0.000001	1.414E-08
Chisq	0.007088	n/a
R^2	0.997887	n/a

	[EDG] = 2.4M	
	Value	Error
m_1	0.000401	3.360E-05
m_2	0.000004	7.185E-08
Chisq	0.01585	n/a
R^2	0.993990	n/a

	[EDG] = 3.2M	
	Value	Error
m_1	0.000235	8.774E-05
m_2	0.000008	1.788E-07
Chisq	0.001265	n/a
R^2	0.98930136	n/a

Figure S48. [*p*-$MeOC_6H_4CN$] vs k_{obs}; non-linear fit

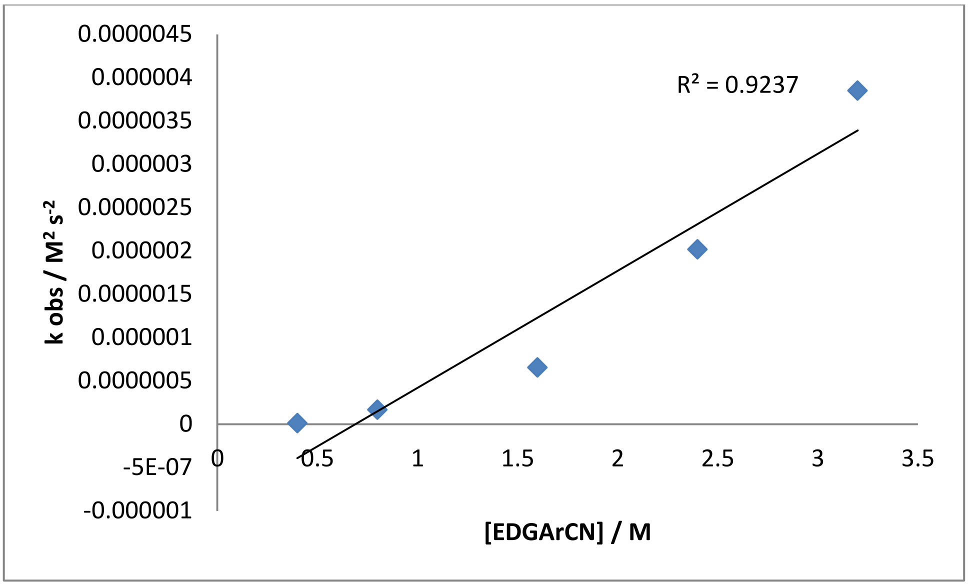

Figure S49. [*p*-$MeOC_6H_4CN$]$^{-1}$ vs k_{obs}; non-linear fit

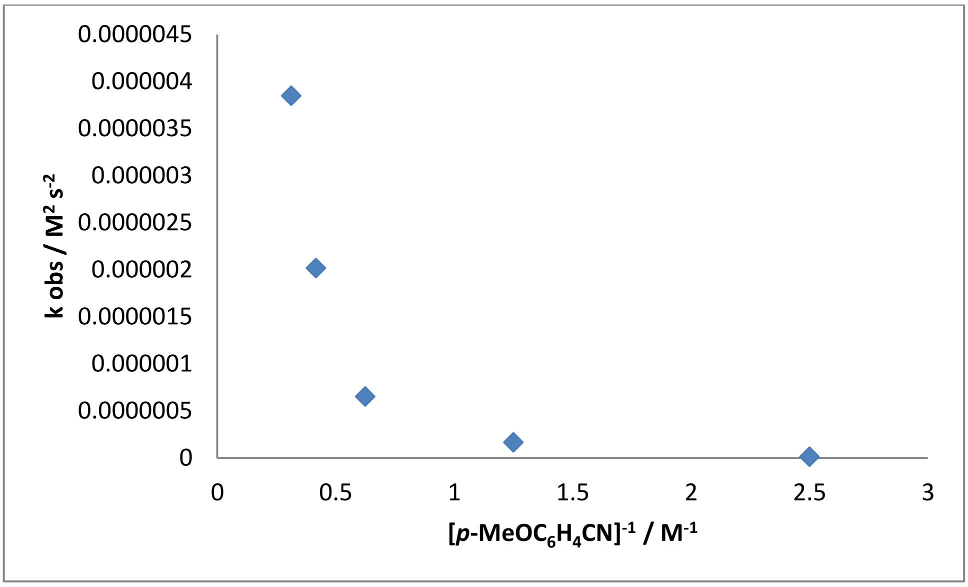

Figure S50. [*p*-MeOC$_6$H$_4$CN]$^{-1}$ vs k$_{obs}$

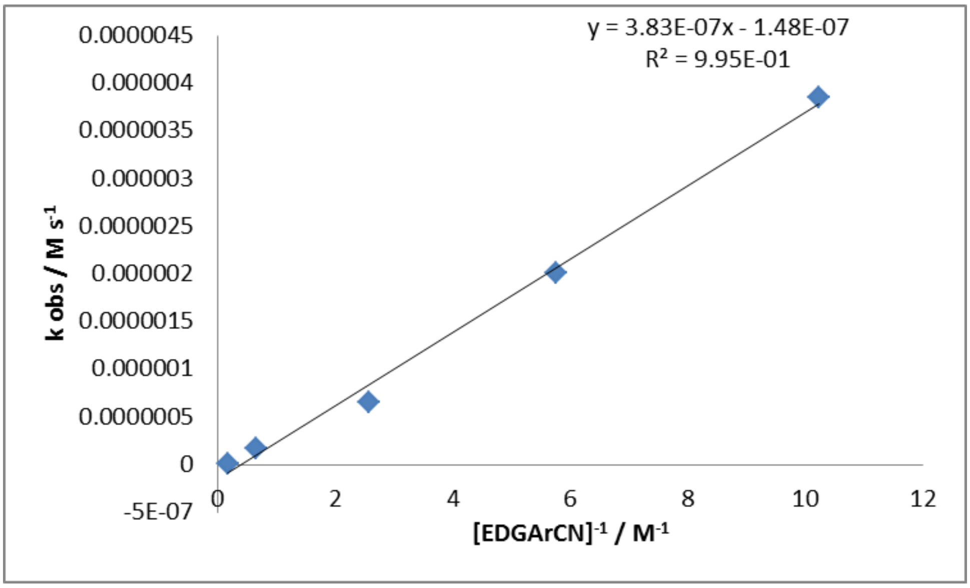

	Value	Error
m_1	-1.4800E-07	8.6431E-08
m_2	3.8300E-07	1.6048E-08
Chisq	0.00451649	n/a
R^2	0.994773	n/a

Determination of order in [HBpin]

Figure S51. [*p*-MeOC$_6$H$_4$CH$_2$N(Bpin)$_2$] vs time; non-linear kinetics

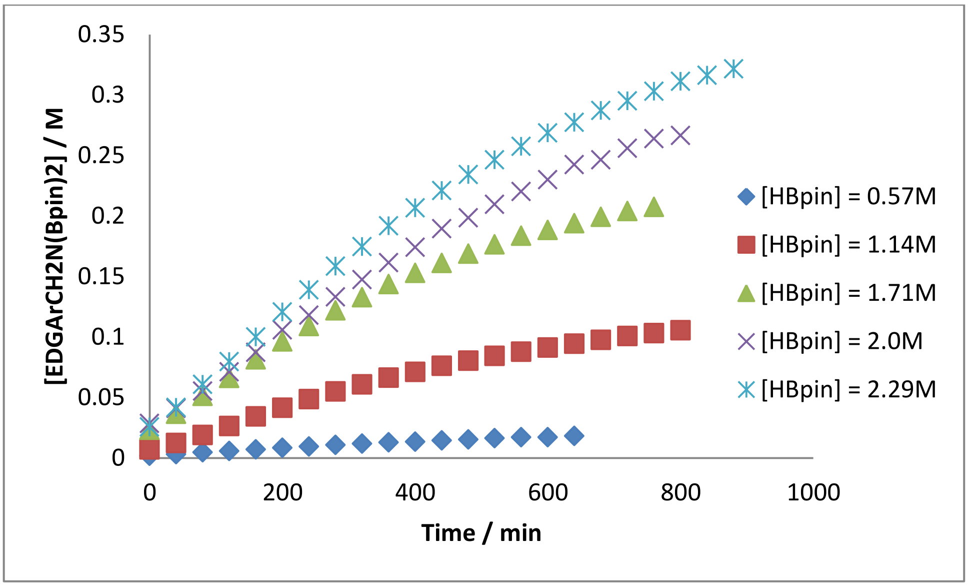

Figure S52. ln([p-MeOC$_6$H$_4$CH$_2$N(Bpin)$_2$]$_0$/[p-MeOC$_6$H$_4$CH$_2$N(Bpin)$_2$]$_t$) vs time; non-linear kinetics

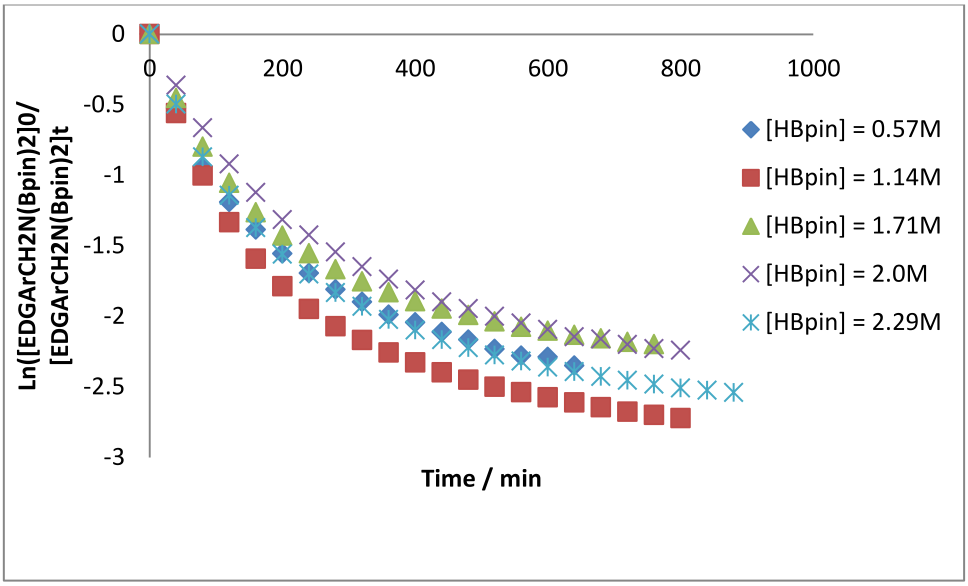

Figure S53. 1/[p-MeOC$_6$H$_4$CH$_2$N(Bpin)$_2$] vs time; non-linear kinetics

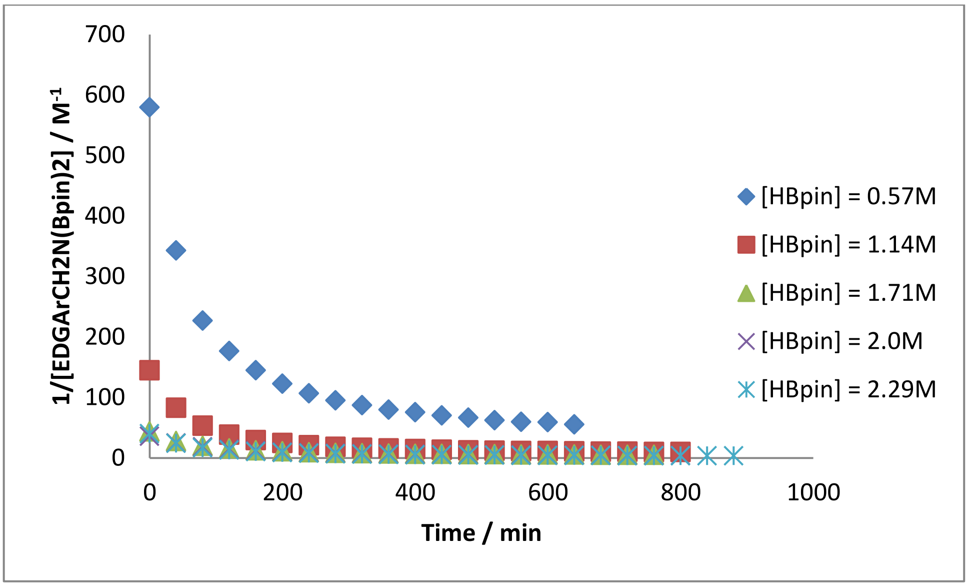

Figure S54. [*p*-$MeOC_6H_4CH_2N(Bpin)_2]^2$ vs time; induction period of 120 mins observed

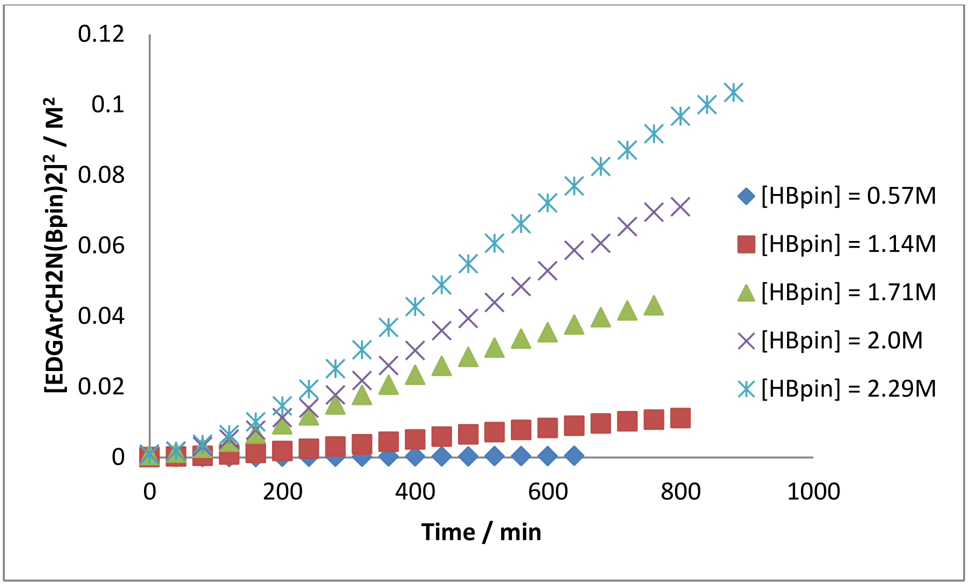

Figure S55. [*p*-$MeOC_6H_4CH_2N(Bpin)_2]^2$ vs time; induction period of 120 minutes removed

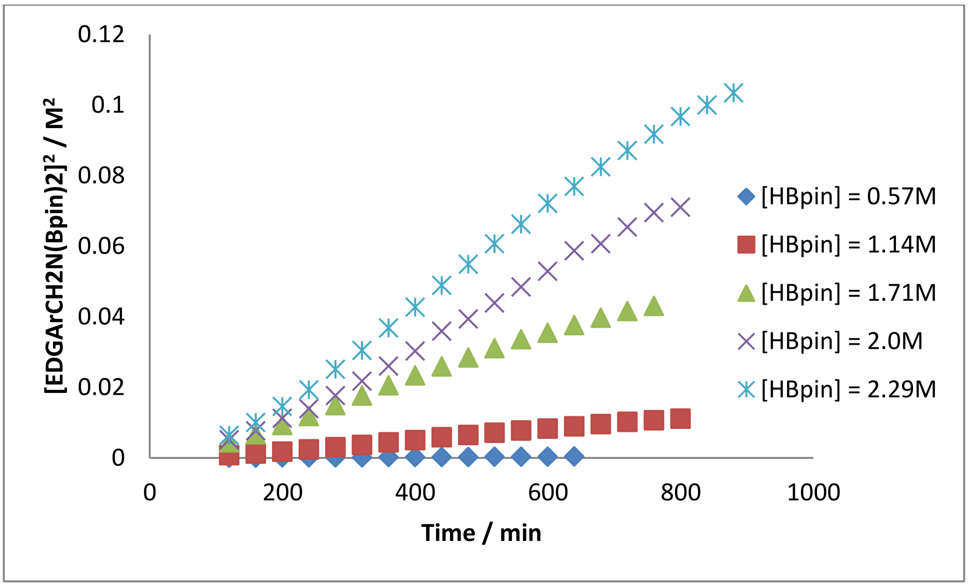

	[HBpin] = 0.57M	
	Value	Error
m_1	-0.000048	0.000004
m_2	0.000001	0.000000
Chisq	0.011388	n/a
R^2	0.9967516	n/a

	[HBpin] = 1.14M	
	Value	Error
m_1	-0.001327	0.000074
m_2	0.000016	0.000000
Chisq	0.001187	n/a
R^2	0.998651	n/a

	[HBpin] = 1.71M	
	Value	Error
m_1	-0.002571	0.000544
m_2	0.000063	0.000001
Chisq	0.002314	n/a
R^2	0.995136	n/a

	[HBpin] = 2.0M	
	Value	Error
m_1	-0.009919	0.000752
m_2	0.000104	0.000001
Chisq	0.014046	n/a
R^2	0.996698	n/a

	[HBpin] = 2.29M	
	Value	Error
m_1	-0.011227	0.000939
m_2	0.000135	0.000002
Chisq	0.001852	n/a
R^2	0.997134	n/a

Figure S56. [HBpin] vs k_{oba}; non-linear fit

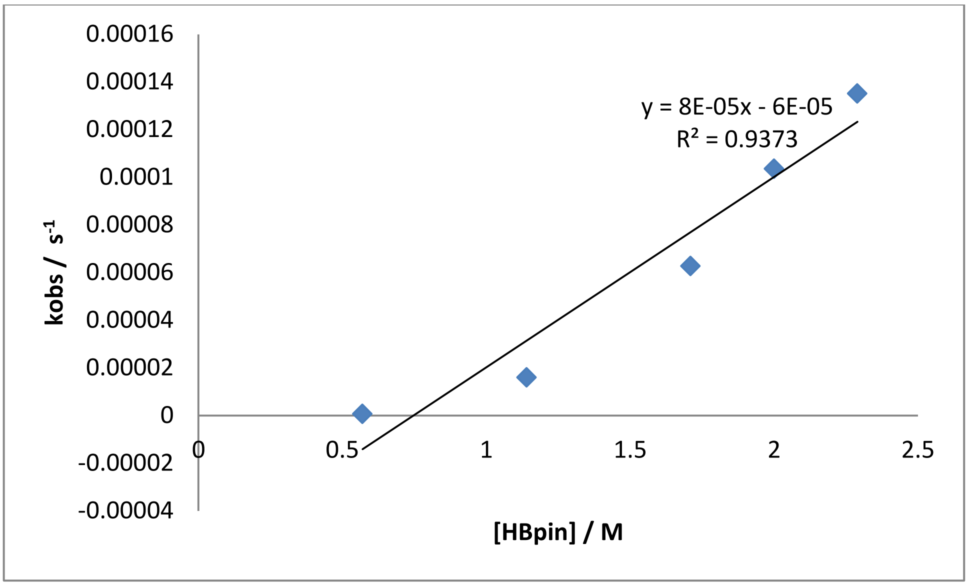

Figure S57. $[HBpin]^2$ vs k_{obs}; linear fit indicates a second order dependence on [HBpin]

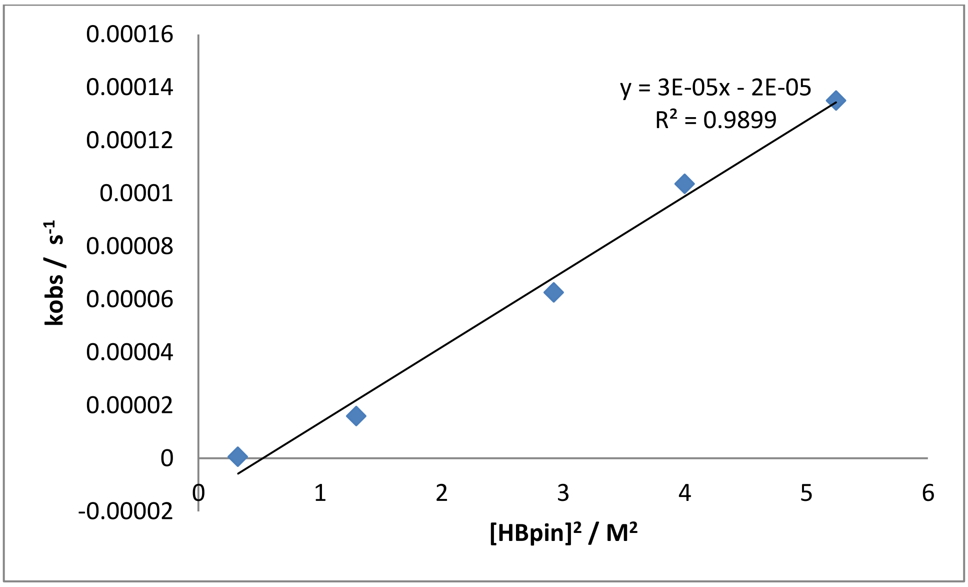

	Value	Error
m_1	-0.000015	0.000005
m_2	0.000028	0.000002
Chisq	0.015630413	n/a
R^2	0.98985985	n/a

Figure S58. $[HBpin]^3$ vs k_{obs}

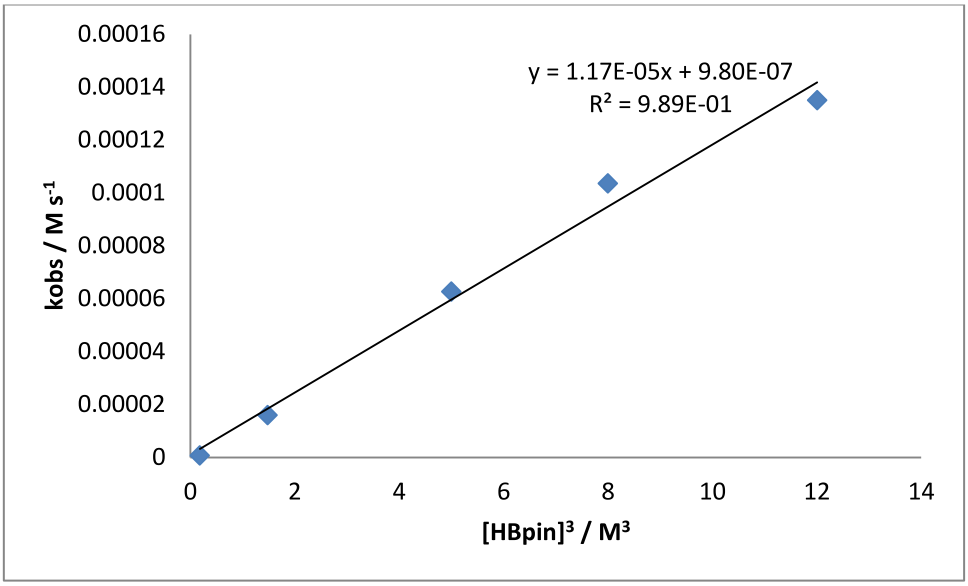

	Value	Error
m_1	9.80E-07	4.93E-06
m_2	1.17E-05	7.19E-07
Chisq	0.002553085	n/a
R^2	0.98884400	n/a

Variable [Mg] under *pseudo*-first order conditions in [HBpin]

Figure S59. [*p*-$MeOC_6H_4CN$] vs time; non-linear kinetics

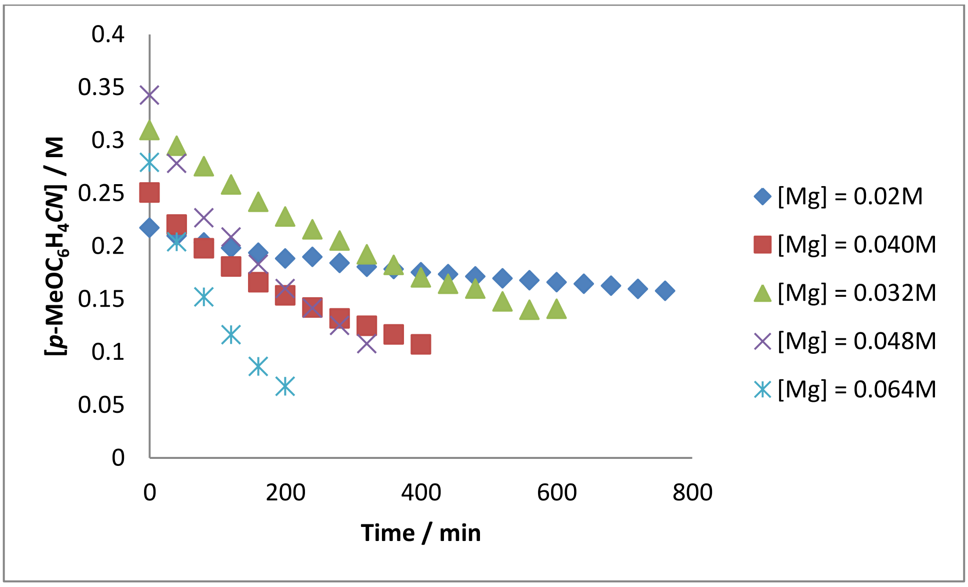

Figure S60. ln([p-MeOC$_6$H$_4$CN]$_0$/[p-MeOC$_6$H$_4$CN]$_t$) vs time; variable [Mg] under *pseudo*-first order conditions in [HBpin] (8.0 M) whilst keeping [p-MeOC$_6$H$_4$CN] (0.4 M) invariant

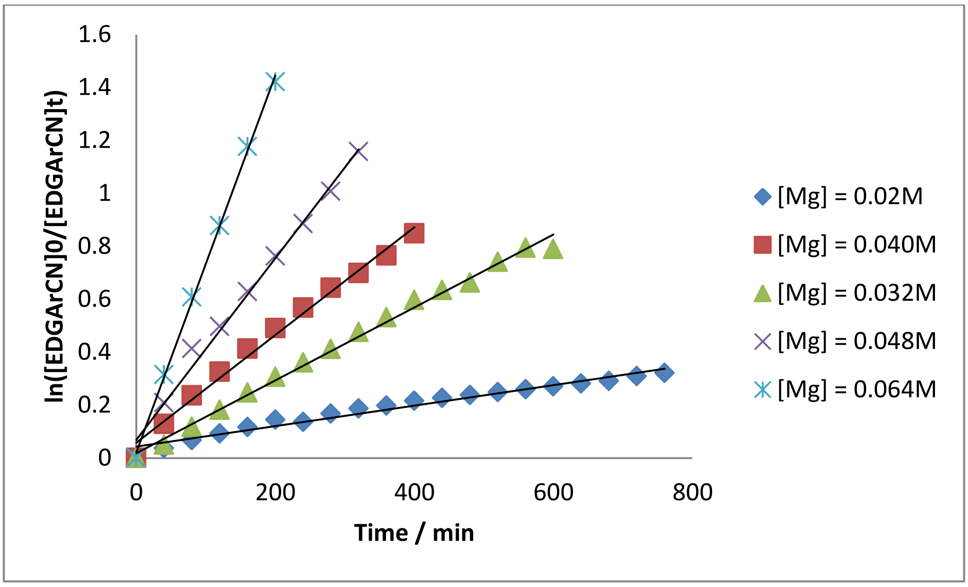

	[Mg] = 0.020M	
	Value	Error
m_1	0.043055	0.007416
m_2	0.000386	0.000017
Chisq	0.225892	n/a
R^2	0.967518	n/a

	[Mg] = 0.040M	
	Value	Error
m_1	0.057696	0.016640
m_2	0.002037	0.000070
Chisq	0.049623	n/a
R^2	0.989386	n/a

	[Mg] = 0.032M	
	Value	Error
m_1	0.018183	0.016640
m_2	0.001376	0.000070
Chisq	0.004929	n/a
R^2	0.993879	n/a

	[Mg] = 0.048M	
	Value	Error
m_1	0.068100	0.023870
m_2	0.003433	0.000125
Chisq	0.036552	n/a
R^2	0.990754	n/a

	[Mg] = 0.064M	
	Value	Error
m_1	0.021445	0.014694
m_2	0.007113	0.000121
Chisq	0.001945	n/a
R^2	0.998838	n/a

Figure S61. 1/[*p*-$MeOC_6H_4CN$] vs time; non-linear kinetics

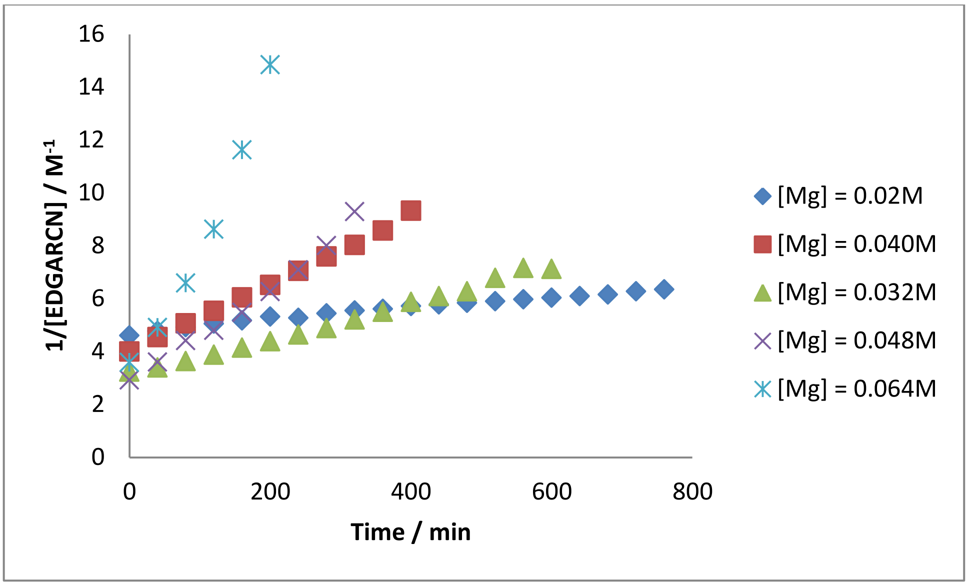

Figure S62. [Mg] vs k_{obs}; non-linear fit. k_{obs} values taken from 1st order plot

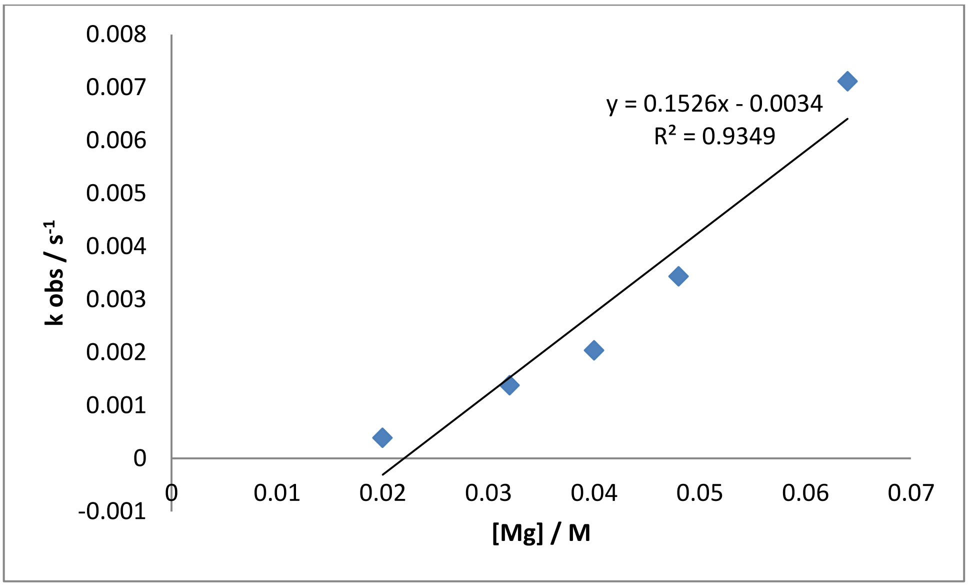

Figure S63. $[Mg]^2$ vs k_{obs}

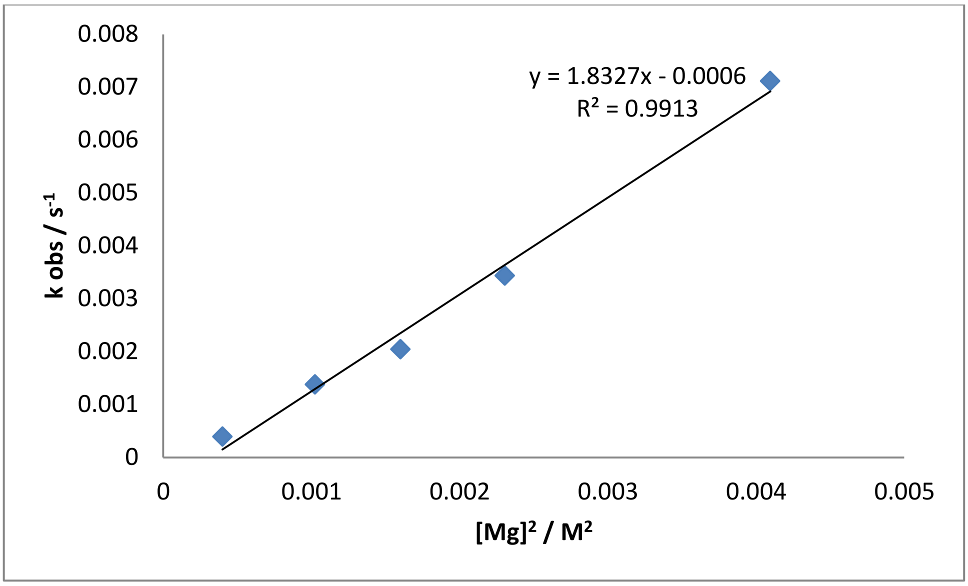

	Value	Error
m_1	-0.000585	0.000225
m_2	1.832664	0.099105
Chisq	0.010325	n/a
R^2	0.991303	n/a

Figure S64. $[Mg]^3$ vs k_{obs}

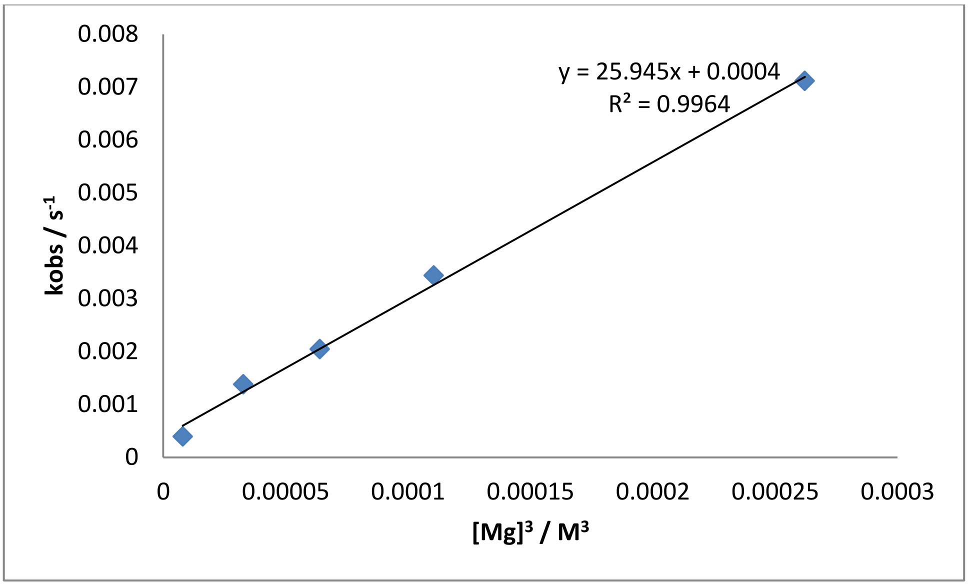

	Value	Error
m_1	0.000391	0.000119
m_2	25.944599	0.904880
Chisq	0.008239727	n/a
R^2	0.996364	n/a

Variable [HBpin] under near stoichiometric reaction conditions

Figure S65. [*p*-MeOC_6H_4CN] vs time; non-linear kinetics

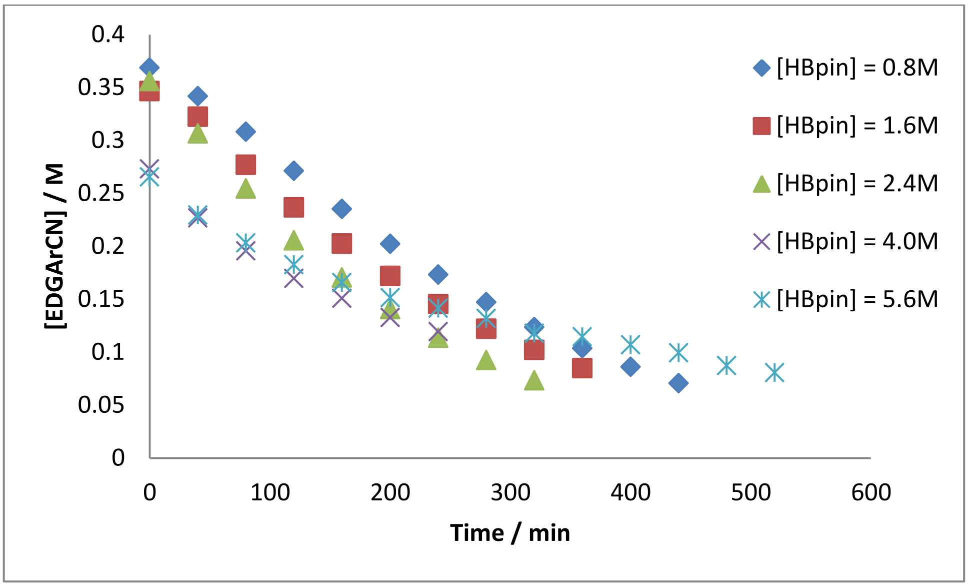

Figure S66. ln([*p*-MeOC_6H_4CN]$_0$/[*p*-MeOC_6H_4CN]$_t$) vs time

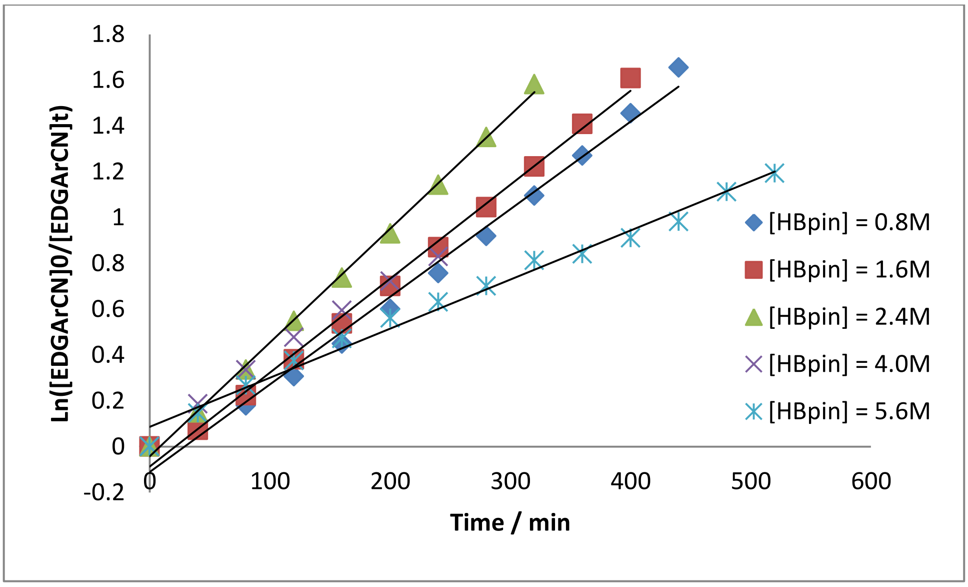

	[HBpin] = 0.8M	
	Value	Error
m_1	-0.095968	0.028964
m_2	0.003711	0.000122
Chisq	0.041384	n/a
R^2	0.990307	n/a

	[HBpin] = 1.6M	
	Value	Error
m_1	-0.075344	0.021288
m_2	0.004009	0.000100
Chisq	0.026625	n/a
R^2	0.995078	n/a

	[HBpin] = 2.4M	
	Value	Error
m_1	-0.042223	0.015034
m_2	0.004969	0.000079
Chisq	0.006756	n/a
R^2	0.998236	n/a

	[HBpin] = 4.0M	
	Value	Error
m_1	0.039869	0.019001
m_2	0.003409	0.000132
Chisq	0.021209	n/a
R^2	0.992589	n/a

	[HBpin] = 5.6M	
	Value	Error
m_1	0.086297	0.020508
m_2	0.002142	0.000067
Chisq	0.061684	n/a
R^2	0.988388	n/a

Figure S67. 1/[*p*-$MeOC_6H_4CN$] vs time; non-linear kinetics

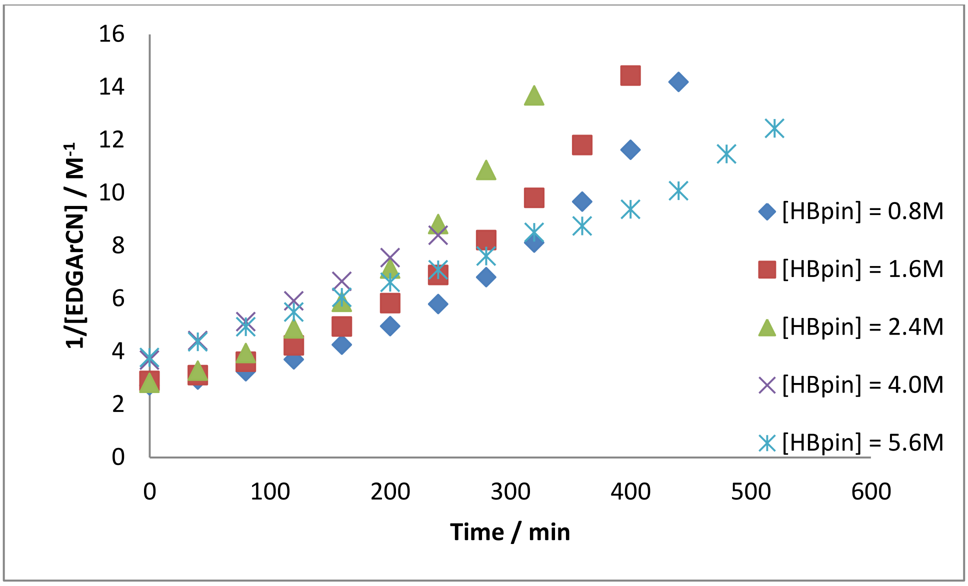

Figure S68. [HBpin] vs k_{obs}; indicates a variable dependence upon [HBpin] with respect to rate of reaction

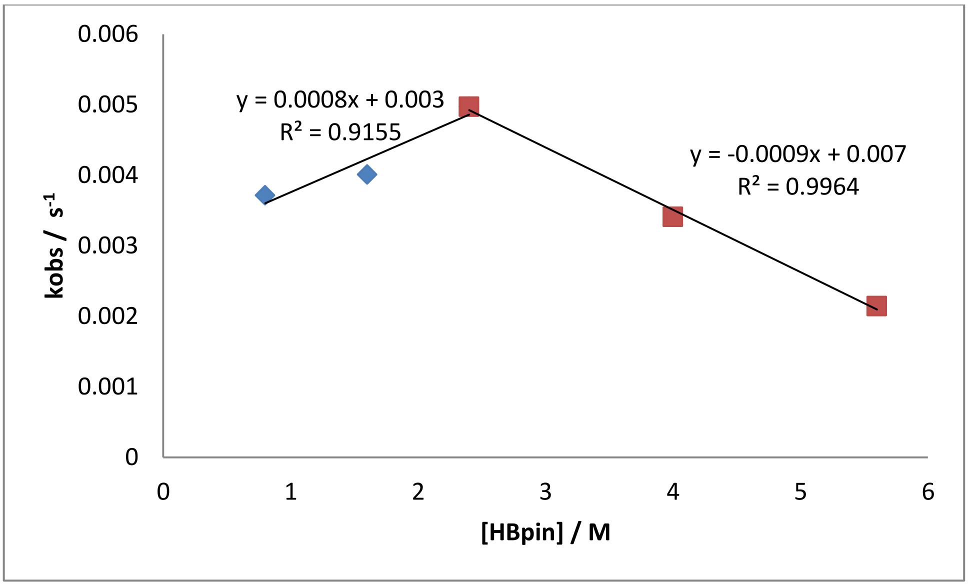

	[HBpin] 0.8 – 2.4M	
	Value	Error
m_1	0.002957	0.000413
m_2	0.000794	0.000239
Chisq	0.048987	n/a
R^2	0.919695	n/a

	[HBpin] = 2.4 – 5.6M	
	Value	Error
m_1	0.007081	0.000222
m_2	-0.000897	0.000053
Chisq	0.001245	n/a
R^2	0.997477	n/a

Figure S69. [HBpin]^2 vs k_{obs}; increased linearity for 0.8-2.4M

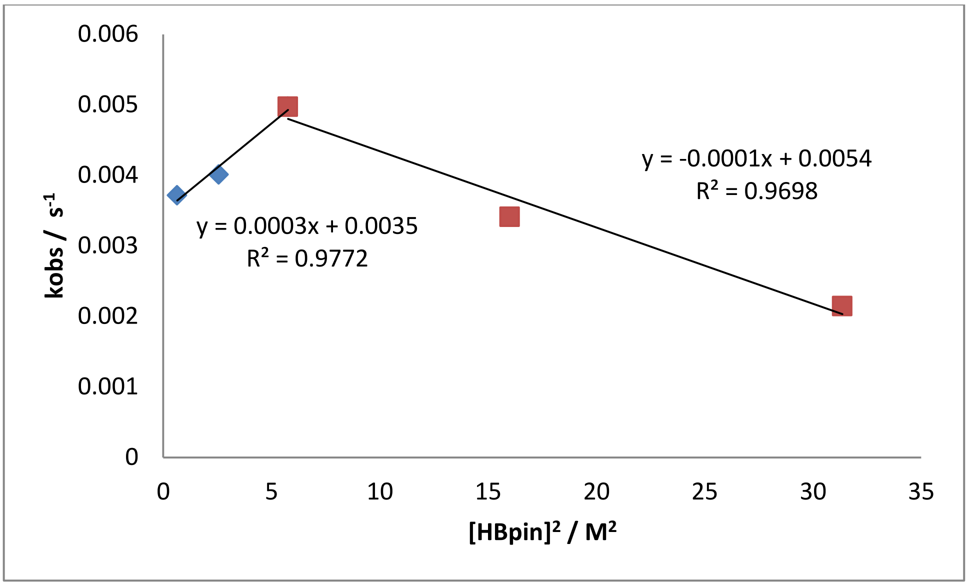

	[HBpin] 0.8 – 2.4M	
	Value	Error
m_1	0.003479	0.000140
m_2	0.000251	0.000038
Chisq	0.017675	n/a
R^2	0.977190	n/a

	[HBpin] = 2.4 – 5.6M	
	Value	Error
m_1	0.005423	0.000393
m_2	-0.000108	0.000019
Chisq	0.021134	n/a
R^2	0.969779	n/a

Figure S70. [HBpin]^3 vs k_{obs}; increased linearity for 0.8 - 2.4M

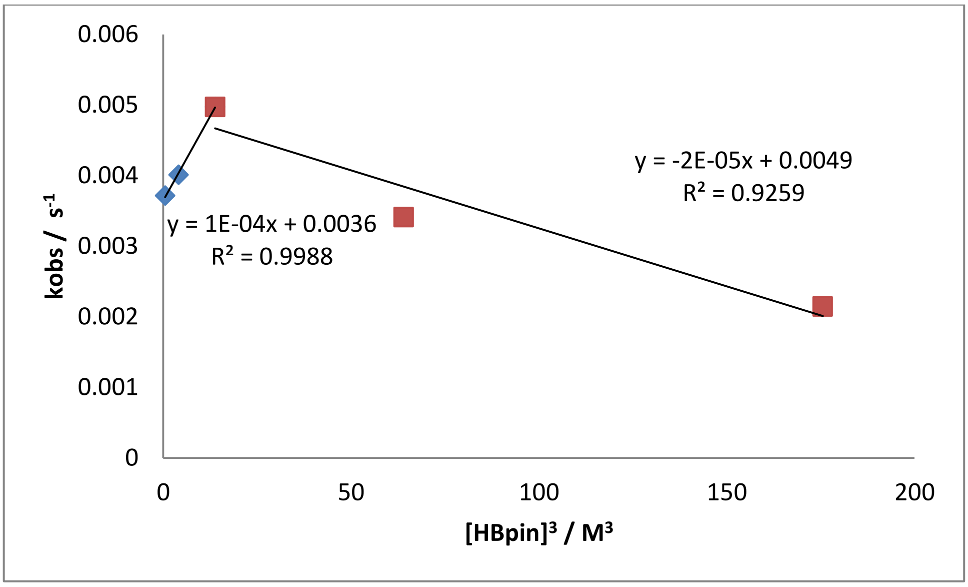

	[HBpin] 0.8 – 2.4M	
	Value	Error
m_1	0.003644	0.000027
m_2	0.000095	0.000003
Chisq	0.00117	n/a
R^2	0.998807	n/a

	[HBpin] = 2.4 – 5.6M	
	Value	Error
m_1	0.004897	0.000503
m_2	-0.000016	0.000005
Chisq	0.064303	n/a
R^2	0.925949	n/a

Variable Temperature Studies

Figure S71. [*p*-$MeOC_6H_4CN$] vs time; non-linear kinetics

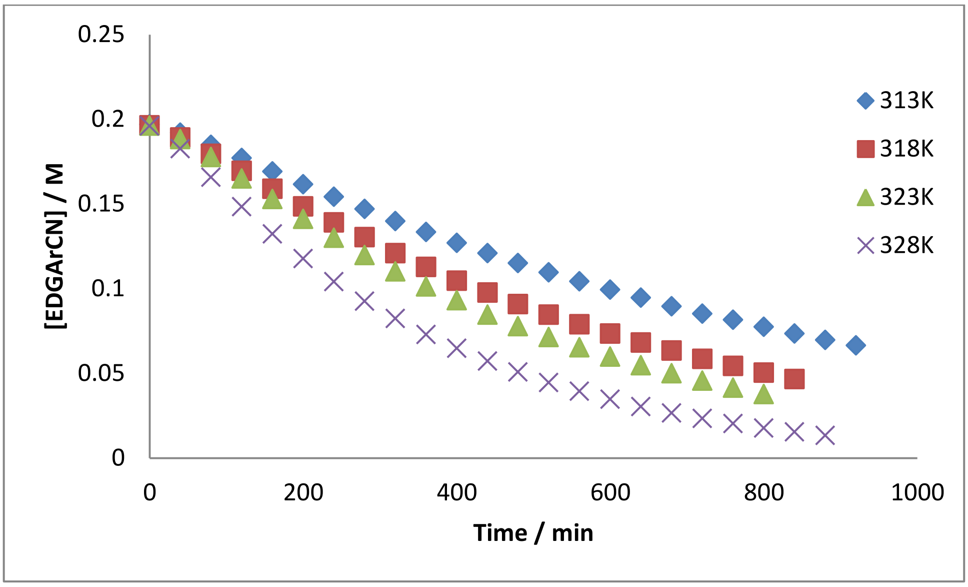

Figure S72. ln([*p*-$MeOC_6H_4CN]_0$/[*p*-$MeOC_6H_4CN]_t$) vs time

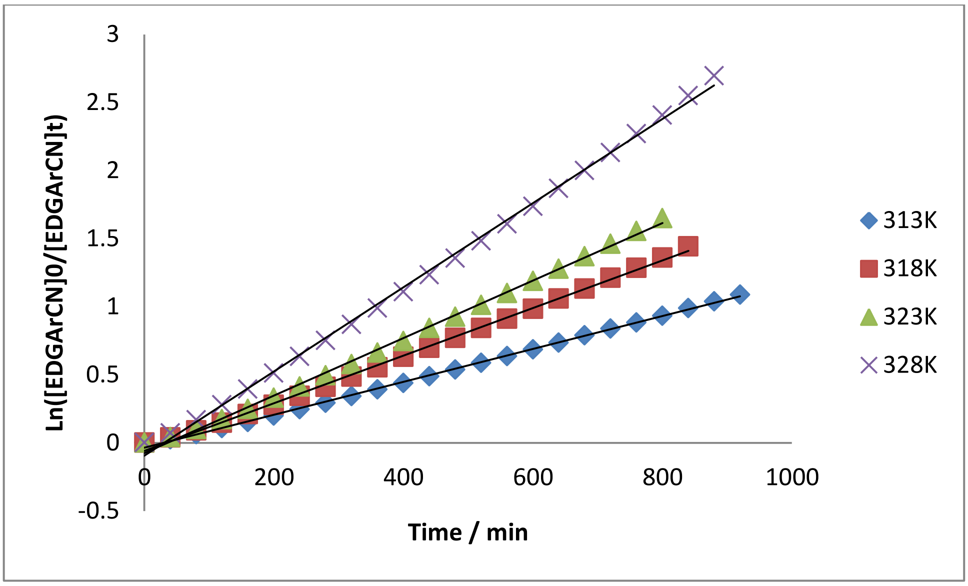

	313 K	
	Value	Error
m_1	-0.036570	0.004240
m_2	0.001210	0.000008
Chisq	0.011917	n/a
R^2	0.99906	n/a

	318 K	
	Value	Error
m_1	-0.058620	0.008199
m_2	0.001750	0.000017
Chisq	0.017399	n/a
R^2	0.99818	n/a

	323 K	
	Value	Error
m_1	-0.074960	0.010727
m_2	0.002110	0.000023
Chisq	0.021446	n/a
R^2	0.99776	n/a

	328 K	
	Value	Error
m_1	-0.056910	0.010357
m_2	0.002930	0.000031
Chisq	0.012625	n/a
R^2	0.99850	n/a

Figure S73. 1/[*p*-$MeOC_6H_4CN$] vs time; non-linear kinetics

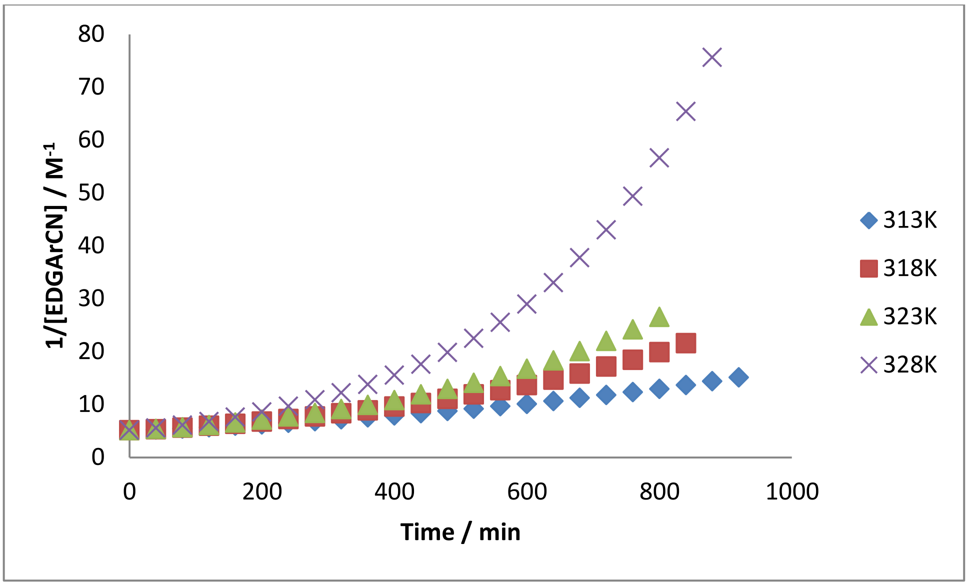

Figure S74. Eyring Plot; 1/T vs Ln(k/T)

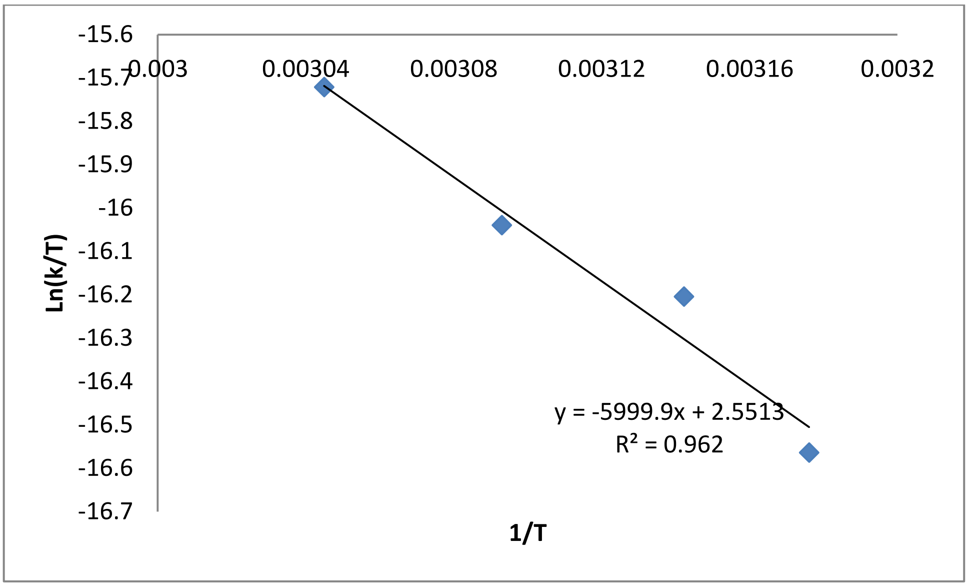

	Value	Error
m_1	2.551282	2.627555
m_2	-5999.875772	843.654844
Chisq	0.038217588	n/a
R^2	0.961961	n/a

The graph shown as Fig. S74 was used to calculate the following Activation Energy Parameters, least square error analysis was also carried to provide accurate error information.

	Value	Error
ΔH	49.88 kJ mol^{-1}	± 7.01
ΔS	-176.33 J k^{-1} mol^{-1}	± 21.85

Figure S75. Arrhenius Plot; 1/T vs Ln(k)

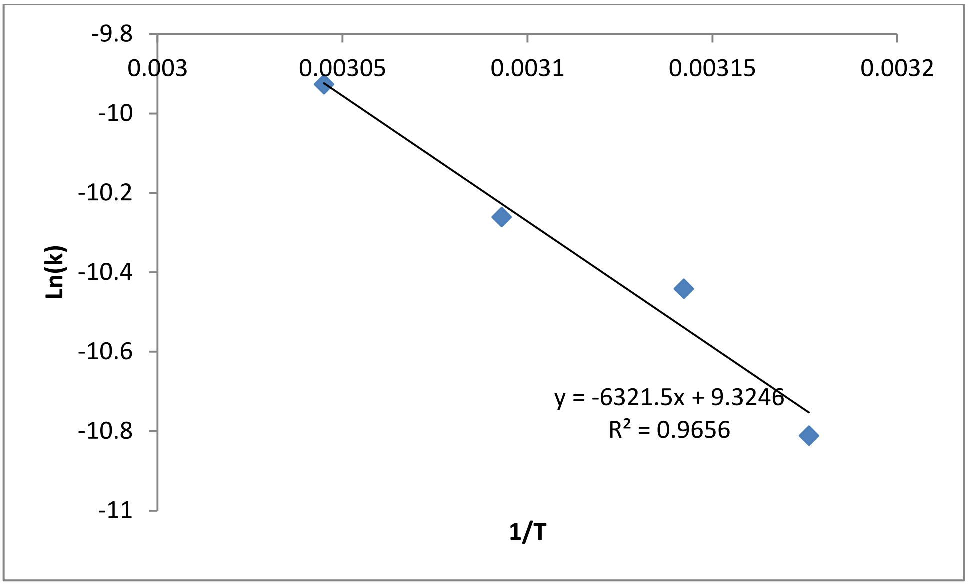

	Value	Error
m_1	9.324641	2.625809
m_2	-6321.450699	843.094344
Chisq	0.034447917	n/a
R^2	0.965647	n/a

This graph was used to calculate the following Activation Energy Parameter; least square error analysis was also carried to provide accurate error information.

	Value	Error
Ea	52.56 kJ mol^{-1}	± 7.01

Electron Withdrawing aryl nitrile – *m*-$MeOC_6H_4CN$

Determination of order in [Mg]

Figure S76. [*m*-$MeOC_6H_4CN$] vs time; non-linear kinetics

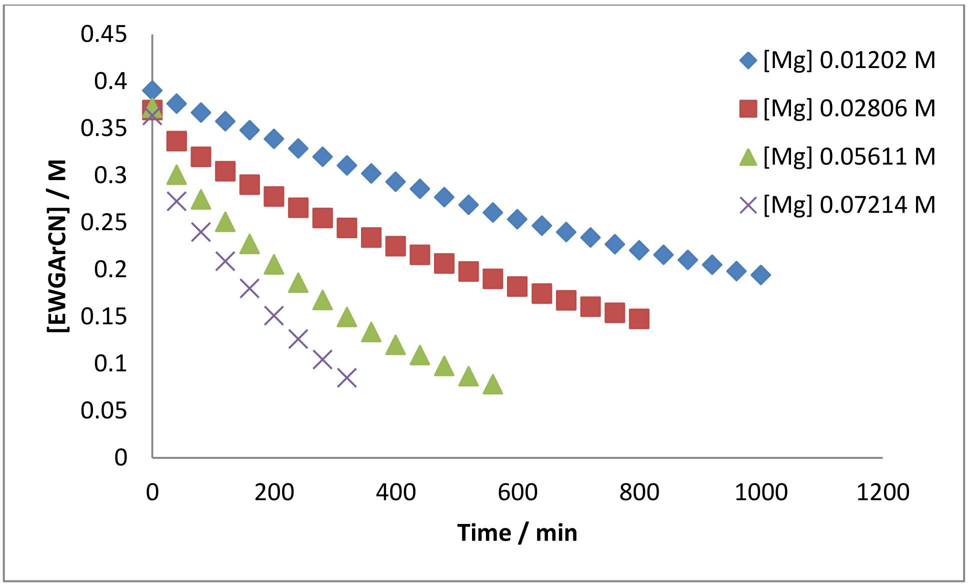

Figure S77. ln([*m*-$MeOC_6H_4CN]_0$/[*m*-$MeOC_6H_4CN]_t$) vs time

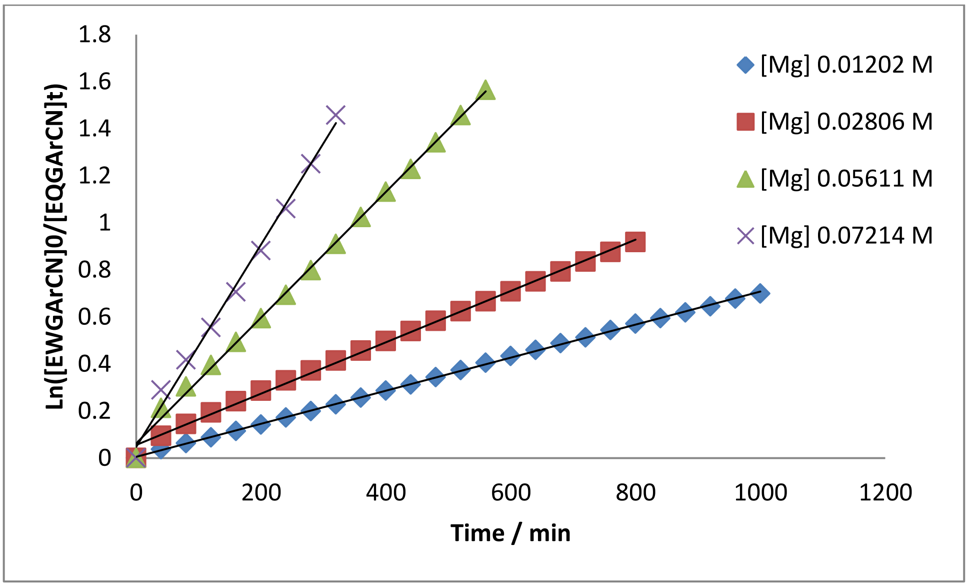

	[Mg] 0.01202 M	
	Value	Error
m_1	0.004713	0.001563
m_2	0.000702	0.000003
Chisq	0.0005	n/a
R^2	0.999650	n/a

	[Mg] 0.02806 M	
	Value	Error
m_1	0.054630	0.006236
m_2	0.001090	0.000013
Chisq	0.042573	n/a
R^2	0.99717	n/a

	[Mg] 0.05611 M	
	Value	Error
m_1	0.061530	0.011542
m_2	0.002670	0.000035
Chisq	0.017716	n/a
R^2	0.99776	n/a

	[Mg] 0.0.07214 M	
	Value	Error
m_1	0.046350	0.024226
m_2	0.004300	0.000127
Chisq	0.010818	n/a
R^2	0.99392	n/a

Figure S78. 1/[*m*-$MeOC_6H_4CN$] vs time; non-linear kinetics

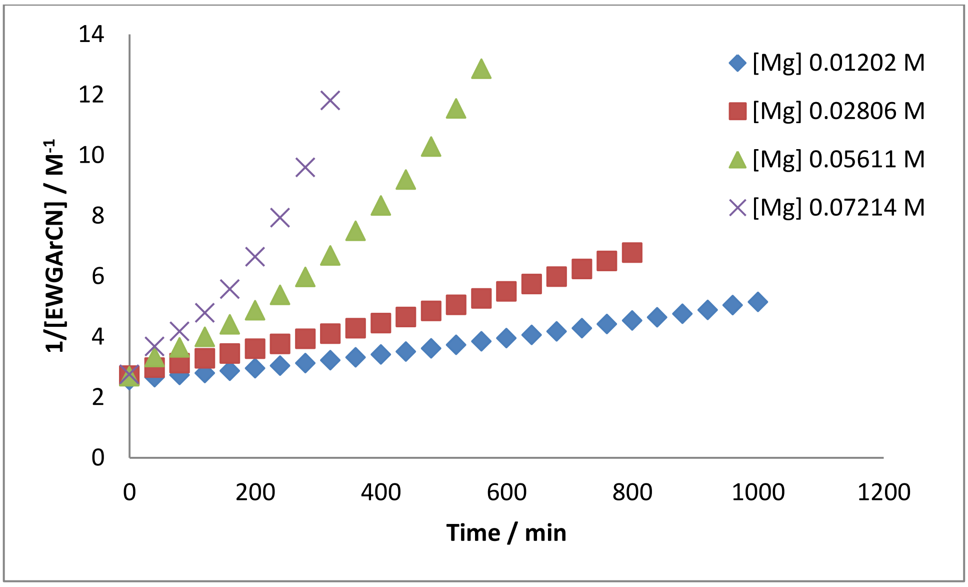

Figure S79. [Mg] vs k_{obs}; non-linear fit

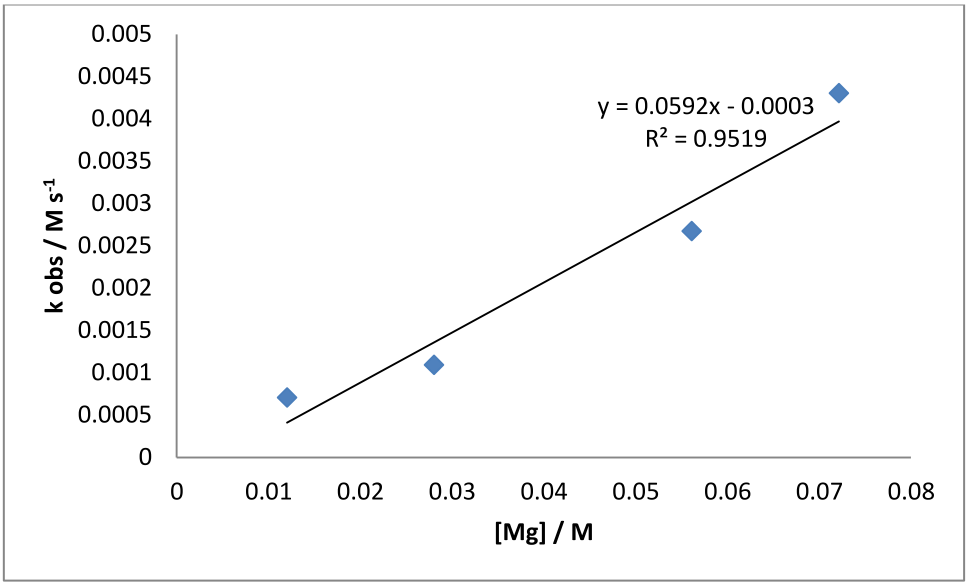

Figure S80. $[Mg]^2$ vs k_{obs}; indicating a second order dependence upon [Mg]

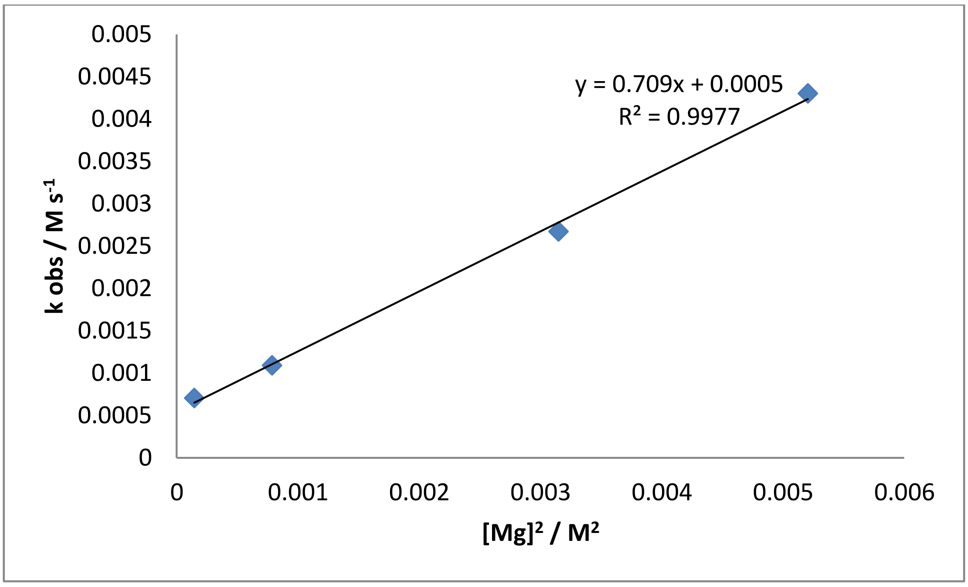

	Value	Error
m_1	0.000545	0.000074
m_2	0.708969	0.024210
Chisq	0.001466	n/a
R^2	0.997673	n/a

Figure S81. $[Mg]^{1/2}$ vs k_{obs}; non-linear fit

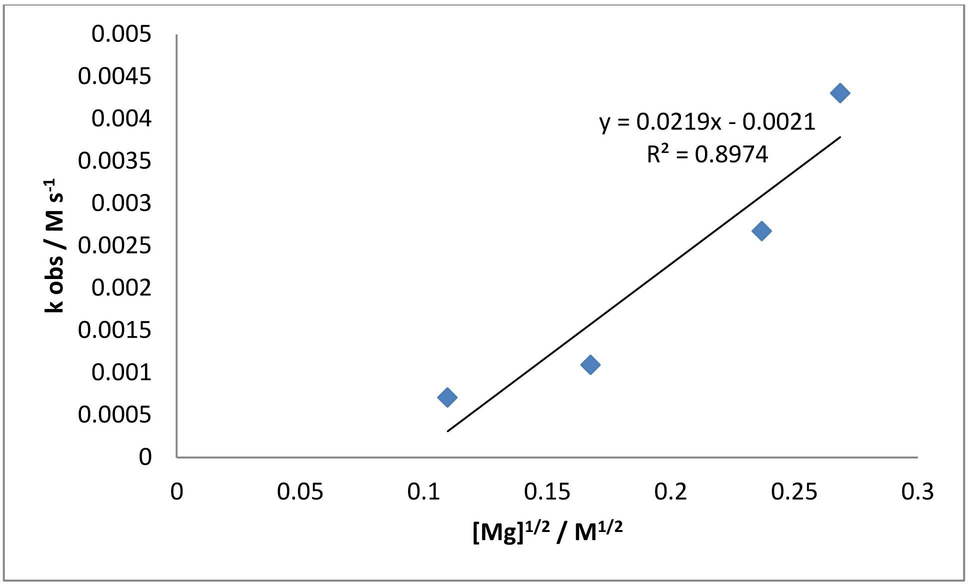

Determination of order in [Ar(EWG)CN]

Figure S82. [*m*-$MeOC_6H_4CN$] vs time; non-linear fit

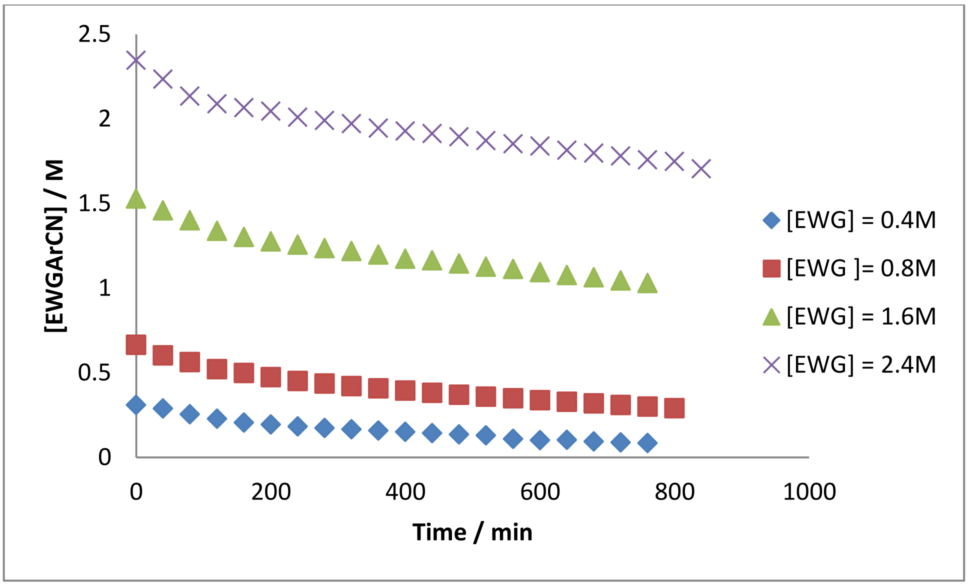

Figure S83. ln([*m*-$MeOC_6H_4CN$]$_0$/[*m*-$MeOC_6H_4CN$]$_t$) vs time; linear fit with induction period 0-160 min

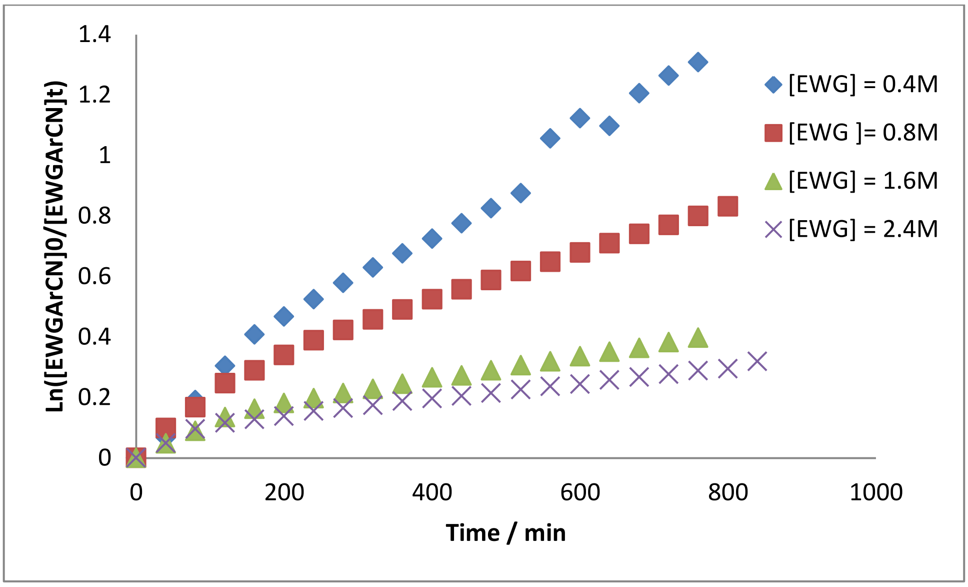

Figure S84. ln([*m*-$MeOC_6H_4CN$]$_0$/[*m*-$MeOC_6H_4CN$]$_t$) vs time; induction period omitted for k_{obs} values

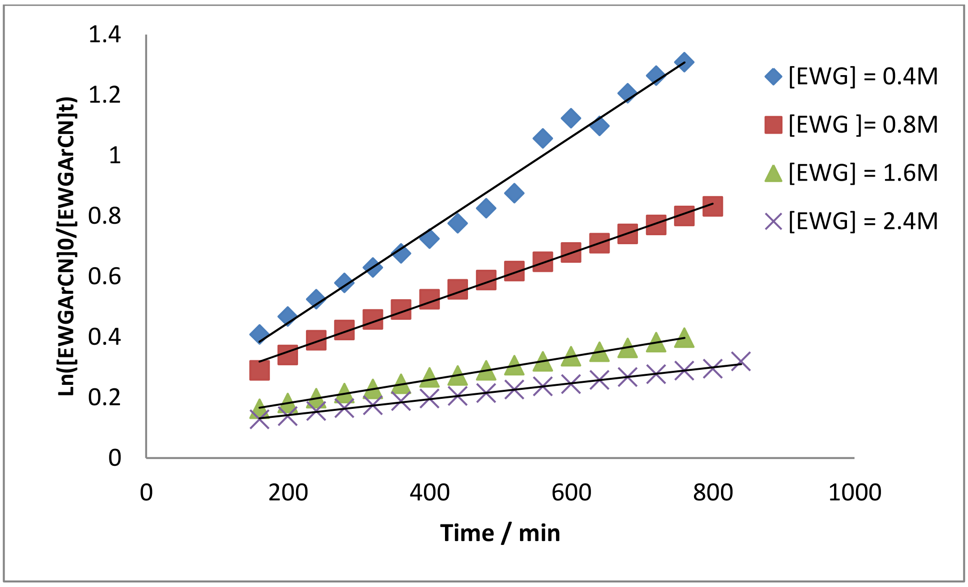

	[EWG] 0.4M	
	Value	Error
m_1	0.137358	0.024792
m_2	0.001540	0.000050
Chisq	0.006875	n/a
R^2	0.985440	n/a

	[EWG] 0.8M	
	Value	Error
m_1	0.188078	0.006876
m_2	0.000816	0.000013
Chisq	0.03394	n/a
R^2	0.996050	n/a

	[EWG] 1.6M	
	Value	Error
m_1	0.104483	0.001651
m_2	0.000385	0.000003
Chisq	0.003903	n/a
R^2	0.998953	n/a

	[EWG] 2.4M	
	Value	Error
m_1	0.088384	0.002056
m_2	0.000264	0.000004
Chisq	0.003444	n/a
R^2	0.996701	n/a

Figure S85. 1/[*m*-$MeOC_6H_4CN$] vs time; non-linear fit

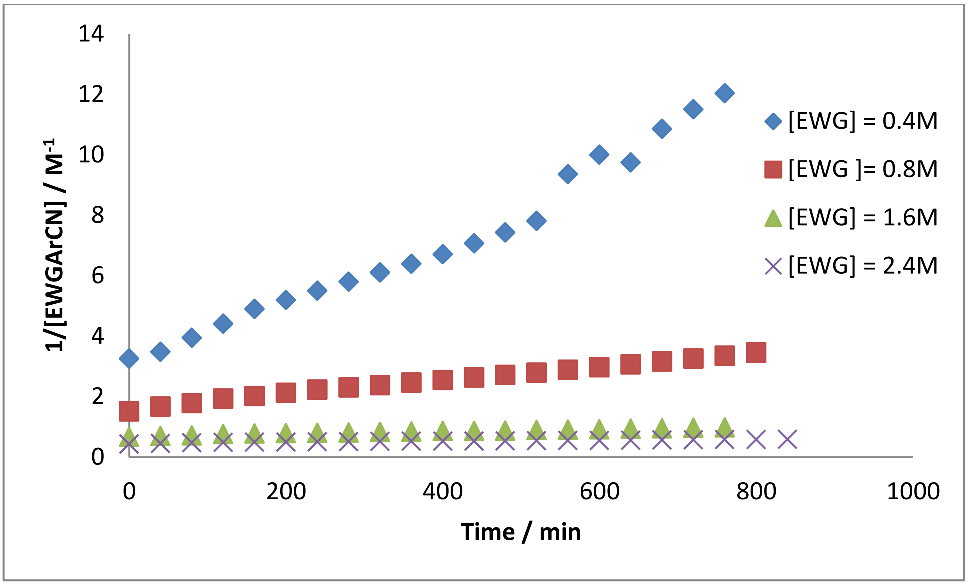

Figure S86. [*m*-$MeOC_6H_4CN$] vs k_{obs}; non-linear kinetics

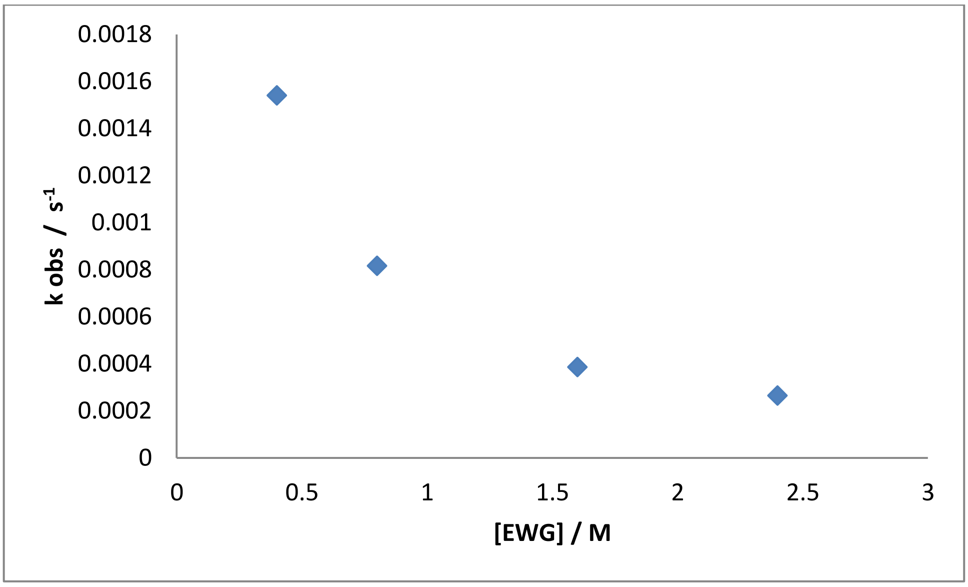

Figure S87. [*m*-$MeOC_6H_4CN$]$^{-1}$ vs k_{obs}; indicating a -1 order dependence upon [Ar(EWG)CN]

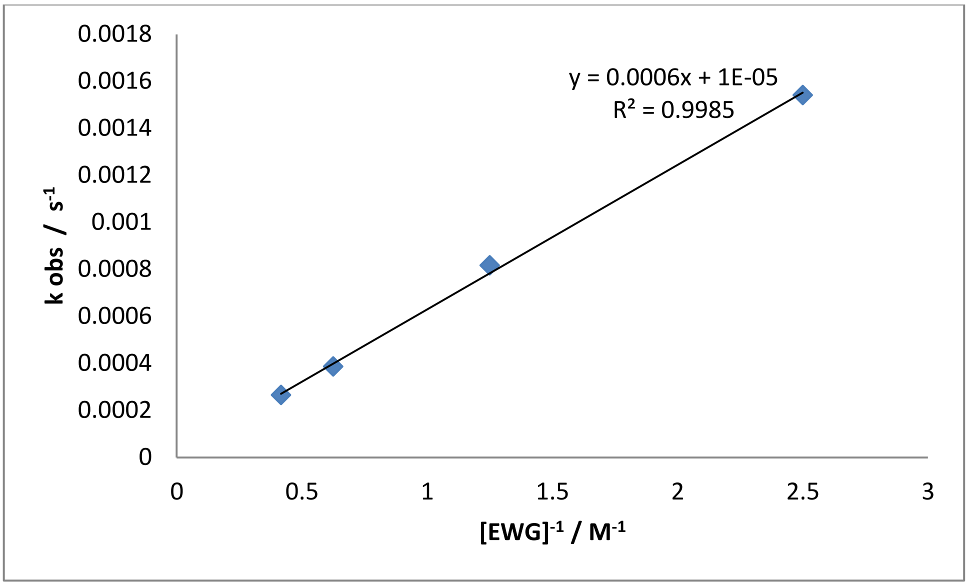

	Value	Error
m_1	0.000015	0.000024
m_2	0.000615	0.000017
Chisq	0.000626	n/a
R^2	0.998542	n/a

Variable [Mg] under *pseudo*-first order in [HBpin]

Figure S88. [*m*-MeOC_6H_4CN] vs time; non-linear fit

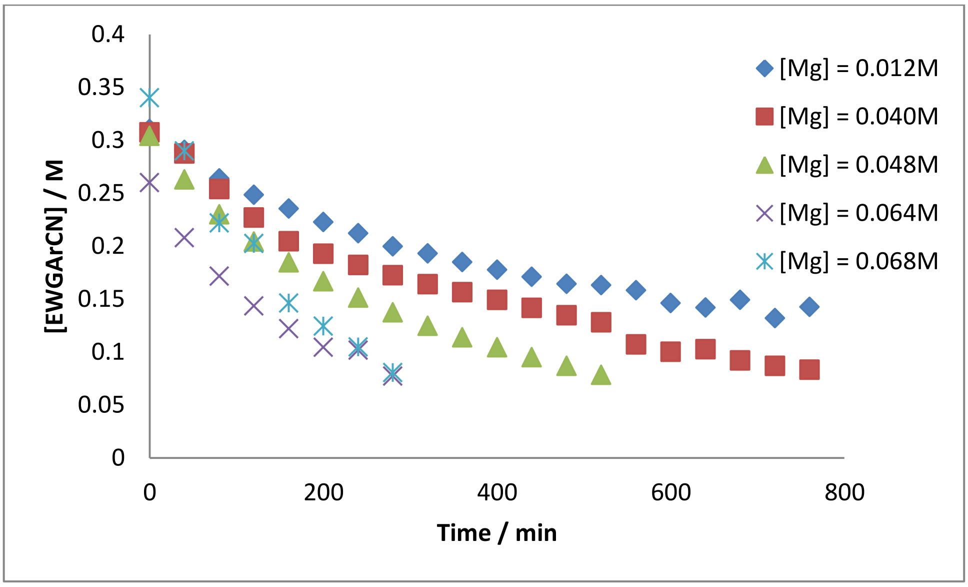

Figure S891. ln([*m*-MeOC_6H_4CN]$_0$/[*m*-MeOC_6H_4CN]$_t$) vs time; induction period observed at lower [Mg]

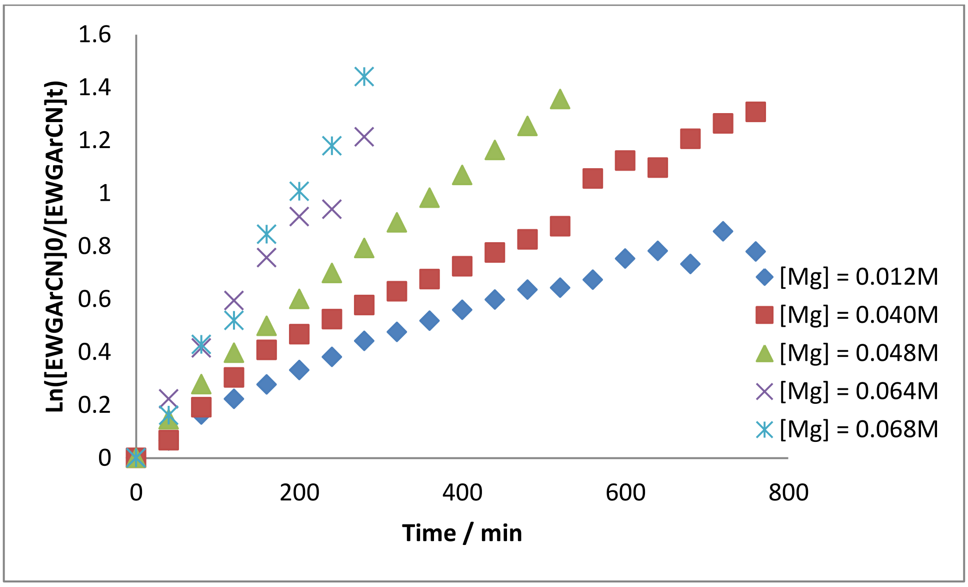

Figure S90. ln([*m*-$MeOC_6H_4CN$]$_0$/[*m*-$MeOC_6H_4CN$]$_t$) vs time; induction period of 120 min removed for 2 lowest [Mg]

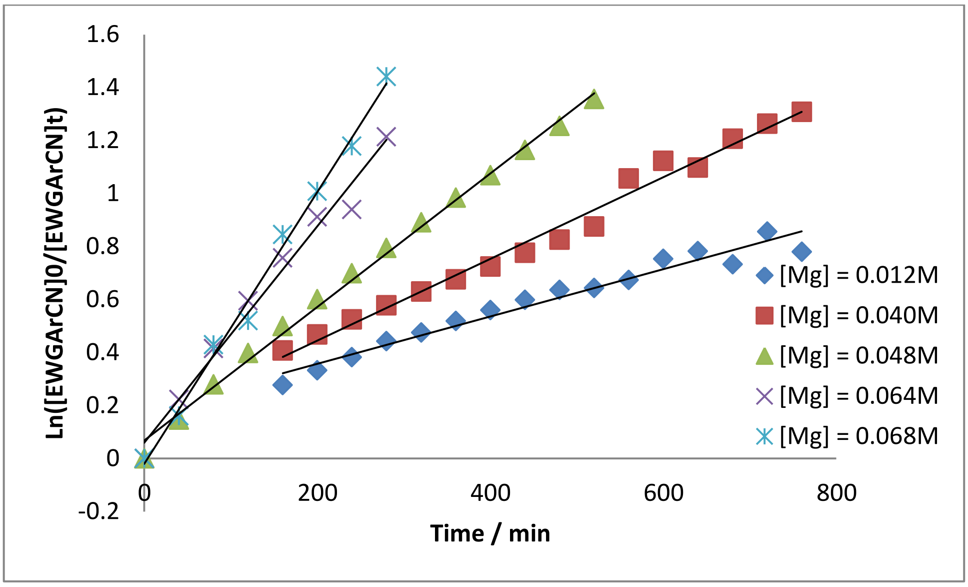

	[Mg] 0.012M	
	Value	Error
m_1	0.179299	0.024145
m_2	0.000892	0.000049
Chisq	0.072735	n/a
R^2	0.959934	n/a

	[Mg] 0.040M	
	Value	Error
m_1	0.137358	0.024792
m_2	0.001540	0.000050
Chisq	0.006875	n/a
R^2	0.985440	n/a

	[Mg] 0.048M	
	Value	Error
m_1	0.068124	0.014779
m_2	0.002520	0.000048
Chisq	0.027985	n/a
R^2	0.995610	n/a

	[Mg] 0.064M	
	Value	Error
m_1	0.059523	0.036864
m_2	0.004084	0.000220
Chisq	0.024854	n/a
R^2	0.982841	n/a

	[Mg] 0.068M	
	Value	Error
m_1	-0.020818	0.028766
m_2	0.005130	0.000172
Chisq	0.001947	n/a
R^2	0.993308	n/a

Figure S91. 1/[m-$MeOC_6H_4CN$] vs time; non-linear kinetics

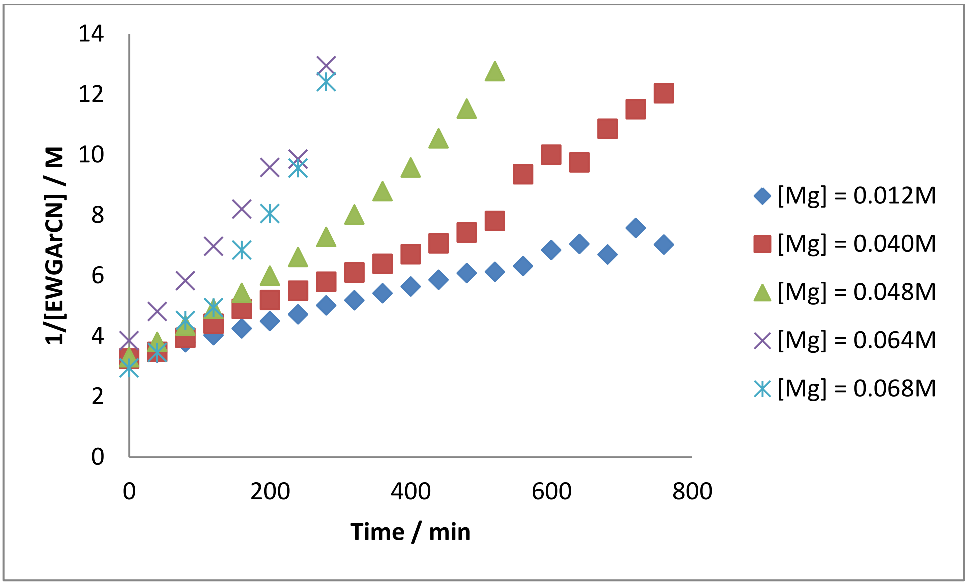

Figure S92. [Mg] vs k_{obs}; non-linear fit

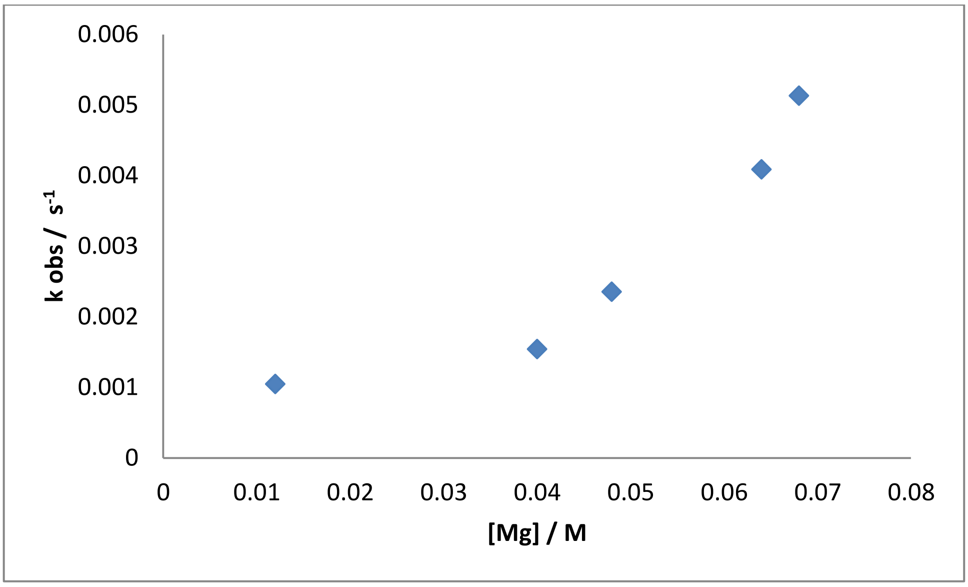

Figure S93. $[Mg]^2$ vs k_{obs}; non-linear fit

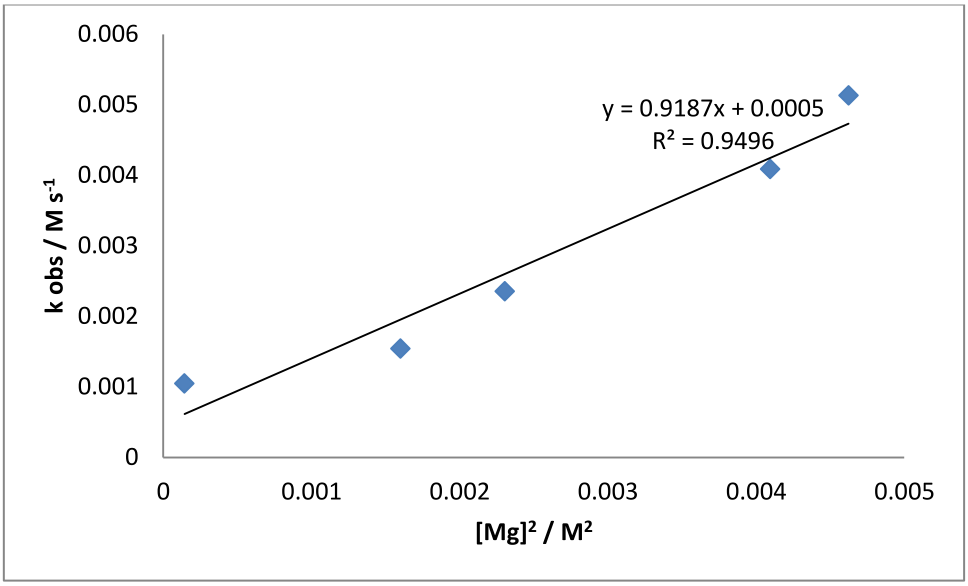

Figure S94. $[Mg]^3$ vs k_{obs}; indicated 3rd order dependence upon [Mg] under *pseudo*-first order conditions in [HBpin]

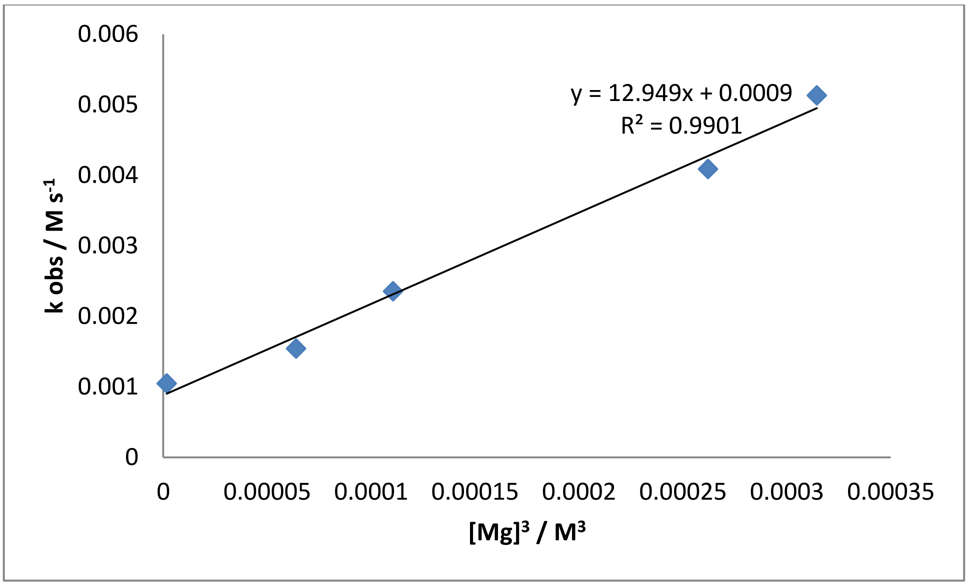

	Value	Error
m_1	0.000879	0.000143
m_2	12.949454	0.746753
Chisq	0.008512641	n/a
R^2	0.990122	n/a

Variable [Mg] under *pseudo*-first order in [*m*-MeOC_6H_4CN]

Figure S95. [*m*-MeO$C_6H_4CH_2N(Bpin)_2$] vs time; non-linear kinetics

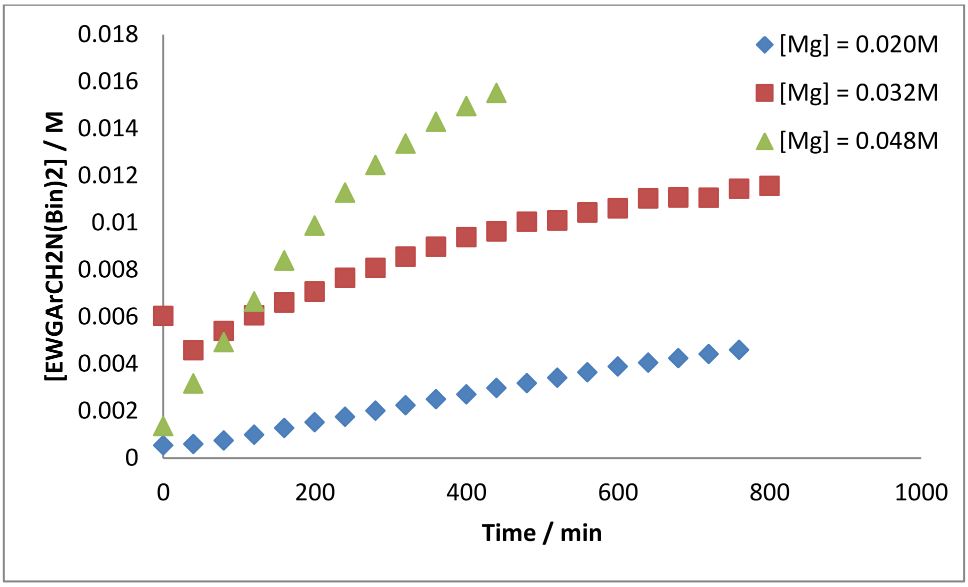

Figure S96. ln([*m*-MeO$C_6H_4CH_2N(Bpin)_2]_0$/[*m*-MeO$C_6H_4CH_2N(Bpin)_2]_t$) vs time; non-linear kinetics

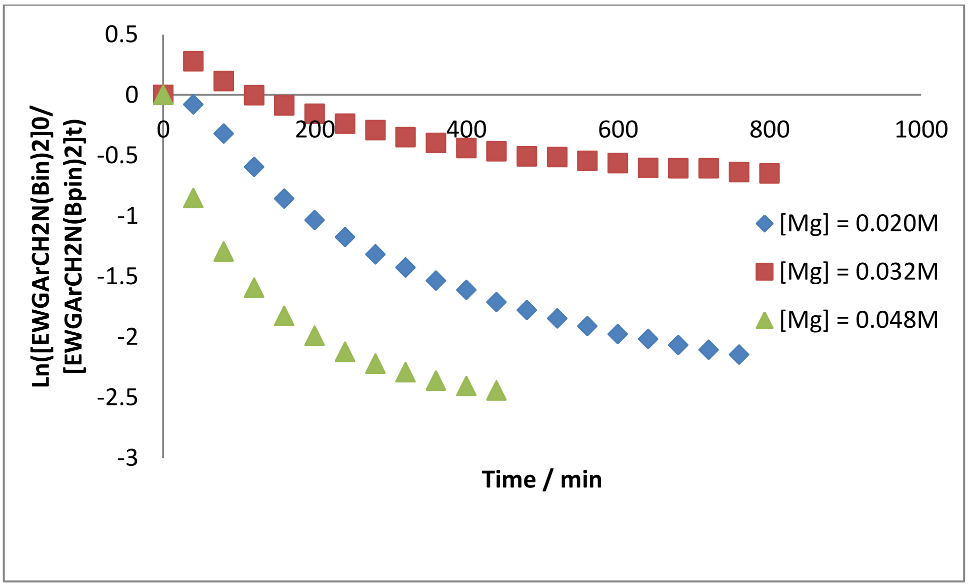

Figure S97. 1/[m-MeOC$_6$H$_4$CH$_2$N(Bpin)$_2$] vs time; non-linear kinetics

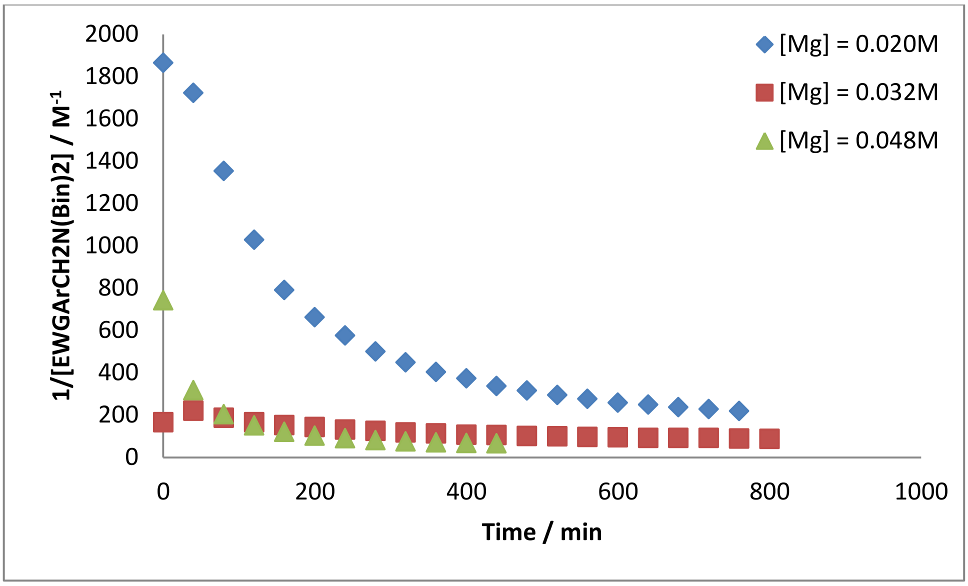

Figure S98. [m-MeOC$_6$H$_4$CH$_2$N(Bpin)$_2$]^2 vs time; variable [Mg] under *pseudo*-first order conditions in [m-MeOC$_6$H$_4$CN] (4.0M) whilst keeping [HBpin] (0.8M) invariant

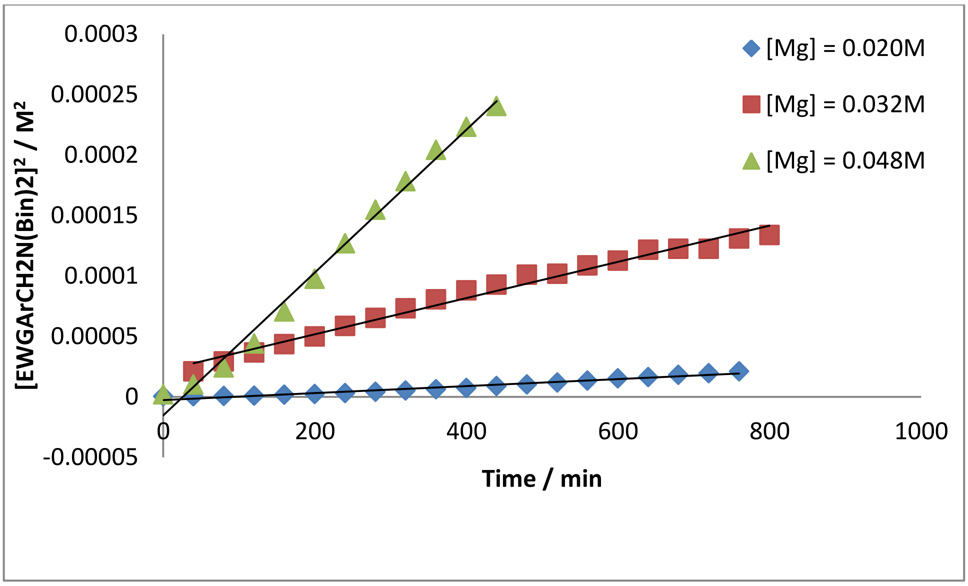

	[Mg] 0.020M	
	Value	Error
m_1	-2.68E-06	5.97E-07
m_2	2.90E-08	1.34E-09
Chisq	0.192125	n/a
R^2	0.962262	n/a

	[Mg] 0.032M	
	Value	Error
m_1	2.17E-05	2.23E-06
m_2	1.50E-07	4.64E-09
Chisq	0.038156	n/a
R^2	0.983011	n/a

	[Mg] 0.048M	
	Value	Error
m_1	-1.53E-05	4.56E-06
m_2	5.90E-07	1.75E-08
Chisq	0.04374	n/a
R^2	0.991255	n/a

Figure S99. [Mg] vs k_{obs}; non-linear fit

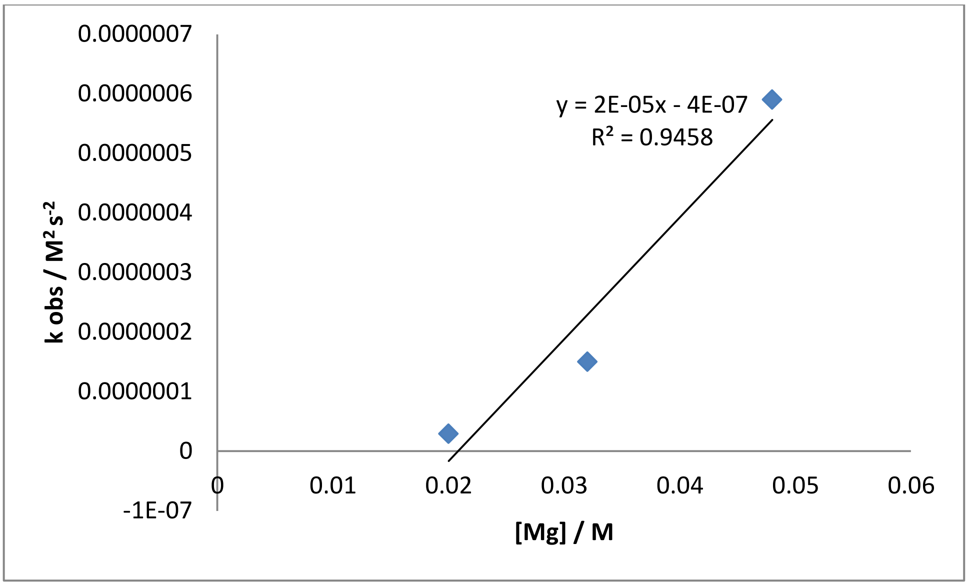

Figure S100. $[Mg]^2$ vs k_{obs}; non-linear fit

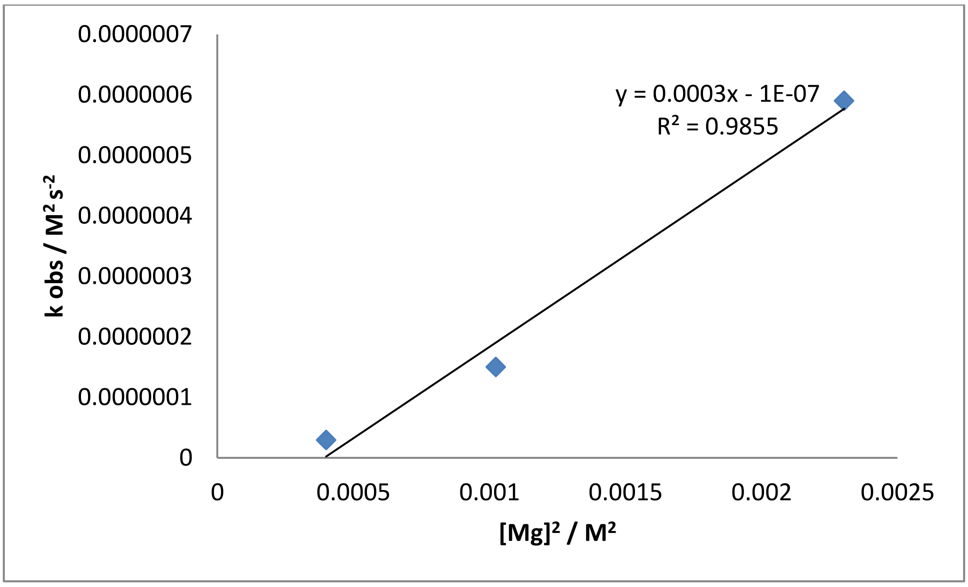

Figure S101. $[Mg]^3$ vs k_{obs}; indicating 3rd order dependence on [Mg] under *pseudo*-first order conditions in [*m*-$MeOC_6H_4CN$]

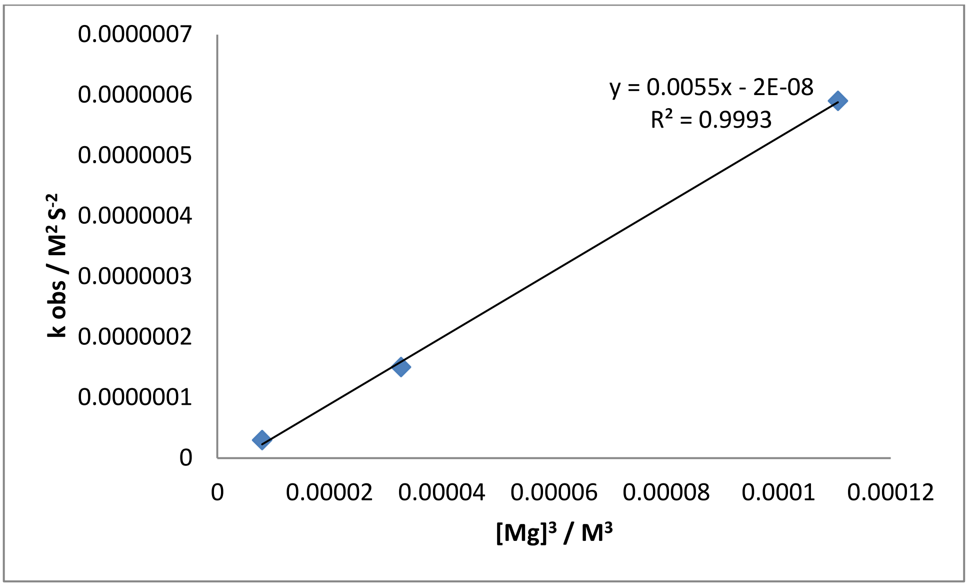

	Value	Error
m_1	-2.20E-08	9.96E-09
m_2	0.005513	0.000149
Chisq	0.000819	n/a
R^2	0.999268	n/a

Variable Temperature Studies

Figure S102. [*m*-$MeOC_6H_4CN$] vs time; non-linear kinetics

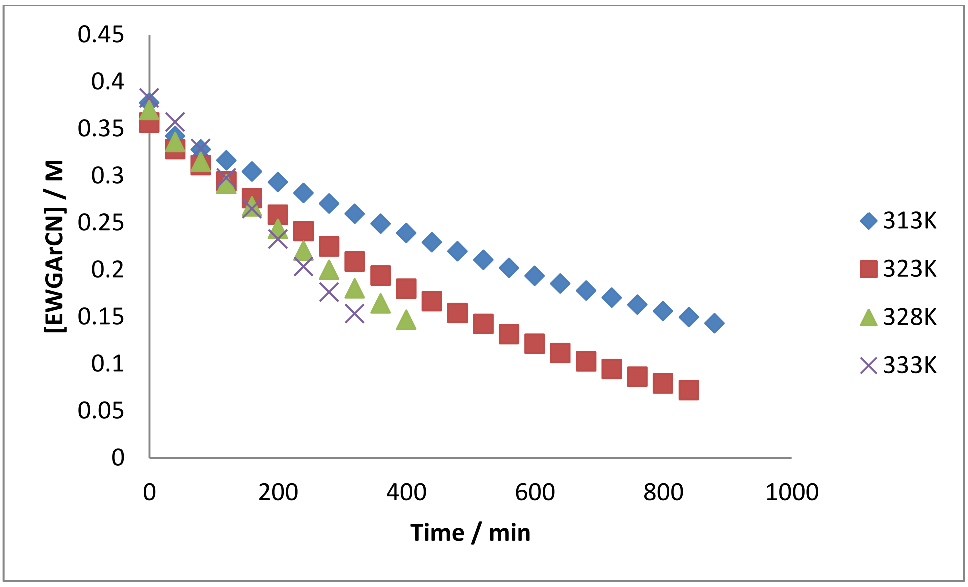

Figure S103. ln([*m*-$MeOC_6H_4CN$]$_0$/[*m*-$MeOC_6H_4CN$]$_t$) vs time; variable temperature under standard reaction conditions

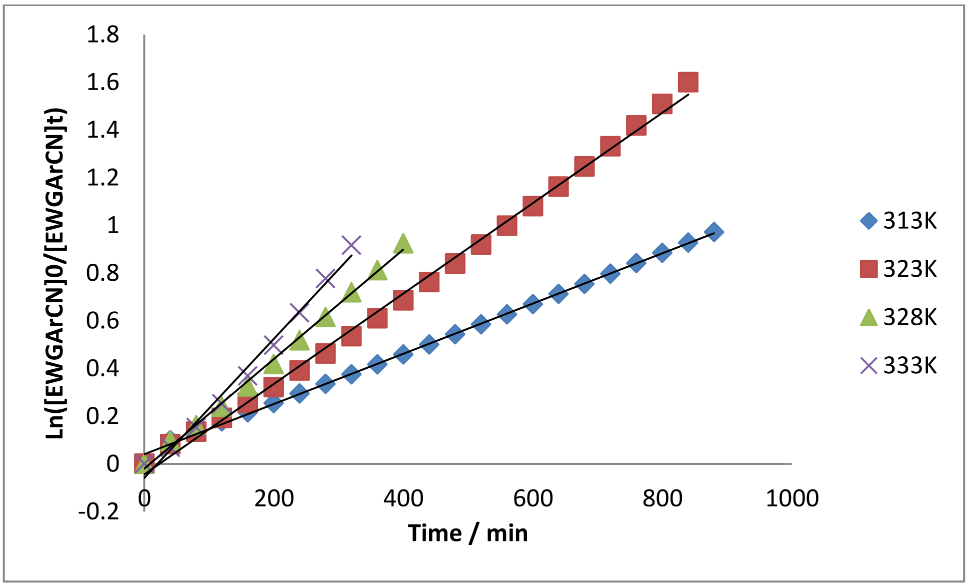

	313 K	
	Value	Error
m_1	0.039620	0.004333
m_2	0.001050	0.000008
Chisq	0.02007	n/a
R^2	0.99866	n/a

	323 K	
	Value	Error
m_1	-0.040080	0.011011
m_2	0.001880	0.000024
Chisq	0.00774	n/a
R^2	0.99702	n/a

	328 K	
	Value	Error
m_1	-0.020310	0.010620
m_2	0.002300	0.000045
Chisq	0.004877	n/a
R^2	0.9966	n/a

	333 K	
	Value	Error
m_1	-0.059510	0.023468
m_2	0.002920	0.000123
Chisq	0.038538	n/a
R^2	0.98766	n/a

Figure S104. 1/ *m*-MeOC$_6$H$_4$CN] vs time; non-linear kinetics

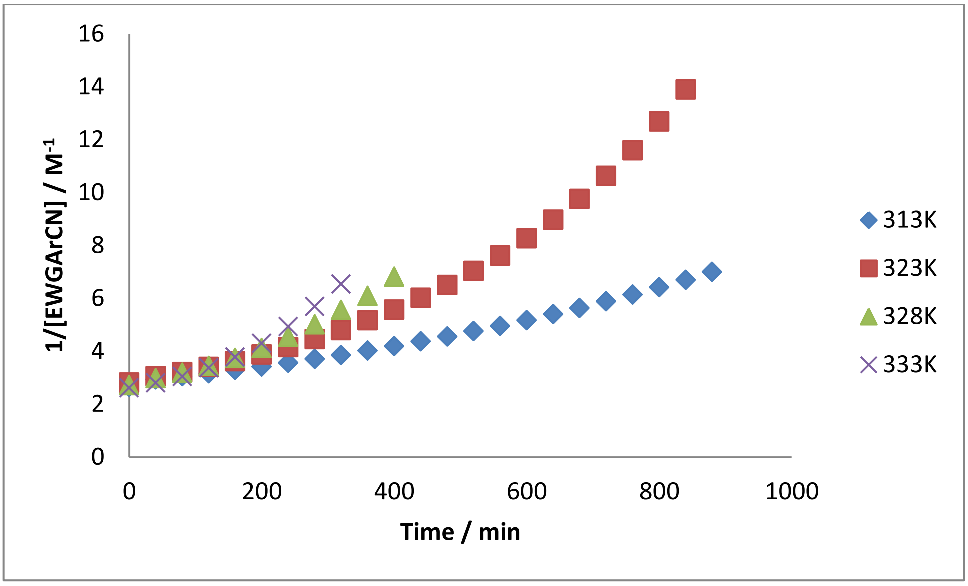

Figure S105. Eyring Plot; 1/T vs Ln(k/T)

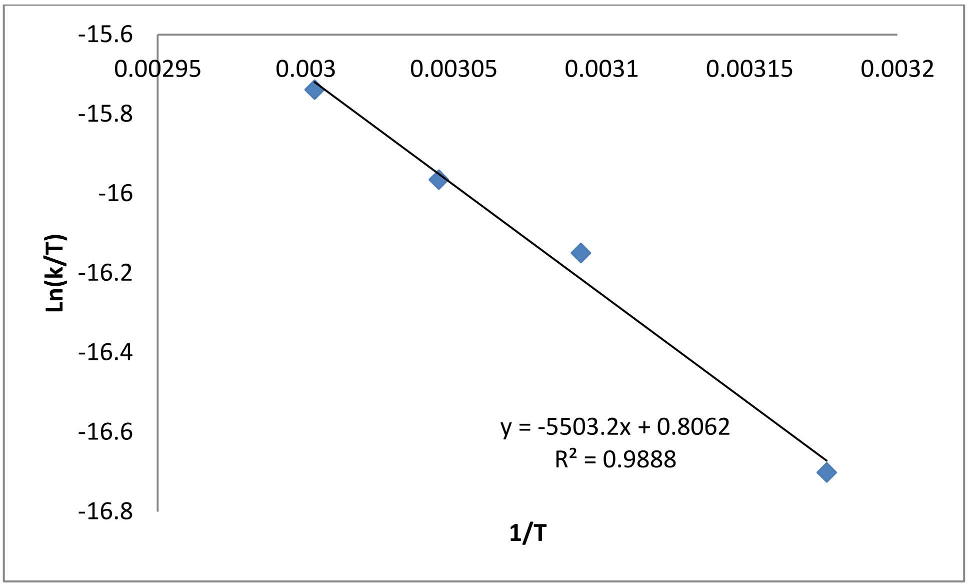

	Value	Error
m_1	0.806183	1.277090
m_2	-5503.171097	414.646769
Chisq	0.007110758	n/a
R^2	0.988773	n/a

This graph was used to calculate the following Activation Energy Parameters, least square error analysis was also carried to provide accurate error information.

	Value	Error
ΔH	45.75 kJ mol^{-1}	± 3.44
ΔS	-190.84 J k^{-1} mol^{-1}	± 10.61

Figure S106. Arrhenius Plot; 1/T vs Ln(k)

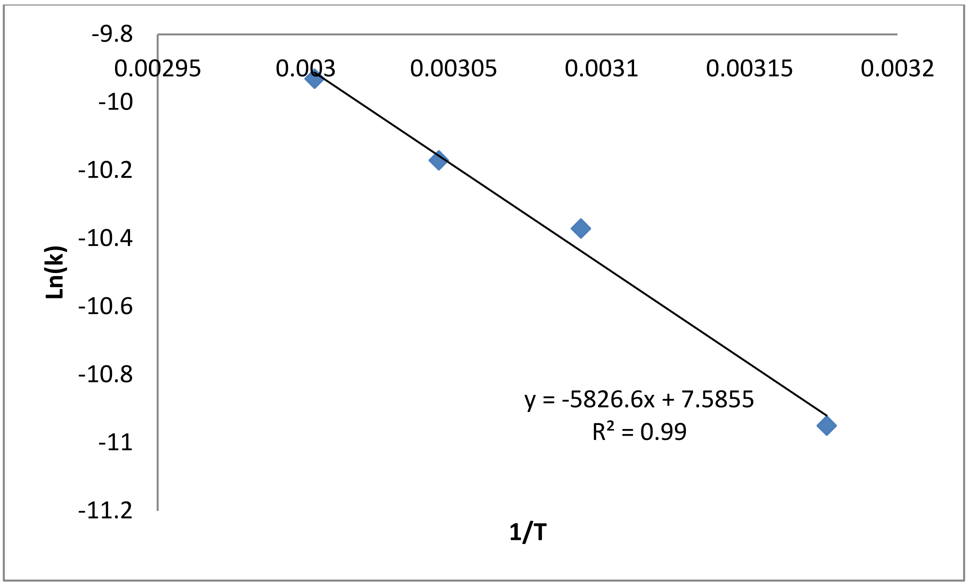

	Value	Error
m_1	7.585536	1.272382
m_2	-5826.648141	413.118121
Chisq	0.006290611	n/a
R^2	0.990046	n/a

This graph was used to calculate the following Activation Energy Parameter; least square error analysis was also carried to provide accurate error information.

	Value	Error
Ea	48.44 kJ mol^{-1}	± 3.43

Kinetic Isotope Effect - EtCN

Using the standard reaction: LMgBu (10 mg, 0.02 mmol ie. 10 mol%) was dissolved in 0.5 ml of C_6D_6, 110.5 μL (0.42 mmol) of deuterated pinacolborane was then added followed by 0.2 mmol of propionitrile. ^{1}H NMR spectra were collected at consistent intervals until the reaction reached the desired 3 half-lives (80 % product conversion). All reactions were carried out at 323 K.

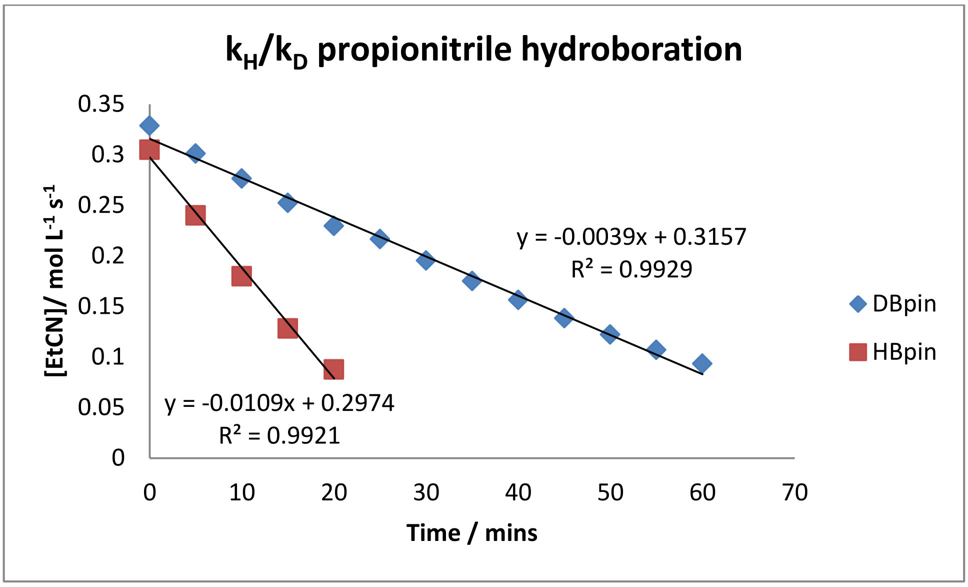

Figure S107: Determination of KIE for propionitrile dihydroboration

$K_H/k_D = 0.0109/0.0039 = 2.79$

*Kinetic Isotope Effect – p-*MeOC_6H_4*CN*

Using the standard reaction: LMgBu (10 mg, 0.02 mmol ie. 10 mol%) was dissolved in 0.5 ml of C_6D_6, 110.5 µL (0.42 mmol) of deuterated pinacolborane was then added followed by 0.2 mmol of 4-methoxybenzonitrile. ^{1}H NMR spectra were collected at consistent intervals until the reaction reached the desired 3 half-lives (80 % product conversion). All reactions were carried out at 323 K.

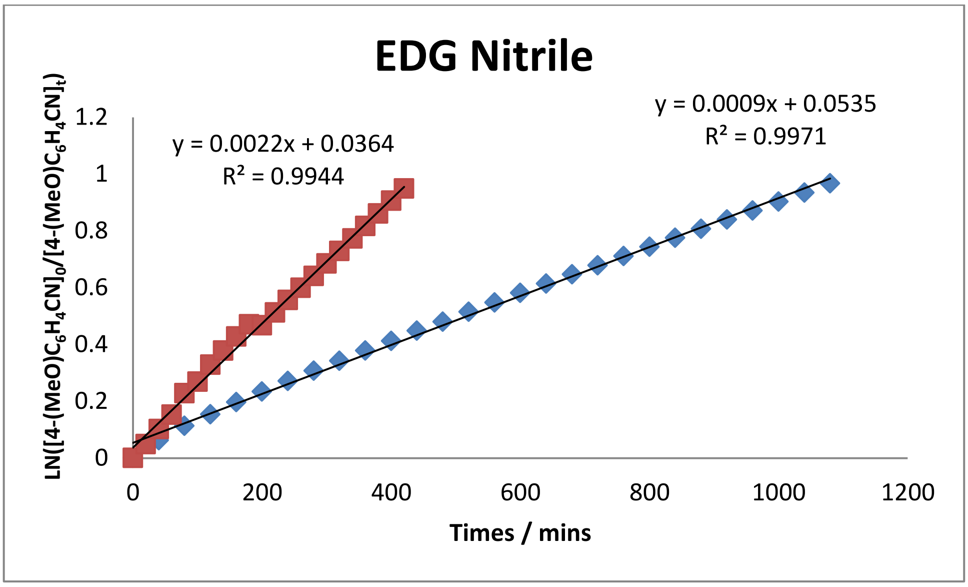

Figure S108: Determination of KIE for (4-methoxy)benzonitrile dihydroboration.

Kinetic Isotope Effect - *m*-$MeOC_6H_4$*CN*

Using the standard reaction: LMgBu (10 mg, 0.02 mmol ie. 10 mol%) was dissolved in 0.5 ml of C_6D_6, 110.5 µL (0.42 mmol) of deuterated pinacolborane was then added followed by 0.2 mmol of 3-methoxybenzonitrile. ^{1}H NMR spectra were collected at consistent intervals until the reaction reached the desired 3 half-lives (80 % product conversion). All reactions were carried out at 323 K.

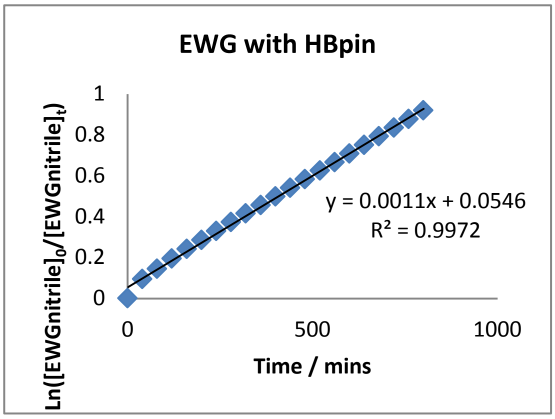

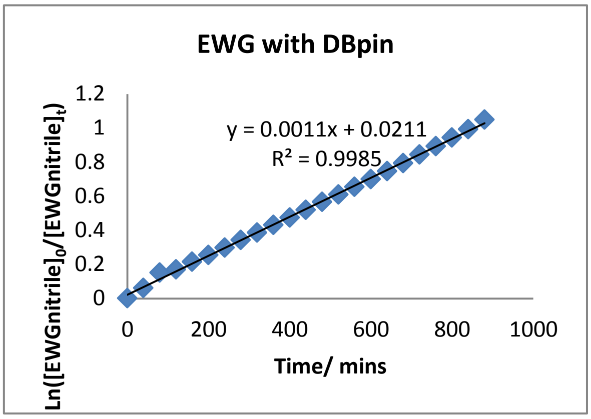

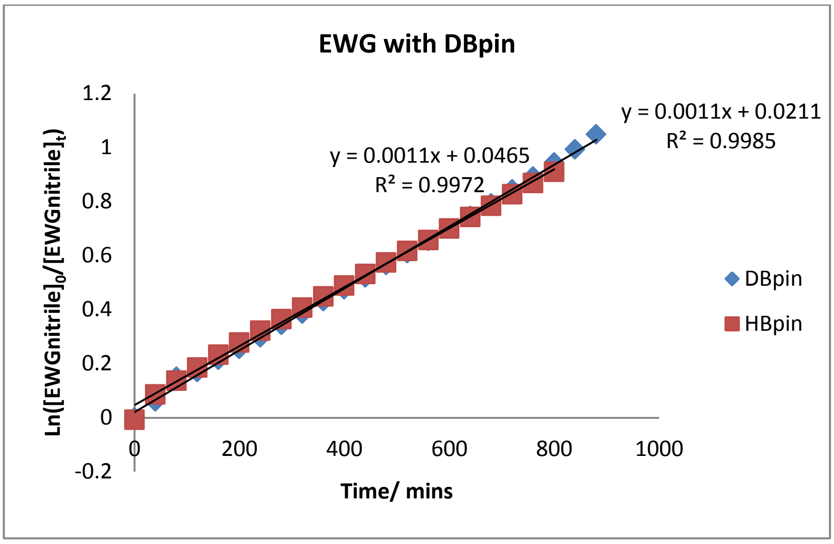

Figure S109: Determination of KIE for (3-methoxy)benzonitrile dihydroboration.

KIE = 1
